# Multistep CO_2_ Activation and Dissociation Mechanisms on Pd_*x*_Pt_4–*x*_ Clusters in the Gas Phase

**DOI:** 10.1021/acs.jpca.2c08333

**Published:** 2023-05-17

**Authors:** Renata Sechi, Tibor Höltzl

**Affiliations:** † Department of Inorganic and Analytical Chemistry, Budapest University of Technology and Economics, Szent Gellért tér 4, Budapest 1111, Hungary; ‡ ELKH-BME Computation Driven Chemistry Research Group, Department of Inorganic and Analytical Chemistry, Budapest University of Technology and Economics, Műegyetem rkp. 3, Budapest 1111, Hungary; ¶ Furukawa Electric Institute of Technology, Késmárk utca 28/A, Budapest 1158, Hungary

## Abstract

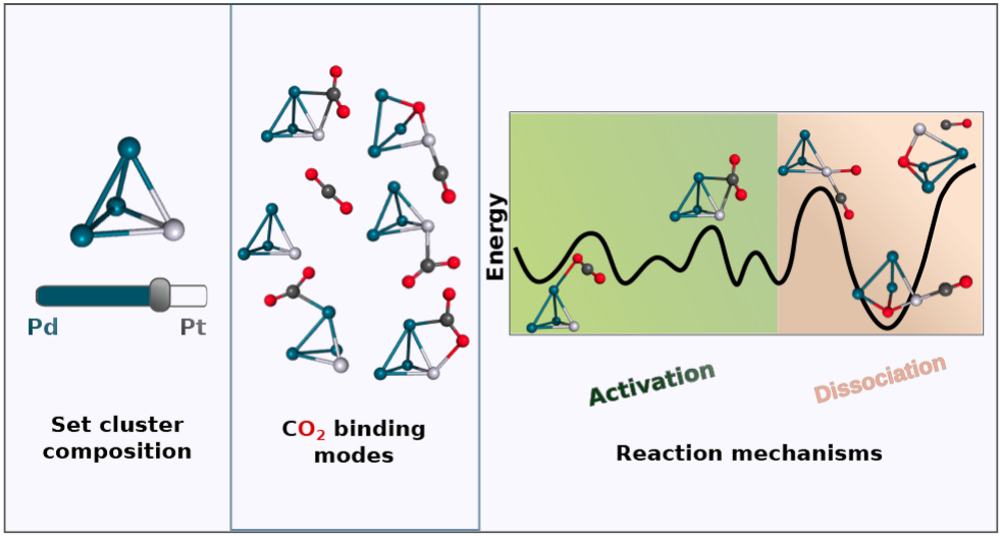

Palladium, platinum, and their alloys are promising catalysts for electrochemical CO_2_ reduction reactions (CO_2_RR), leading to the design of durable and efficient catalysts for the production of useful chemicals in a more sustainable way. However, a deep understanding of the CO_2_RR mechanisms is still challenging because of the complexity and the factors influencing the system. The purpose of this study is to investigate at the atomic scale the first steps of the CO_2_RR, CO_2_ activation and dissociation mechanisms on Pd_*x*_Pt_4–*x*_ clusters in the gas phase. To do it, we use Density Functional Theory (DFT)-based reaction path and ab initio molecular dynamics (AIMD) computations. Our research focuses on the description of CO_2_ activation and dissociation processes via the computation of multistep reaction paths, providing insights into the site and binding mode dependent reactivity. Detailed understanding of the CO_2_–cluster interaction mechanisms and estimating of the reaction energy barriers facilitate comprehension of why and how catalysts are poisoned and identification of the most stable activated adducts configurations. We show that increasing the platinum content induces fluxional behavior of the cluster structure and biases CO_2_ dissociation; in fact, our computations unveiled several dissociated CO_2_ isomers that are very stable and that there are various isomerization processes leading to a dissociated structure (possibly a CO poisoned state) from an intactly bound CO_2_ one (activated state). On the basis of the comparison of the Pd_*x*_Pt_4–*x*_ reaction paths, we can observe the promising catalytic activity of Pd_3_Pt in the studied context. Not only does this cluster composition favor CO_2_ activation against dissociation (thereby expected to facilitate the hydrogenation reactions of CO_2_), the potential energy surface (PES) is very flat among activated CO_2_ isomers.

## Introduction

Increasing anthropogenic CO_2_ emissions are directly responsible for global warming and climate change,^1−4^ and currently, several methods and techniques for reducing CO_2_ emissions into the atmosphere have been investigated.^5−7^ The conversion of carbon dioxide into valuable chemicals via electro- or thermal catalysis^8^ is a promising technique; hence, there is a significant need for developing and enhancing the efficiency of catalytic materials.

Among the different CO_2_ reduction catalysts, palladium,^9^ platinum,^10^ and their alloys^11^ attracted large interest as these yield formate or carbon monoxide with high efficiency. Performing catalysis employing platinum and palladium nanoparticles is an attractive approach to enhance the utilization and manage the scarce availability of these materials, to help avoid catalyst deactivation,^12^ and also to suppress the undesired hydrogen evolution reaction.^13,14^ Chen et al. showed how the alloying of palladium and platinum inhibits CO poisoning during electrocatalytic formic acid oxidation and CO_2_ reduction.^15^ Zhang et al. screened the effect of the PdPt nanoparticle composition on formic acid electrocatalytic oxidation and concluded that alloying 10% platinum gives the highest activity, where the CO poisoning was suppressed by the distant platinum sites.^16^ Recently, Kortlever et al. studied the properties of carbon-supported Pd_*x*_Pt_100–*x*_ nanoparticles for CO_2_ electroreduction to formic acid and found that Pd_70_Pt_30_ shows a faradaic efficiency of 88%.^17^ The activity of PdPt aerogels with Pd/Pt atomic ratios from 50:50 to 90:10 has been recently studied by Herranz et al.^13^ As no carbon support is needed for CO_2_RR using the aerogel catalyst, the hydrogen evolution reaction is considerably suppressed.

Small metal clusters have been shown to activate CO_2_ and catalyze its dissociation^18−22^ or conversion to various chemicals.^23−25^ CO_2_ activation and dissociation mechanisms have been studied using Density Functional Theory (DFT)-based methods for Pt_4_^26−28^ and Pd_4_^18,28^ clusters in the gas phase, and also for tetranuclear mono- and bimetallic Pd_*x*_Pt_4–*x*_ clusters on a surface.^29,^ Wang et al. compared the energy profiles of the CO_2_ dissociation mechanism on Pd_*x*_Pt_4–*x*_/In_2_O_3_.^29^ By comparing the cluster composition-dependent CO_2_ dissociation energy barriers, they found that it decreases in the order of Pd_4_ > Pd_3_Pt > Pd_2_Pt_2_ > PdPt_3_ > Pt_4_. Additionally, the Brønsted–Evans–Polanyi relation is satisfied by the good correlation between the CO_2_ dissociation barrier and the corresponding reaction energy. They concluded that the Pd_2_Pt_2_/In_2_O_3_ cluster is the optimal composition for the CO_2_ activation and dissociation since it balances these two processes.

Small gas-phase clusters are also valuable model systems of complex catalyst materials.^22,31,32^ For example, it has been shown that the oxidation mechanism of carbon monoxide on small Pd_*n*_^+^ (*n* = 2–6) clusters is similar to that on palladium single crystals,^33^ while CO has been found to bind more weakly to Pd_6_^+^ than to the other cluster sizes or to the palladium surface, indicating its enhanced resistance against poisoning. Doping platinum with another element in order to reduce the binding strength has been seen to be an efficient way of mitigating CO poisoning, as has been unveiled in the case of GePt_*n*–1_ (*n* = 5–9) clusters.^34^ The CO_2_ electroreduction mechanism was also studied on Pt_4_ and Pd_4_ clusters^28^ using DFT methods and the computational hydrogen electrode model.

In this context, the role of electronic structure computations has been of great importance in the past years,^35−37^ supporting catalyst design by detailed understanding of the reaction mechanisms and the electronic structure of the participating chemical species. Exploration of multistep reaction mechanisms has contributed significantly in the unraveling of the role of the atomic catalyst, as in the case of the hydroformylation reaction on Rh/CoO,^38^ the oxygen evolution reaction promoted by cobalt,^39^ and also the CO_2_ reduction reactions, e.g., on Ni/C,^40^ on copper,^41,42^ or on platinum^43^ surfaces, or the full methanol synthesis reaction path on Pd_4_/In_2_O_3_^44^ and on Pt_8_/In_2_O_3_.^45^ Considering these reactions as complex and multistep processes, as well as the analysis of the electronic structures of the intermediates and transition structures, opens the way toward rational catalyst design.^46,47^ The preferred CO_2_ binding mode and its dissociation is a particularly interesting question as it can change the reaction mechanism and also the preferred product. For example, theoretical computations showed thermodynamically unfavored CO_2_ dissociation on Pt(111) or Pd(111) surfaces,^48^ while, size-dependent preference of CO_2_ toward activation or dissociation has been observed on Pt_*n*_^–^ (*n* = 4–7) small gas-phase cluster anions.^20^ It was also observed that CO_2_ does not dissociate on a Pd/Al_2_O_3_ catalyst and adsorbed hydrogens are needed for formation of CO.^49^

In this work, we use DFT methods to study CO_2_ binding, activation and dissociation on the Pd_*x*_Pt_4–__*x*_ clusters. Up to now, both the experimental and the computational results considered only single-step reaction paths for this class. Using gas-phase model clusters, we show that the reactions involve several steps and that the dynamics play an important role. As a first step, we analyze the bare cluster geometries; then we systematically investigate the CO_2_ binding modes to the clusters. Considering Pd_*x*_Pt_4–*x*_ systems in the gas phase allows one to observe different energy profiles for reaction mechanisms as a function of the cluster composition and under well-defined conditions. Finally, we interpret the chemical bonding based on Mayer bond orders (MBOs) and energy decomposition analysis (EDA). Stable adducts are used to construct CO_2_ activation and dissociation reaction paths. The reactivities are analyzed further by performing ab initio molecular dynamics (AIMD) simulations.

## Computational Methods

The PBE functional^50^ and the Grimme’s D3 dispersion correction^51^ have been used for all of the computations. The PBE functional has been applied previously to Pd or Pt clusters, Pd/Pt bimetallic alloys, and surfaces.^26,29,30,52−56^ Moreover, this choice of functional is consistent with the previously reported results of small-size clusters to surfaces and nanoparticles.^57,58^ For selected cases, we also investigate the effect of the exchange-correlation functional on the results. The bare cluster geometries were gradually converged in three steps using three different basis sets: LANL2DZ,^59^ def2-SVP, and def2-TZVP, respectively.^60^ The energy analysis of the different basis sets can be found in the Supporting Information (SI), Figures S2–S12. We also tested the accuracy of the computations compared to coupled cluster references (see the SI for details).

We verified the stability of the self consistent field (SCF) solutions by performing internal stability analysis using the Davidson’s algorithm.^61^ Harmonic vibrational analysis of the stationary points on the potential energy surface was performed to determine if the obtained structures are minima or transition structures. We applied the freezing-string method^62^ for generating transition-state structure guesses. Geometries of the located stationary points were subsequently refined using the eigenvector following algorithm.^63,64^ The intrinsic reaction paths were computed starting from each transition structure to confirm the connecting reactant and product geometries.

AIMD simulations allowed us to compare the trajectories with the insight gained from the reaction path computations. Unless noted otherwise, the first adducts on the reaction paths were used as starting structures for the simulations. We computed the trajectory with GPAW^65^ using the PBE functional and TZP basis set, integrated in the atomic simulation environment (ASE).^66^ The simulations were carried on an *NVT* ensemble and by solving the Langevin equation. The algorithm for the integration of the Langevin equation is accurate to second order.^67^ We chose the Langevin dynamics in order to mitigate the flying ice cube effect.^68^ We set the simulation at temperature *T* = 500 K, friction coefficient γ = 0.01, and time step 1 fs. The higher temperature helps sample the atomic configuration space during the typically achievable time scales of molecular dynamics simulations. Each simulation was run for 10 ps.

DFT computations and EDA were performed using the Q-Chem 5.4 software,^69^ while the MBOs were computed using the Multiwfn code.^70^ All of the figures were generated using the PyMol^71^ and IQmol programs. For all of our graphs we used universally readable scientific colormaps from the Python package cmcrameri.^72^

## Results

### Bare Pd_*x*_Pt_4–*x*_ Clusters

We carried out a systematic search for the stable bare cluster geometries, similarly to the approach described in refs (18), (26), and (73). We selected the most stable structures after optimizing the geometries of several possible initial geometries (linear, triangular, quadrangular, and tetrahedral) and also several initial spin multiplicities. A detailed description is given in the SI. Furthermore, we screened the bare cluster structures for the singlet, triplet, and quintet spin multiplicities, as done for instance in ref (30).

The ground state of the Pd_*x*_Pt_4-*x*_ clusters is a tetrahedron-like structure, triplet spin, in agreement with previous works.^26,30,74−76^ The details, including the relative energies of the higher spin states, are available in the SI. Since, previously, quadrangular quintet^27^ and tetrahedral triplet^26^ states of the Pt_4_ cluster were reported to be close in energy, we performed further computations using various density functionals (B3LYP, TPSS, TPSSh) and basis sets (def2-TZVP, def2-TZVPP, def2-QZVP, def2-QZVPP) to test the results obtained using the PBE functional (see the SI for details). All of the computations indicated that the ground state of Pt_4_ is a tetrahedral-shaped triplet, in agreement with previous reports. The tetrahedral, triplet lowest-energy state is further confirmed by our CCSD(T)/def2-QZVPPD single point computations on the PBE/def2-TZVP geometries. However, the *T*_1_ diagnostics of the CCSD(T) computations are large (in the range of 0.1–0.2), showing possible multireference characters (see the SI for the details).

**Figure 1:**
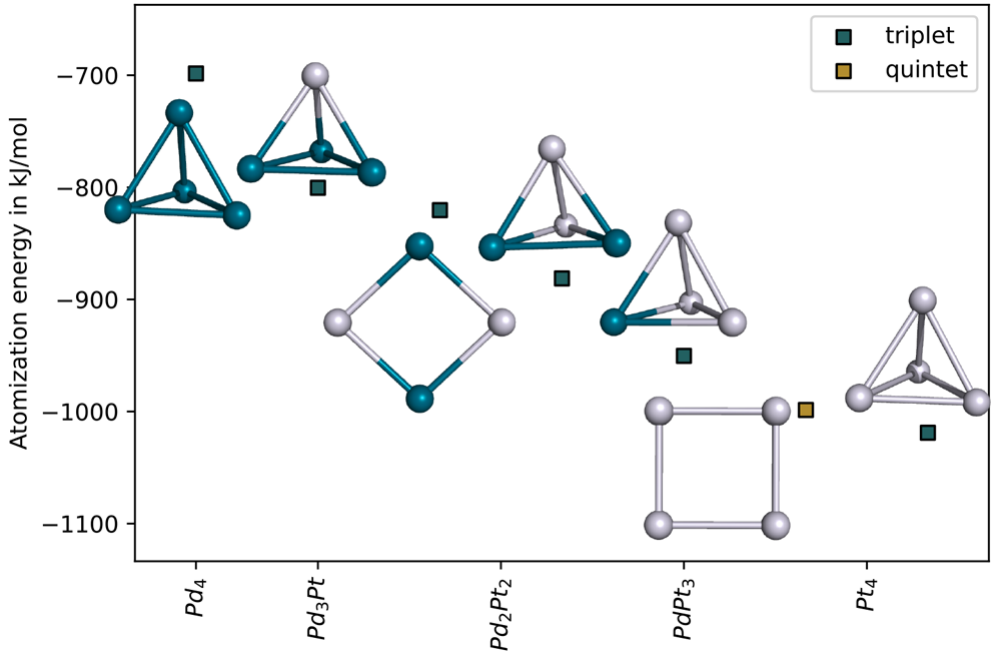
Lowest-energy structures obtained by screening several geometries and spin states. The reported energies are computed using the PBE/def2-TZVP level of theory. Colors: blue - palladium; ivory - platinum.

As all of the computations agree on the tetrahedron-shaped, triplet ground state of the Pt_4_ cluster, we use this structure and spin state in our further computations. The atomization energies of the tetrahedron-like clusters depend on Pd content, with order Pd_4_ > Pd_3_Pt > Pd_2_Pt_2_ > PdPt_3_ > Pt_4_. Taking the tetrahedron-like structures in Figure 1, we notice that the atomization energies change nonlinearly with the cluster composition; the atomization energy is more negative by ∼100 kJ/mol upon single platinum substitution in the Pd_4_ cluster, whereas there is a smaller atomization energy difference between the Pd_2_Pt_2_ and the PdPt_3_ clusters.

### CO_2_ Binding Modes

We systematically generated several starting structures of CO_2_–cluster adducts with different CO_2_ binding modes, using the method applied in our previous works.^77−79^ The initial geometries include both intact and dissociated CO_2_ molecules (see Figures S16 and S17 in the SI). Computations with different spin multiplicities (singlet, triplet, quintet) of the Pd_*x*_Pt_4–*x*_ + CO_2_ clusters inferred that the adducts are preferably triplet structures, as discussed in the SI.

We compute the adsorption energy *E*_ads_
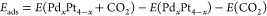
1for each adduct structure. In agreement with previous studies of Pd_*x*_Pt_4–*x*_–CO_2_ adducts on a support,^29,30^ our results show that the adsorption energy decreases monotonically with the Pt content; this is true also for the atomization energies of the bare clusters. However, the composition-dependent monotonous decrease of the atomization and adsorption energies is not maintained by every alloy cluster of Pd or Pt, as, for example, the case of Cu_*x*_Pd_4–__*x*_ in ref (18) or of Cu_*x*_Pt_4–*x*_.^26^

For all cluster compositions, adducts with dissociated CO_2_ were found to have low energy; however, for the bimetallic Pd_2_Pt_2_ and PdPt_3_ clusters, several energetically close-lying, low-energy adducts were found (see Figures S20 and S21 in the SI). In all of the bimetallic-cluster compositions, the cluster can distort and rearrange from the tetrahedron-like geometry and open its structure into a bent-rhomboidal shape. This takes place more easily with increasing platinum content (see Figures S18–S22 in the SI).

### Analysis of the CO_2_–Cluster binding

Structural and geometrical descriptors, such as O–C–O angles, C–O bond lengths, and C–M (M denotes platinum or palladium) distances, have been computed to characterize the carbon dioxide activation. Nevertheless, these descriptors are not sufficient to fully understand the CO_2_ binding mechanism as, e.g., we observed adducts with similar geometries and yet very different adsorption energies. This shows that, apart from the geometry, electronic effects also play an important role in the binding. Thus, we computed the natural charges and MBOs^80,81^ and performed also EDA to understand the CO_2_ activation in these clusters.

#### MBO

**Figure 2:**
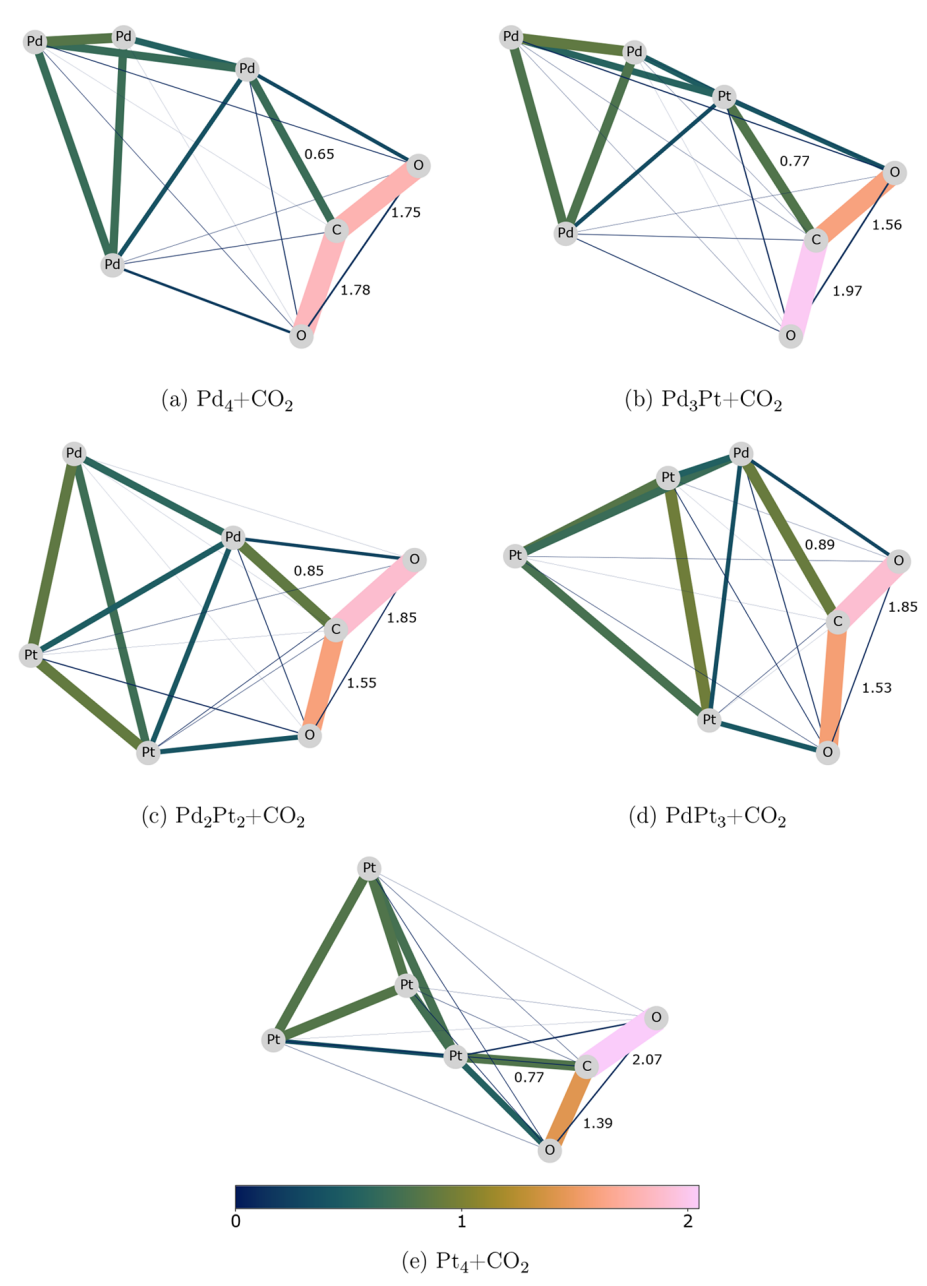
MBOs computed for representative Pd_*x*_Pt_4–*x*_ adducts. The edge width and color indicate the MBO values between the adjacent atoms.

The MBO has already been adopted in the analysis of CO_2_ activation on small Cu–Zr bimetallic clusters,^82^ showing its correlation with the bonding energy. In our study, the MBOs show stronger CO_2_ binding to the platinum than to the palladium atoms. Figure 2 shows the MBOs of representative cluster–CO_2_ adducts. With the pure palladium cluster in Figure 2a, the MBOs between the C–O bonds are similar, showing that the Pd–C binding does not change the electron distribution significantly. The difference between the MBO values of the carbon–oxygen bonds enlarges proportionally to the platinum content in the cluster. The disparity is even greater if the carbon binds to a platinum atom. In alloy structures in which palladium binds to the carbon, the high MBO of 0.7 indicates strong binding of oxygen to the neighboring platinum. The MBOs between the Pd/Pt and carbon are larger than those between Pd/Pt and oxygen, and this indicates that the bond covalent character is stronger in the first case. Interestingly, in these structures, the enhanced Pt–O interaction decreases the Pt–Pd MBO, so that the Pt–Pd MBO is smaller than the Pt–O MBO. For Pt_4_ + CO_2_ adducts, the two carbon–oxygen bonds are affected the most by the presence of the metal cluster; this means that the platinum atom modifies significantly the electronic population of the CO_2_. However, the MBOs give information about the bonds’ covalent character, but charge transfer, partial ionic characters, or noncovalent interactions can also contribute to the binding in these clusters.^83,84^ Thus, we analyzed further the interactions using the EDA.

#### EDA

EDA decomposes quantitatively the intermolecular interaction energy into different terms with clear chemical and physical meaning.^85−89^ This makes it possible to determine the contribution of the different interactions to intermolecular binding^86^ between the metal cluster and the CO_2_ in the adducts.

Our results were computed using a version of EDA based on absolutely localized molecular orbitals (ALMO-EDA).^86^ ALMOs are molecular orbitals expanded in terms of atomic orbitals of a specific fragment for the given molecule. The EDA version adopted in our calculations^86,90,91^ fragments the interaction energy in the adduct, *E*_int_ into
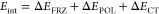
2The frozen density component, Δ*E*_FRZ_, is the energy change needed to bring the infinitely separated rigid fragments into the adduct without any relaxation of the molecular orbitals on the fragments and thus involves the electrostatics, Pauli repulsion, and the van der Waals attraction between the fragments. The polarization energy, Δ*E*_POL_, is computed as the energy lowering due to the intramolecular relaxation of each fragment’s ALMOs in the field of all other fragments in the system. The charge transfer energy term, Δ*E*_CT_, examines the energy lowering due to electron transfer from occupied orbitals on one fragment to virtual orbitals of another fragment.

A characteristic of the ALMO-EDA is that the charge transfer term can be decomposed *exactly* into donor and acceptor contributions represented by complementary occupied virtual pair (COVP) orbitals. All of the COVPs together describe the charge transfer between the fragments exactly.^86,90,91^ Major contribution to the bonding between the fragments is often given by only a few of these pairs, so it is usually sufficient to consider only those to qualitatively interpret the contribution of the charge transfer interactions to the intermolecular binding. Recently, COVPs have been used successfully to explain the CO_2_ binding on doped copper clusters^79^ and also to explain copper cluster binding to boron-doped graphene.^92^

We defined the tetranuclear Pd_*x*_Pt_4–*x*_ as one fragment and the CO_2_ as the second fragment. Our EDA was limited to the adducts with intact CO_2_. The fragmentation of the adducts with dissociated CO_2_ is a complicated task since the definition of the fragments is determinant for the results.

**Figure 3:**
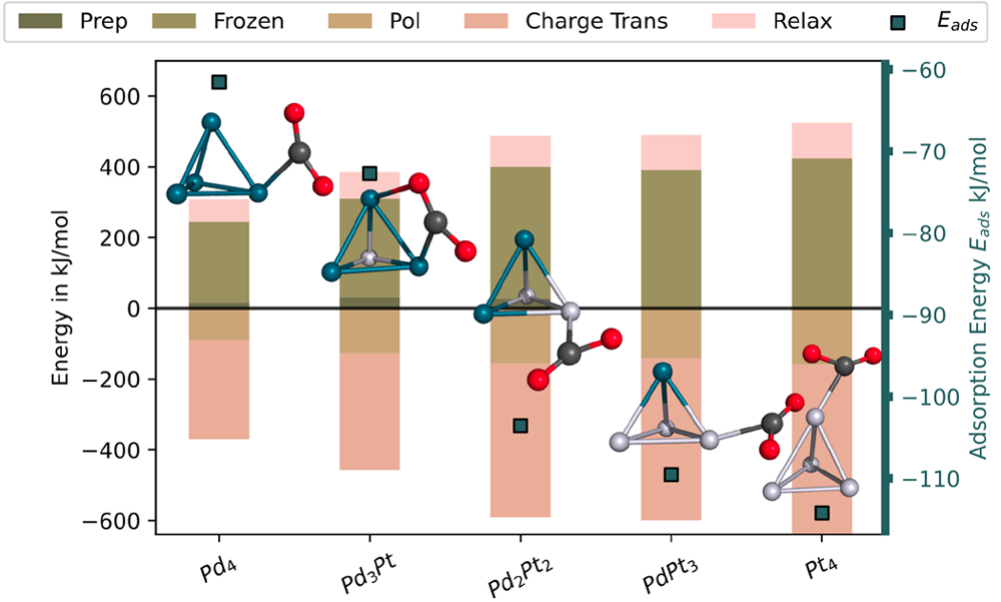
EDA of the lowest-energy intact CO_2_ adducts for each cluster composition.

In Figure 3, we included the preparation energy *E*_prep_ and the relaxation energy *E*_rel_ as well. The preparation energy *E*_prep_ represents a usually small energy contribution, which cannot be decomposed to either frozen, polarization, or charge transfer energies. The relaxation energy is *E*_rel_ is computed by
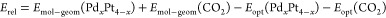
3where *E*_mol–geom_ is the energy of the molecular fragment with the same geometry of the adduct and *E*_opt_ is the energy of the optimized structure of the free fragments.

**Figure 4:**
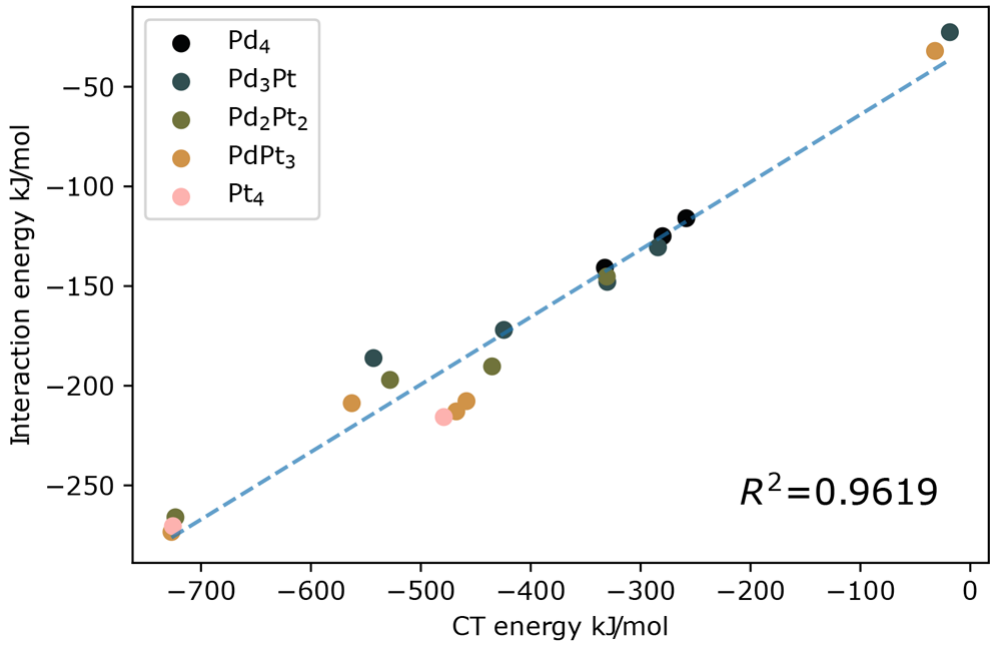
Correlation between the charge transfer (CT) energy and the interaction energy (eq 2) of the different intact CO_2_–cluster adducts.

Thus, the adsorption energy can be computed as
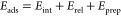
4The charge transfer scales linearly with the interaction energy (defined as in eq 2), as shown by Figure 4. This clearly shows the importance of the charge transfer in the CO_2_–cluster binding. We observed no correlation between the charge transfer and the HOMO or LUMO energies or with the dipole moment of the bare cluster (see the SI for more details); thus, we carried out the COVP analysis to interpret the results. On the other hand, the charge transfer energy does not correlate with the adsorption energy. This is probably due to the fact that the adduct’s fragments can have very different geometries from the optimized free fragments, and so there is no relation between the free fragment and adduct conformation of each fragment. That is why the contribution of the relaxation energy does not scale with the charge transfer, which is associated only to the characteristics of the fragments in the adduct geometry. Also, for some structures the preparation energy has a larger contribution.

**Figure 5:**
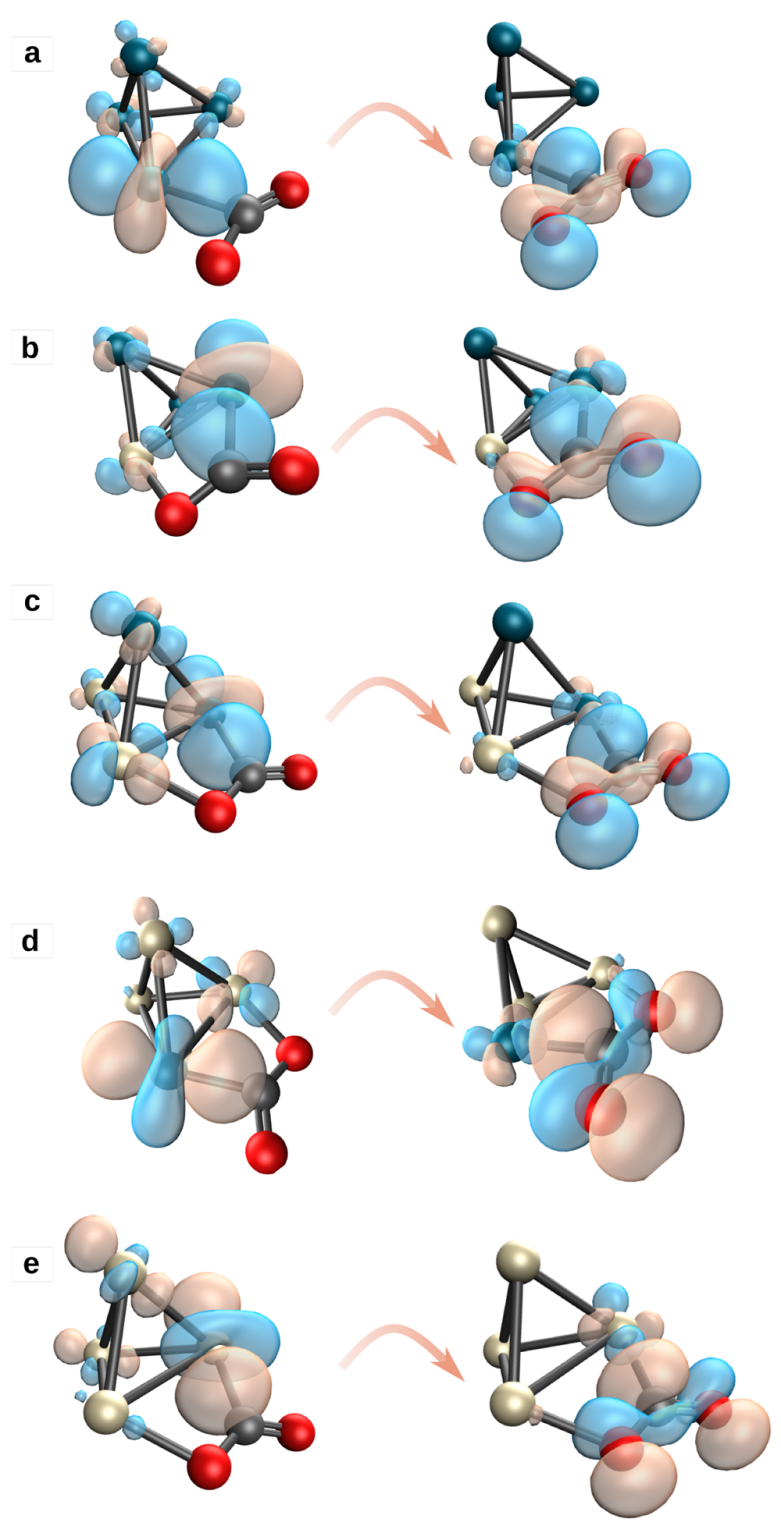
Major COVPs for Pd_*x*_Pt_4–*x*_ clusters, ordered from a to e by decreasing palladium content.

The results of the COVP analysis (see Figure 5) infer that the d_*z*^2^_ orbital of one of the transition metal atoms donates an electron to the antibonding 2π* orbital of CO_2_. A similar electron donation mechanism has been found also in doped copper clusters^25^ and in platinum-doped gold clusters.^93^ This charge transfer has a remarkable role in the activation of the CO_2_ molecule. When the d_*z*^2^_ orbital mixes with 2π* of CO_2_, the antibonding 2π* is populated, and thus, the repulsion between the C–O atoms increases. The repulsion elongates the C–O bond, as was observed also in refs (94) and (95). The CO_2_ molecule bends into a V-shape, which indicates an activated state. Thus, the electron donation from the d orbitals of the cluster atoms to the antibonding π* orbital of the CO_2_ molecule is responsible for the activation.

### Reaction Paths

We investigated the reactions starting from the located intact CO_2_ bound clusters. Our relaxed energy scans of the C–M distance (M denotes the nearest Pd or Pt atom to the carbon) showed barrier-free adduct formation of the CO_2_ molecule and the Pd_*x*_Pt_4–*x*_ clusters in the first reaction step. For the reaction paths involving Pd_4_, Pd_2_Pt_2_ and Pt_4_, the initial adduct of the reaction paths shows CO_2_ chemisorption. However, for Pd_3_Pt and PdPt_3_ clusters, our adduct screening revealed stable van der Waals structures as well, which were considered as starting geometries of the reaction paths.

**Figure 6:**
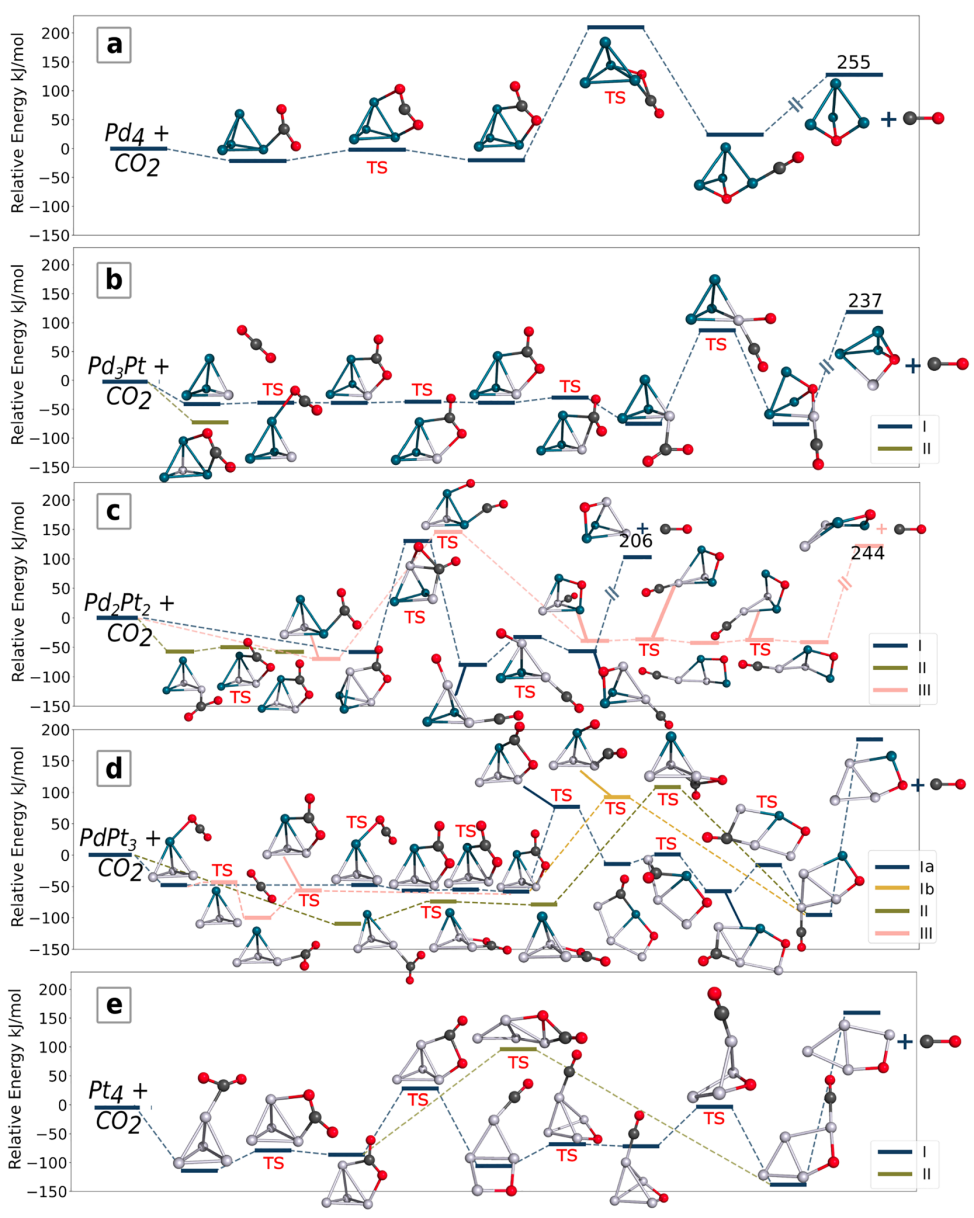
Computed reaction paths for CO_2_ activation and dissociation on Pd_*x*_Pt_4–*x*_ clusters. The CO detachment reaction line is broken for Pd_4_, Pd_3_Pt, and Pd_2_Pt_2_.Transition structures are marked with a red “TS”.

**Figure 7:**
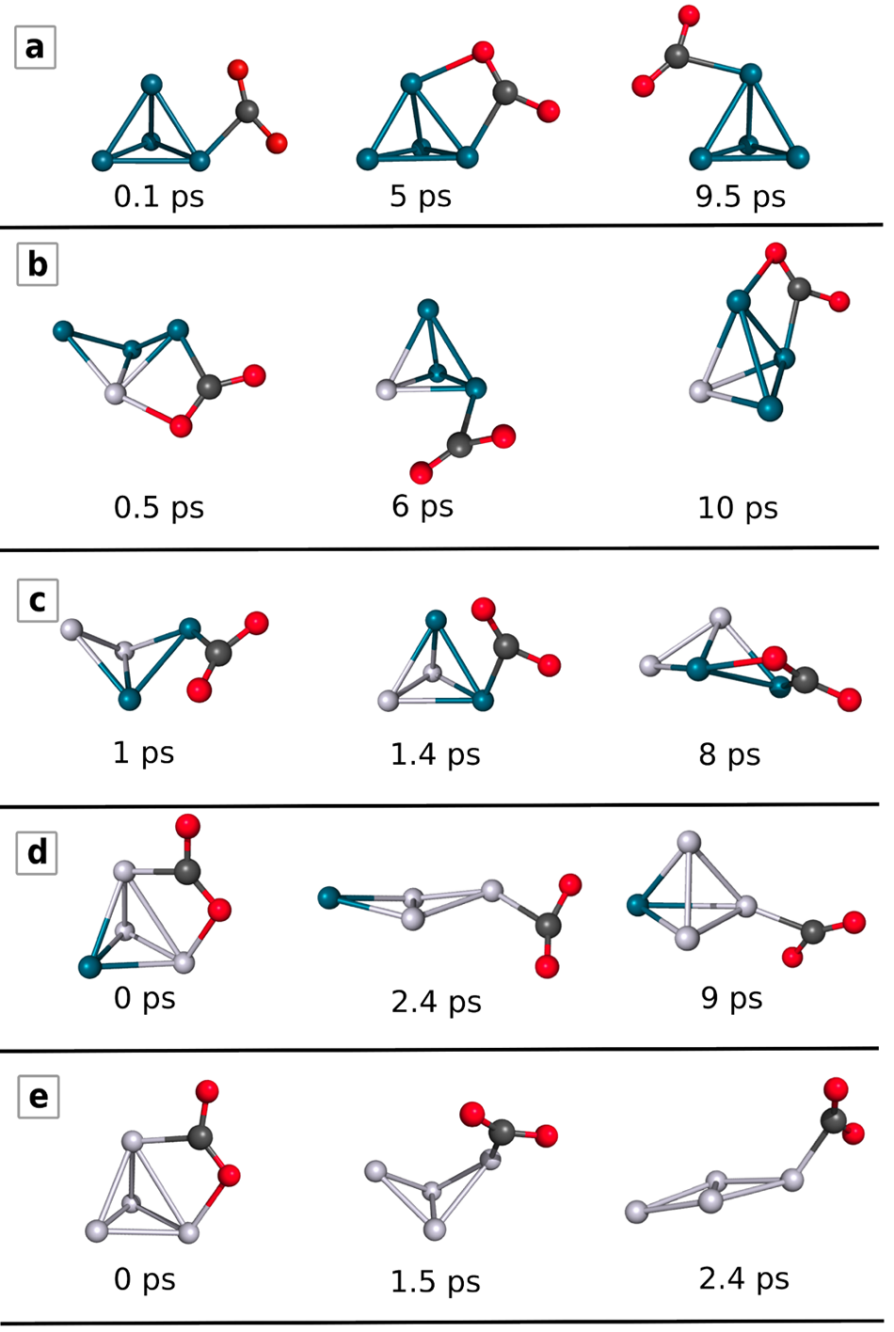
Selected snapshots from the AIMD simulations.

Interestingly, the computations showed several possible CO_2_ activation and dissociation reaction paths with highly different activation barriers (Figure 6). These results infer that CO_2_ dissociation on Pd_*x*_Pt_4–*x*_ clusters in the gas phase strongly depends on the binding site and binding mode. For bimetallic cluster compositions, the number of the possible reaction paths and their complexity both increase compared with the pure clusters. In the following, we refer to Figures 6 and 7 to describe and analyze in detail the several reaction paths and the AIMD simulations, respectively.

#### Pd_4_

Figure 6a shows the CO_2_ dissociation path on the Pd_4_ cluster. The CO_2_ activation and dissociation process takes place in three steps. In the first intermediate, the short Pd–C distance of 2.07 Å and the Pd–O distances of 2.29 and 2.8 Å indicate that the carbon and one of the oxygen atoms of the bent CO_2_ molecule are binding to the cluster, while in the second intermediate, the interatomic distances (Pd–C: 2.05 Å; shortest Pd–O distances: 2.43 and 2.37 Å) suggest that each atom of the carbon dioxide is interacting with palladium atoms. The energy barrier between these adducts is only 19 kJ/mol. The population of the CO_2_ π* orbital resulting from the electron donation during the adsorption (see the EDA section and Figure 5) weakens the C–O bond, clearly indicating its activation. Next, an energy barrier of 230 kJ/mol is needed for the C–O bond dissociation, where a triply coordinated oxygen atom (binding mode vi in Figure S17b in the SI) is formed and CO binds to one of the adjacent palladium atoms. The CO elimination requires a further 230 kJ/mol, indicating relatively strong binding. The Pd_4_ cluster does not undergo structural deformation during the CO_2_ dissociation process and keeps its tetrahedral configuration. DFT-based molecular dynamics simulations at 500 K further confirm that the Pd_4_ moiety maintains its rigid, tetrahedral structure in the adduct (see Figure 7a), while the CO_2_ molecule fluctuates around the metal atom that was the closest to carbon at the start of the simulation.

Overall, the large activation barrier suggests that the CO_2_ dissociation is hindered on the Pd_4_ cluster, while it may be deactivated by the bound CO molecule.

#### Pd_3_Pt

In this case, the CO_2_ molecule can bind either to a platinum–palladium edge (reaction path I) or to the one with two palladium atoms (reaction path II), with the latter being the energetically more favored (Figure 6b). However, from our systematic adducts screening, we did not identify any suitable candidate structure for further reaction from the more stable initial adduct with CO_2_ bound to two palladium atoms (the adjacent Pd–C distance is 2.04 Å, and Pd–O distances are 2.45 and 2.35 Å, respectively), showing the termination of the reaction here. This shows that the CO_2_ dissociation is hindered if the molecule is bound to a palladium atom, in agreement with the results for the Pd_4_ cluster.

For path I, it is very visible in Figure 6b that CO_2_ can migrate easily on the cluster. The reaction path is very flat, with energy barriers ranging from 5 to 25 kJ/mol. Eventually, the carbon will bind to the platinum atom, from where the carbon dioxide dissociation is possible with an energy barrier of 162 kJ/mol. It is interesting to note that substitution of a single palladium by platinum in Pd_4_ lowers the CO_2_ dissociation energy by approximately 50 kJ/mol, i.e., by about 25%. This shows that the introduction of a single platinum atom enhances both the binding and also the dissociation of the CO_2_ molecule. The transition metal cluster keeps its tetrahedron-like structure upon CO_2_ dissociation, as was observed for the Pd_4_ cluster. Detaching CO from the product structure needs high energy of 313 kJ/mol, nearly 100 kJ/mol more than that for Pd_4_.

During the molecular dynamics simulation of the Pd_3_Pt cluster (Figure 7b), we observe that the cluster opens its tetrahedral structure into a bent-rhomboidal shape after 0.5 ps, while the cluster closes again to tetrahedral geometry around 6 ps. This highlights the importance of the dynamical processes, as the static reaction paths did not show the emergence of bent-rhomboidal cluster shapes. Similarly to Pd_4_, the CO_2_ molecule fluctuates around the cluster surface.

#### Pd_2_Pt_2_

For the CO_2_ adducts of the Pd_2_Pt_2_ cluster (Figure 6c), the reaction can start in three directions as the CO_2_ molecule can bind on the platinum–platinum(I), platinum–palladium(II), or palladium–palladium edge (III).

Path I starts with a bidentate CO_2_ adduct. Subsequently, 188 kJ/mol energy is needed for the CO_2_ dissociation, which leads first to an interesting structure, with a formally monovalent, platinum-bound oxygen atom and where the CO molecule binds to the other platinum atom. A relatively small energy barrier leads to the structure with bridge position oxygen, from where the CO dissociation is possible. The cluster maintains the tetrahedral structure upon CO_2_ dissociation.

The intact, monodentate-bound CO_2_ entrance complex of reaction path II is low in energy, while a small reaction barrier leads to the somewhat less stable bidentate structure. This suggests dynamical behavior of the bound CO_2_ molecule. We did not find any subsequent dissociation reactions, which may be explained by the energetic stability of the entrance adduct.

Reaction path III starts with an intact, bidentate-bound CO_2_ adduct, which is followed by a high energy barrier (215 kJ/mol), corresponding to carbon–oxygen bond breaking. For the subsequent intermediates and transition structures, the reaction path is flat, and we observe energy differences of about 10 kJ/mol among the different structures. This reaction leads to a quadrangular, boat-shaped cluster with dissociated CO_2_; thus, in contrast to the Pd_4_ and Pd_3_Pt clusters, the tetrahedral shape of the metal cluster is not kept by the product. This is because, after CO_2_ dissociation, the CO binds to the platinum atom, and we know from EDA that the charge transfer between platinum and the carbon atom is greater than that for the palladium–carbon. This suggests that since the Pt–C charge transfer weakens the internal cluster structure, it consequently deforms to a bent-rhomboidal cluster.

In line, the molecular dynamics simulation of the Pd_2_Pt_2_ + CO_2_–cluster adduct structure shows an umbrella-like fluctuating motion (Figure 7c): at 1 ps, the cluster opens into a bent-rhomboidal structure; then it closes back to the tetrahedron shape at 1.3 ps and reopens again into the rhomboidal shape at 8 ps. The CO_2_ migrates around the palladium atoms, and we never observe CO_2_ dissociation, in agreement with the corresponding large energy barrier.

#### PdPt_3_

We found three reaction paths for the PdPt_3_ CO_2_ adducts (Figure 6d). In the first two reaction paths, the CO_2_ binds either to a palladium(I) or to a platinum atom (II), while in path III, it binds first to a palladium and then migrates to a platinum. Subsequently, path III merges to path I. The reaction paths lead to dissociated CO_2_ product, and the cluster adopts a bent-rhomboidal shape, with bridge oxygen between a platinum and a palladium atom, whereas the CO is bound to another platinum atom.

Reaction path I can diverge to paths Ia and Ib. These reactions start with an intermediate having a unique CO_2_ binding mode, found only in this case (denoted by type ii in Figure S16b in the SI). The subsequent barrier of about 10 kJ/mol leads to a bidentate adduct, with Pd–C and Pd–O distances of 1.95 and 2.13 Å, respectively. After, an activation energy of 150 kJ/mol is needed to dissociate CO_2_ and the structure is relaxed during several steps. Alternatively, CO_2_ dissociation and the subsequent relaxation of the product structure can proceed in a single reaction step (path Ib). This leads to the same structure as the multistep dissociation and relaxation reaction, but with a somewhat higher activation energy.

In both paths II and III, the carbon atom of the CO_2_ molecule is bound first to a platinum atom of the cluster, and they differ only in the orientation of CO_2_ with respect to the cluster. While these two reaction paths involve different CO_2_ dissociation steps, both lead to the same dissociated CO_2_ product.

In path II, the final product with dissociated CO can be reached by an energy barrier of 187 kJ/mol.

Path III involves CO_2_ bound to both Pd and Pt atoms. The CO_2_ molecule binds to one of the palladium atoms, then it rotates around the cluster and binds to the platinum atom, and finally it rotates again and binds to a palladium. The reaction steps involving palladium- and platinum-bound CO_2_ have small barriers.

The AIMD simulation of the PdPt_3_ cluster with CO_2_ shows that the cluster opens its structure into an almost planar rhomboidal one after 2.4 ps, and it recovers to the tetrahedral shape again at 9 ps (see Figure 7d). Again, we observe the shape fluctuation of the cluster but not CO_2_ dissociation. We observe cluster shape fluctuation and CO_2_ migration around the platinum atoms, regardless of the atoms to which the CO_2_ is binding in the initial structure of the simulations.

#### Pt_4_

Figure 6e shows two CO_2_ dissociation pathways (denoted by I and II, respectively) on the Pt_4_ cluster. The final product is a quadrangular structure with dissociated CO_2_. Reaction path I involves several steps, but the highest-energy barrier we observed is about 115 kJ/mol, and the structures with dissociated CO_2_ are energetically favored.

Path II involves fewer intermediates than path I and reaches the final product structure in one reaction step in which the metal cluster loses its tetrahedron shape and opens into a metastable quadrangular structure. However, the energy barrier to overcome is of about 200 kJ/mol; thus, the multistep reaction path I is more feasible. The reaction paths in Figure 6e show two stable structures in which the Pt_4_ cluster distorts from its tetrahedral shape. This is in line with the low-energy quadrangular geometry of the bare cluster; see Figure 1. The required energy to detach the CO from the lowest-lying energy adduct is 294 kJ/mol. These results support the best-known problem of CO poisoning of platinum.^96^

The tetrahedral Pt_4_ part of the cluster–CO_2_ adduct starts opening after 1.5 ps in the AIMD simulation (Figure 7e). At around 3 ps, its structure is planar, and the cluster keeps its rhomboidal structure for the rest of the simulation time. The fact that the cluster structure holds the planar configuration confirms our observations of stable planar structures in both the bare cluster and the reaction path computations. It is interesting to note that the fluxionality of the graphene-supported Pt_4_ cluster was observed experimentally.^97^

### CO_2_ Activation and Dissociation and CO Detachment

Our computations show that both CO_2_ activation and dissociation on Pd_*x*_Pt_4–*x*_ clusters involve multistep reaction paths. The reactivity can be tuned by the cluster composition. The CO_2_ binding to the Pd_*x*_Pt_4–*x*_ cluster is an exothermic process; the adsorption energy increases proportionally with the platinum doping, from at least −20 kJ/mol (CO_2_ + Pd_4_) and at most −114 kJ/mol (CO_2_ + Pt_4_). The carbon atom binds before the oxygen to the cluster, and both mono- and bidentate binding modes correspond to minima on the potential energy surface. The stability of the activated structures indicates that CO_2_ activation on the cluster is a favored process. The EDA shows that charge transfer plays an important role in the CO_2_ activation.

In Figure 9, we compare the natural charges of the activated CO_2_ in the cluster adducts, of the detached oxygen in the dissociated CO_2_ adducts, and of the oxygen atom in the oxides. With the increased platinum content of the clusters, the cluster-bound CO_2_ molecule shows more negative charge, in line with the scaling of the EDA charge transfer energy with the platinum content. This is explained by the higher CO_2_ binding affinity of platinum than that of palladium. From activation to dissociation, the anionic character of the cluster-bound oxygen atom (O*) increases with the platinum content. In the dissociated CO_2_ adducts (which contain O* and CO* moieties), the charge of the oxygen does not correlate with the cluster composition. The charge of the O* in the dissociated CO_2_ structure can depend on different factors, such as the energy transfer of the platinum atoms to the CO, the cluster shape, the M–CO (M denotes the nearest Pd or Pt atom) distance,^98^ or the adjacency of O* and CO* in the adduct. In the oxides, the natural charge of the oxygen decreases with increasing platinum doping.

**Figure 8:**
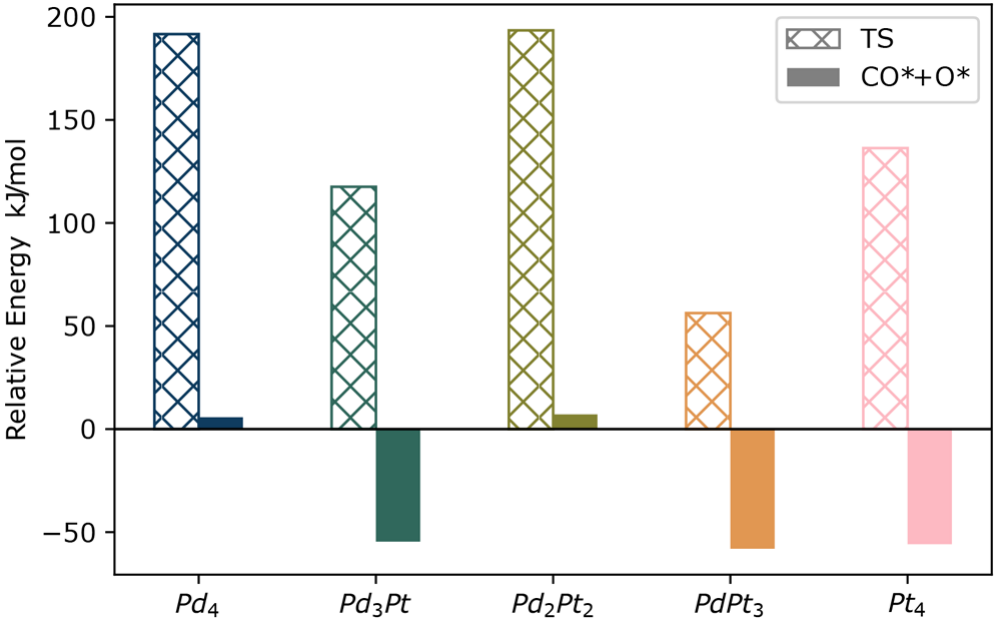
Relative energies of the highest-energy transition states (TS) and the lowest-energy adducts for which we observe dissociated CO_2_, compared to the separated cluster and CO_2_. Level of theory: TPSSh+D3/def2-QZVPPD//PBE+D3/def2-TZVP.

**Figure 9:**
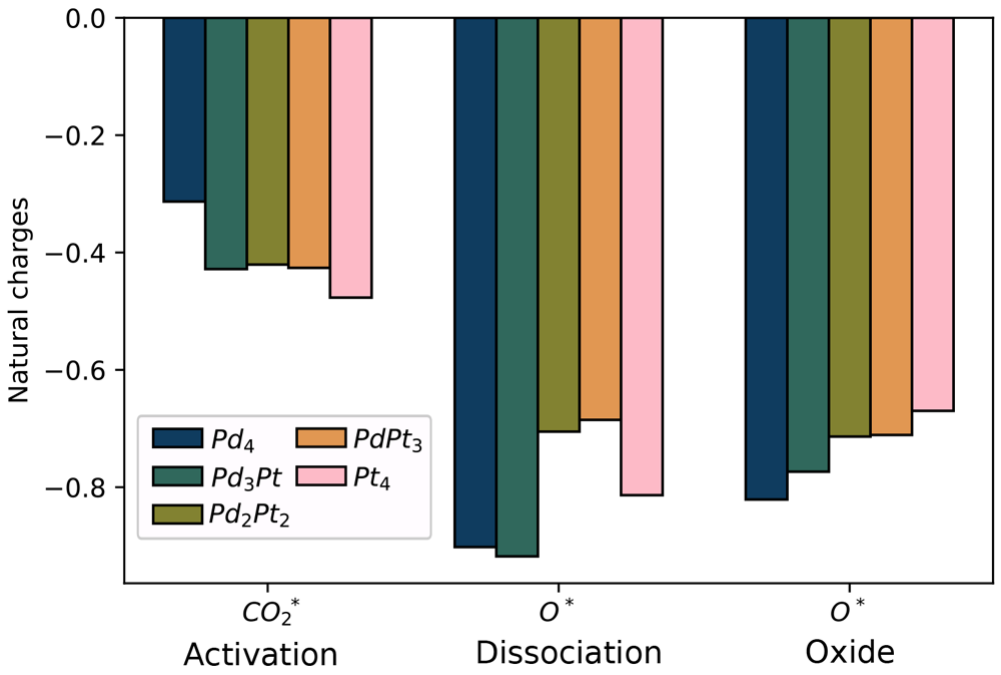
Natural charges of the CO_2_ in the activated CO_2_–cluster adducts, of the dissociated oxygen in the dissociated CO_2_ adducts, and of the oxygen atom in the oxides.

The AIMD simulations show CO_2_ activation but not dissociation within the 10 ps simulation time. The CO_2_ binding weakens the cluster structure and makes it more fluxional. In most cases, the shape fluctuation is clearly shown only by the molecular dynamics simulations and not by the static reaction paths. Low energy barriers correspond also to the intact CO_2_ migration on the cluster surface, which implies a lower probability of a system to localize enough nuclear kinetic energy toward the CO_2_ dissociation, thus decreasing the CO_2_ dissociation rate. This is because multiple processes (cluster structure relaxation, fluctuations, etc.) concur with dissociation, even when starting from all of the intact CO_2_ structures on the reaction path. This highlights the role of the entropy effects beyond the conventionally applied rigid rotor harmonic oscillator approximation, and, as has been shown earlier, an ensemble of structures must be taken into account to compute thermodynamic and kinetic quantities.^99^ In the Pd_*x*_Pt_4–*x*_ adducts, the structure fluxionality and the CO_2_ activation can proceed simultaneously. We report additional analysis of the trajectories as well as trajectories videos in the SI, in the section Further AIMD Simulations Analysis, and as separate folder, respectively.

The heights of the CO_2_ dissociation energy barriers on the different reaction paths depend not only on the cluster composition but also on the binding site and on the geometry of the dissociated product. For instance, in the case of the Pt_4_ cluster, we located two different dissociation rate-limiting transition structures, from which the one corresponding to a multistep reaction is the lower by 33 kJ/mol.

For the reactants, the highest-energy transition structures, and the lowest-energy adducts with dissociated CO_2_, we computed the single-point energies on the PBE+D3/def2-TZVP geometries using the expectedly more accurate TPSSh+D3/def2-QZVPP method (Figure 8). Based on their energies, the dissociated CO_2_ products can be divided into two groups as dissociation is endothermic for Pd_4_ and Pd_2_Pt_2_ and exothermic for Pd_3_Pt, PdPt_3_, and Pt_4_. The CO_2_ dissociation energy barriers decrease in the order of Pd_2_Pt_2_ > Pd_4_ > Pt_4_ > Pd_3_Pt > PdPt_3_, and that of Pt_4_ is the median value. However, Pt_4_ isomerizes quickly to its rhomboidal shape (see the Reaction Paths section), and Pt_4_, like pure platinum, is prone to CO poisoning.^12^ Further information can be found in the section Direct CO_2_ Dissociation from the Reaction Paths in the SI.

The Pd_3_Pt cluster composition is of particular interest as the intactly bound CO_2_ is already activated and the CO_2_ dissociation barrier is still relatively high (162 kJ/mol). In general, the CO_2_ migration has small barriers on this cluster (see Figure 6b). It is interesting to note that in this cluster the palladium to platinum ratio (75% Pd, 25% Pt) is similar to that in Pd_70_Pt_30_, which, according to the experimental study of Kortlever et al.,^17^ yields a maximum faradaic efficiency in the CO_2_ electroreduction to formate. Moreover, experimental studies for the synthesis of half-shell Pd_3_Pt nanocrystals (25–40 nm, 5 nm thickness) report that the material is a good electrocatalyst because it is rich of active sites and not prone to CO poisoning.^100^

## Conclusions

We investigated the role of the Pd_*x*_Pt_4–*x*_ cluster composition for CO_2_ activation and dissociation in the gas phase, analyzing the detailed reaction mechanisms of these processes with respect to the cluster composition. The systematic screening of different initial cluster geometries with singlet, triplet, or quintet spin states pointed out that the triplet, tetrahedron-shaped clusters are the most stable in the gas phase. For the case of the Pt_4_, benchmark computations confirmed the stability of the tetrahedral triplet state compared to the close-lying quadrangular quintet.

We generated systematically the CO_2_ adducts for the most stable structures, for each cluster composition, considering both intact and dissociated CO_2_ binding modes to the cluster. For the most stable adducts, we computed the MBOs and performed ALMO-EDA. Especially the ALMO-EDA showed two main results for the intact CO_2_ adducts: first, the cluster charge transfer energy is linearly proportional to the interaction energy; second, the charge transfer is not scaling with the cluster composition. The bent geometry of the cluster-bound CO_2_, its negative natural charges, and the EDA clearly show activated CO_2_ molecule in the intactly bound adducts.

With the most stable structures, we located multiple reaction paths, starting on the different binding sites on the cluster. These reaction paths show CO_2_ activation, dissociation, and finally CO detachment from the cluster. We observed multistep reaction paths in all cases. CO_2_ adsorption is a favored process, with the carbon atom being adsorbed first to the cluster. The CO_2_ dissociation barrier depends on the CO_2_ binding site to the cluster; in general, increasing platinum content tends to lower the energy barrier or to create the possibility of multistep isomerizations of the adducts that lower the dissociation energy barrier as well. The CO detachment barrier is higher if it is bound to a platinum atom, confirming the well-known issue of CO poisoning for platinum catalysts.

Considering the reaction paths starting from several possible initial configurations led to a large number of optimized structures, among which we selected the most feasible configurations for CO_2_ activation and dissociation. The order for CO_2_ dissociation our computations show is, in decreasing order of the highest-energy transition structures, Pd_2_Pt_2_ > Pd_4_ > Pt_4_ > Pd_3_Pt > PdPt_3_, different from the one reported for the india-supported clusters.^29^ Moreover, our results are relevant for comparison to experiments in the gas phase, whose techniques strongly contribute in the development of functional materials.^32^ Our understanding of the processes depends purely on the electronic configuration due to the cluster’s chemical composition.

The understanding of the reactivities was then further augmented using AIMD simulations. The molecular dynamics computations clearly showed that the gas-phase cluster backbones are fluxional in several cases, similarly to the experimentally observed fluxionality of the graphene-supported Pt_4_ cluster.^97^ Importantly, our AIMD simulations showed that CO_2_ can fluctuate freely around its closest atoms. This emphasizes the role of the entropy, which (giving that the product CO is bound more strongly to the cluster) may lead to less favored CO_2_ dissociation. This highlights the role of the detailed reaction paths in understanding the platinum–palladium cluster reactivity and also in the catalyst design for CO_2_ activation.

In this study, we investigated small gas-phase model clusters, but such multistep reactions and the dynamical CO_2_ migration pathways are expected to take place on supported clusters as well. Thus, the effect of the cluster size, solvent, and support will be the subject of future research.

## Supporting Information Available

Test of spin multiplicity and basis set benchmarking of bare Pd_*x*_Pt_4–*x*_ clusters; benchmarking of Pt_4_ geometry and spin multiplicity; testing the accuracy of the PBE-D3/def2-TZVP method compared to coupled cluster computations; testing of spin multiplicity and CO_2_ binding modes; Mayer bond order analysis; detailed EDA; direct CO_2_ dissociation from the reaction paths; further AIMD simulations analysis; reduction of the oxide clusters; coordinates of the structures in the reaction paths (PDF)
Video of Pd^4^ (MP4)
Video of Pd^3^Pt (MP4)
Video of Pd^2^Pt^2^ (MP4)
Video of PdPt^3^ (MP4)
Video of Pt^4^(MP4)

## References

[ref1] Kahn M. A.; Khan M. Z.; Zaman K.; Naz L. Global Estimates of Energy Consumption and Greenhouse Gas Emissions. Renewable and Sustainable Energy Reviews 2014, 29, 336–344. 10.1016/j.rser.2013.08.091.

[ref2] Chu S.; Majumdar A. Opportunities and Challenges for a Sustainable Energy Future. Nature 2012, 488, 294–303. 10.1038/nature11475. 22895334

[ref3] Liu Z.; et al. Near-Real-Time Monitoring of Global CO2 Emissions Reveals the Effects of the COVID-19 Pandemic. Nat. Commun. 2020, 11, 5172 10.1038/s41467-020-20254-5. 33057164 PMC7560733

[ref4] Liu Z.; Deng Z.; Davis S. J.; Giron C.; Ciais P. Monitoring Global Carbon Emissions in 2021. Nature Reviews Earth & Environment 2022, 3, 217–219. 10.1038/s43017-022-00285-w. PMC8935618 35340723

[ref5] Nejat P.; Jomehzadeh F.; Taheri M. M.; Gohari M.; Abd. Majid M. Z. A Global Review of Energy Consumption, CO2 Emissions and Policy in the Residential Sector (with an Overview of the Top Ten CO2 Emitting Countries). Renewable and Sustainable Energy Reviews 2015, 43, 843–862. 10.1016/j.rser.2014.11.066.

[ref6] Boutin E.; Merakeb L.; Ma B.; Boudy B.; Wang M.; Bonin J.; Anxolabéhère-Mallart E.; Robert M. Molecular Catalysis of CO2 Reduction: Recent Advances and Perspectives in Electrochemical and Light-Driven Processes with Selected Fe, Ni and Co aza Macrocyclic and Polypyridine Complexes. Chem. Soc. Rev. 2020, 49, 5772–5809. 10.1039/D0CS00218F. 32697210

[ref7] Fais B.; Sabio N.; Strachan N. The Critical Role of the Industrial Sector in Reaching Long-Term Emission Reduction, Energy Efficiency and Renewable Targets. Applied Energy 2016, 162, 699–712. 10.1016/j.apenergy.2015.10.112.

[ref8] Whipple D. T.; Kenis P. J. A. Prospects of CO2 Utilization via Direct Heterogeneous Electrochemical Reduction. J. Phys. Chem. Lett. 2010, 1, 3451–3458. 10.1021/jz1012627.

[ref9] Min X.; Kanan M. W. Pd-Catalyzed Electrohydrogenation of Carbon Dioxide to Formate: High Mass Activity at Low Overpotential and Identification of the Deactivation Pathway. J. Am. Chem. Soc. 2015, 137, 4701–4708. 10.1021/ja511890h. 25812119

[ref10] Seh Z. W.; Kibsgaard J.; Dickens C. F.; Chorkendorff I.; Nørskov J. K.; Jaramillo T. F. Combining Theory and Experiment in Electrocatalysis: Insights Into Materials Design. Science 2017, 355, eaad4998 10.1126/science.aad4998. 28082532

[ref11] Lee J. H.; Kattel S.; Jiang Z.; Xie Z.; Yao S.; Tackett B. M.; Xu W.; Marinkovic N. S.; Chen J. G. Tuning the Activity and Selectivity of Electroreduction of CO2 to Synthesis Gas Using Bimetallic Catalysts. Nat. Commun. 2019, 10, 3724 10.1038/s41467-019-11352-0. 31427576 PMC6700200

[ref12] Jung N.; Cho Y.-H.; Ahn M.; Lim J. W.; Kang Y. S.; Chung D. Y.; Kim J.; Cho Y.-H.; Sung Y.-E. Methanol-Tolerant Cathode Electrode Structure Composed of Heterogeneous Composites to Overcome Methanol Crossover Effects for Direct Methanol Fuel Cell. Int. J. Hydrogen Energy 2011, 36, 15731–15738. 10.1016/j.ijhydene.2011.09.054.

[ref13] Diercks J. S.; Georgi M.; Herranz J.; Diklić N.; Chauhan P.; Clark A. H.; Hübner R.; Faisnel A.; Chen Q.; Nachtegaal M.; Eychmüller A.; Schmidt T. J. CO2 Electroreduction on Unsupported PdPt Aerogels: Effects of Alloying and Surface Composition on Product Selectivity. ACS Applied Energy Materials 2022, 5, 8460–8471. 10.1021/acsaem.2c00987.

[ref14] Valenti M.; Prasad N. P.; Kas R.; Bohra D.; Ma M.; Balasubramanian V.; Chu L.; Gimenez S.; Bisquert J.; Dam B.; Smith W. A. Suppressing H2 Evolution and Promoting Selective CO2 Electroreduction to CO at Low Overpotentials by Alloying Au with Pd. ACS Catal. 2019, 9, 3527–3536. 10.1021/acscatal.8b04604.

[ref15] Chen X.; Granda-Marulanda L. P.; McCrum I. T.; Koper M. How Palladium Inhibits CO Poisoning During Electrocatalytic Formic Acid Oxidation and Carbon Dioxide Reduction. Nat. Commun. 2022, 13, 38 10.1038/s41467-021-27793-5. 35013444 PMC8748733

[ref16] Zhang H.-X.; Wang C.; Wang J.-Y.; Zhai J.-J.; Cai W.-B. Carbon-Supported Pd-Pt Nanoalloy with Low Pt Content and Superior Catalysis for Formic Acid Electro-oxidation. J. Phys. Chem. C 2010, 114, 6446–6451. 10.1021/jp100835b.

[ref17] Kortlever R.; Peters I.; Koper S.; Koper M. T. Electrochemical CO2 Reduction to Formic Acid at Low Overpotential and with High Faradaic Efficiency on Carbon-Supported Bimetallic Pd–Pt Nanoparticles. ACS Catal. 2015, 5, 3916–3923. 10.1021/acscatal.5b00602.

[ref18] Alvarez-Garcia A.; Flórez E.; Moreno A.; Jimenez-Orozco C. CO2 Activation on Small Cu-Ni and Cu-Pd Bimetallic Clusters. Molecular Catalysis 2020, 484, 110733 10.1016/j.mcat.2019.110733.

[ref19] Klaja O.; Szczygieł J.; Trawczyński J.; Szyja B. M. The CO2 Dissociation Mechanism on the Small Copper Clusters—the Influence of Geometry. Theor. Chem. Acc. 2017, 136, 1–9. 10.1007/s00214-017-2129-4.

[ref20] Green A. E.; Justen J.; Schöllkopf W.; Gentleman A. S.; Fielicke A.; Mackenzie S. R. IR Signature of Size-Selective CO2 Activation on Small Platinum Cluster Anions, Ptn- (n = 4–7). Angew. Chem., Int. Ed. 2018, 57, 14822–14826. 10.1002/anie.201809099. 30207020

[ref21] Barwa E.; Pascher T. F.; Ončák M.; van der Linde C.; Beyer M. K. Carbon Dioxide Activation at Metal Centers: Evolution of Charge Transfer from Mg.+ to CO2 in [MgCO2(H2O)n].+, n = 0–8. Angew. Chem., Int. Ed. 2020, 59, 7467–7471. 10.1002/anie.202001292. PMC7217156 32100953

[ref22] Bernhardt T. M. Gas-phase Kinetics and Catalytic Reactions of Small Silver and Gold Clusters. Int. J. Mass Spectrom. 2005, 243, 1–29. 10.1016/j.ijms.2004.12.015.

[ref23] Ren M.; Zhang Y.; Wang X.; Qiu H. Catalytic Hydrogenation of CO2 to Methanol: A Review. Catalysts 2022, 12, 403 10.3390/catal12040403.

[ref24] Yan G.; Gao Z.; Zhao M.; Yang W.; Ding X. CO2 Hydrogenation to Formic Acid over Platinum Cluster Doped Defective Graphene: A DFT Study. Appl. Surf. Sci. 2020, 517, 146200 10.1016/j.apsusc.2020.146200.

[ref25] Yang S.; Rao D.; Ye J.; Yang S.; Zhang C.; Gao C.; Zhou X.; Yang H.; Yan X. Mechanism of Transition Metal Cluster Catalysts for Hydrogen Evolution Reaction. Int. J. Hydrogen Energy 2021, 46, 3484–3492. 10.1016/j.ijhydene.2020.11.008.

[ref26] Galvez-Gonzalez L. E.; Juarez-Sanchez J. O.; Pacheco-Contreras R.; Garzon I. L.; Paz-Borbon L. O.; Posada-Amarillas A. CO2 Adsorption on Gas-Phase Cu4-xPtx (x = 0–4) Clusters: a DFT Study. Phys. Chem. Chem. Phys. 2018, 20, 17071–17080. 10.1039/C8CP00818C. 29896596

[ref27] Niu J.; Ran J.; Ou Z.; Du X.; Wang R.; Qi W.; Zhang P. CO2 Dissociation over PtxNi4-x Bimetallic Clusters with and without Hydrogen Sources: A Density Functional Theory Study. Journal of CO2 Utilization 2016, 16, 431–441. 10.1016/j.jcou.2016.10.008.

[ref28] Liu C.; He H.; Zapol P.; Curtiss L. A. Computational studies of electrochemical CO2 reduction on subnanometer transition metal clusters. Phys. Chem. Chem. Phys. 2014, 16, 26584–26599. 10.1039/C4CP02690J. 25158148

[ref29] Wang X.; Pan J.; Wei H.; Li W.; Zhao J.; Hu Z. CO2 Activation and Dissociation on In2O3(110) Supported PdnPt(4-n) (n = 0–4) Catalysts: a Density Functional Theory Study. Phys. Chem. Chem. Phys. 2021, 23, 11557–11567. 10.1039/D1CP01015H. 33978017

[ref30] Ha M.-A.; Dadras J.; Alexandrova A. Rutile-Deposited Pt–Pd clusters: A Hypothesis Regarding the Stability at 50/50 Ratio. ACS Catal. 2014, 4, 3570–3580. 10.1021/cs5011426.

[ref31] Lang S. M.; Bernhardt T. M. Gas Phase Metal Cluster Model Systems for Heterogeneous Catalysis. Phys. Chem. Chem. Phys. 2012, 14, 9255–9269. 10.1039/c2cp40660h. 22669249

[ref32] Luo Z.; Castleman A. W. J.; Khanna S. N. Reactivity of Metal Clusters. Chem. Rev. 2016, 116, 14456–14492. 10.1021/acs.chemrev.6b00230. 27960263

[ref33] Lang S. M.; Fleischer I.; Bernhardt T. M.; Barnett R. N.; Landman U. Low-Temperature CO Oxidation Catalyzed by Free Palladium Clusters: Similarities and Differences to Pd Surfaces and Supported Particles. ACS Catal. 2015, 5, 2275–2289. 10.1021/cs5016222.

[ref34] Ugartemendia A.; Peeters K.; Ferrari P.; de Cozar A.; Mercero J. M.; Janssens E.; Jimenez-Izal E. Doping Platinum with Germanium: An Effective Way to Mitigate the CO Poisoning. ChemPhysChem 2021, 22, 1603–1610. 10.1002/cphc.202100407. 34058042

[ref35] Fu C.; Liu C.; Li T.; Zhang X.; Wang F.; Yang J.; Jiang Y.; Cui P.; Li H. DFT Calculations: A Powerful Tool for Better Understanding of Electrocatalytic Oxygen Reduction Reactions on Pt-based Metallic Catalysts. Comput. Mater. Sci. 2019, 170, 109202 10.1016/j.commatsci.2019.109202.

[ref36] Liao X.; Lu R.; Xia L.; Liu Q.; Wang H.; Zhao K.; Wang Z.; Zhao Y. Density Functional Theory for Electrocatalysis. ENERGY & ENVIRONMENTAL MATERIALS 2022, 5, 157–185. 10.1002/eem2.12204.

[ref37] Batchelor T. A.; Pedersen J. K.; Winther S. H.; Castelli I. E.; Jacobsen K. W.; Rossmeisl J. High-Entropy Alloys as a Discovery Platform for Electrocatalysis. Joule 2019, 3, 834–845. 10.1016/j.joule.2018.12.015.

[ref38] Wang L.; Zhang W.; Wang S.; Gao Z.; Luo Z.; Wang X.; Zeng R.; Li A.; Li H.; Wang M.; et al. Atomic-Level Insights in Optimizing Reaction Paths for Hydroformylation Reaction over Rh/CoO Single-Atom Catalyst. Nat. Commun. 2016, 7, 1–8. 10.1038/ncomms14036. PMC5196038 28004661

[ref39] Mattioli G.; Giannozzi P.; Amore Bonapasta A.; Guidoni L. Reaction Pathways for Oxygen Evolution Promoted by Cobalt Catalyst. J. Am. Chem. Soc. 2013, 135, 15353–15363. 10.1021/ja401797v. 24044778 PMC3912752

[ref40] Hossain M. D.; Huang Y.; Yu T. H.; Goddard W. A. III; Luo Z. Reaction Mechanism and Kinetics for CO2 Reduction on Nickel Single Atom Catalysts from Quantum Mechanics. Nat. Commun. 2020, 11, 1–14. 10.1038/s41467-020-16119-6. 32382033 PMC7205999

[ref41] Kortlever R.; Shen J.; Schouten K. J. P.; Calle-Vallejo F.; Koper M. T. M. Catalysts and Reaction Pathways for the Electrochemical Reduction of Carbon Dioxide. J. Phys. Chem. Lett. 2015, 6, 4073–4082. 10.1021/acs.jpclett.5b01559. 26722779

[ref42] Zhang X.; Liu J.-X.; Zijlstra B.; Filot I. A.; Zhou Z.; Sun S.; Hensen E. J. Optimum Cu Nanoparticle Catalysts for CO2 Hydrogenation towards Methanol. Nano Energy 2018, 43, 200–209. 10.1016/j.nanoen.2017.11.021.

[ref43] Shi C.; O’Grady C. P.; Peterson A. A.; Hansen H. A.; Nørskov J. K. Modeling CO2 Reduction on Pt(111). Phys. Chem. Chem. Phys. 2013, 15, 7114–7122. 10.1039/c3cp50645b. 23552398

[ref44] Ye J.; Liu C.-j.; Mei D.; Ge Q. Methanol Synthesis from CO2 Hydrogenation over a Pd4/In2O3Model Catalyst: A Combined DFT and Kinetic Study. J. Catal. 2014, 317, 44–53. 10.1016/j.jcat.2014.06.002.

[ref45] Wang X.; Pan J.; Wei H.; Li W.; Zhao J.; Hu Z. Mechanism of Methanol Synthesis from CO2 Hydrogenation over Pt8/In2O3 Catalysts: A Combined Study on Density Functional Theory and Microkinetic Modeling. J. Phys. Chem. C 2022, 126, 1761–1769. 10.1021/acs.jpcc.1c08098.

[ref46] Sarkar S.; Peter S. C. C. Catalyst Designing Strategies for Electrochemical CO2 Reduction: A Perspective. Progress in Energy 2022, 4, 032002 10.1088/2516-1083/ac6e23.

[ref47] Ross M. B.; De Luna P.; Li Y.; Dinh C.-T.; Kim D.; Yang P.; Sargent E. H. Designing Materials for Electrochemical Carbon Dioxide Recycling. Nature Catalysis 2019, 2, 648–658. 10.1038/s41929-019-0306-7.

[ref48] Liu X.; Sun L.; Deng W.-Q. Theoretical Investigation of CO2 Adsorption and Dissociation on Low Index Surfaces of Transition Metals. J. Phys. Chem. C 2018, 122, 8306–8314. 10.1021/acs.jpcc.7b12660.

[ref49] Wang X.; Shi H.; Kwak J. H.; Szanyi J. Mechanism of CO2 Hydrogenation on Pd/Al2O3 Catalysts: Kinetics and Transient DRIFTS-MS Studies. ACS Catal. 2015, 5, 6337–6349. 10.1021/acscatal.5b01464.

[ref50] Perdew J. P.; Burke K.; Ernzerhof M. Generalized Gradient Approximation Made Simple. Phys. Rev. Lett. 1996, 77, 3865–3868. 10.1103/PhysRevLett.77.3865. 10062328

[ref51] Grimme S.; Ehrlich S.; Goerigk L. Effect of the Damping Function in Dispersion Corrected Density Functional Theory. J. Comput. Chem. 2011, 32, 1456–1465. 10.1002/jcc.21759. 21370243

[ref52] Patra A.; Bates J. E.; Sun J.; Perdew J. P. Properties of Real Metallic Surfaces: Effects of Density Functional Semilocality and van Der Waals Nonlocality. Proc. Natl. Acad. Sci. U. S. A. 2017, 114, E9188–E9196. 10.1073/pnas.1713320114. 29042509 PMC5676929

[ref53] Da Silva J. L.; Stampfl C.; Scheffler M. Converged Properties of Clean Metal Surfaces by All-Electron First-Principles Calculations. Surf. Sci. 2006, 600, 703–715. 10.1016/j.susc.2005.12.008.

[ref54] Cheng D.; Wang W. Tailoring of Pd–Pt Bimetallic Clusters with High Stability for Oxygen Reduction Reaction. Nanoscale 2012, 4, 2408–2415. 10.1039/c2nr12097f. 22374435

[ref55] Mendes P. C. D.; Verga L. G.; Da Silva J. L. F. Ab Initio Screening of Pt-Based Transition-Metal Nanoalloys Using Descriptors Derived from the Adsorption and Activation of CO2. Phys. Chem. Chem. Phys. 2021, 23, 6029–6041. 10.1039/D1CP00570G. 33683269

[ref56] Jin N.; Han J.; Wang H.; Zhu X.; Ge Q. A DFT Study of Oxygen Reduction Reaction Mechanism Over O-Doped Graphene-Supported Pt4, Pt3Fe and Pt3V Alloy Catalysts. Int. J. Hydrogen Energy 2015, 40, 5126–5134. 10.1016/j.ijhydene.2015.02.101.

[ref57] Koverga A. A.; Flórez E.; Jimenez-Orozco C.; Rodriguez J. A. Not All Platinum Surfaces Are the Same: Effect of the Support on Fundamental Properties of Platinum Adlayer and its Implications for the Activity toward Hydrogen Evolution Reaction. Electrochim. Acta 2021, 368, 137598 10.1016/j.electacta.2020.137598.

[ref58] Kowalec I.; Kabalan L.; Catlow C. R. A.; Logsdail A. J. A Computational Study of Direct CO2 Hydrogenation to Methanol on Pd Surfaces. Phys. Chem. Chem. Phys. 2022, 24, 9360–9373. 10.1039/D2CP01019D. 35383806

[ref59] Hay P. J.; Wadt W. R. Ab Initio Effective Core Potentials for Molecular Calculations. Potentials for K to Au Including the Outermost Core Orbitals. J. Chem. Phys. 1985, 82, 299–310. 10.1063/1.448975.

[ref60] Weigend F.; Ahlrichs R. Balanced Basis Sets of Split Valence, Triple Zeta Valence and Quadruple Zeta Valence Quality for H to Rn: Design and Assessment of Accuracy. Phys. Chem. Chem. Phys. 2005, 7, 3297–3305. 10.1039/b508541a. 16240044

[ref61] Davidson E. R. The Iterative Calculation of a Few of the Lowest Eigenvalues and Corresponding Eigenvectors of Large Real-Symmetric Matrices. J. Comput. Phys. 1975, 17, 87–94. 10.1016/0021-9991(75)90065-0.

[ref62] Behn A.; Zimmerman P. M.; Bell A. T.; Head-Gordon M. Efficient Exploration of Reaction Paths via a Freezing String Method. J. Chem. Phys. 2011, 135, 224108 10.1063/1.3664901. 22168681

[ref63] Cerjan C. J.; Miller W. H. On Finding Transition States. J. Chem. Phys. 1981, 75, 2800–2806. 10.1063/1.442352.

[ref64] Simons J.; Joergensen P.; Taylor H.; Ozment J. Walking on Potential Energy Surfaces. J. Phys. Chem. 1983, 87, 2745–2753. 10.1021/j100238a013.

[ref65] Enkovaara J.; et al. Electronic structure calculations with GPAW: a real-space implementation of the projector augmented-wave method. J. Phys.: Condens. Matter 2010, 22, 253202 10.1088/0953-8984/22/25/253202. 21393795

[ref66] Larsen A. H.; et al. The Atomic Simulation Environment-a Python Library for Working with Atoms. Journal of physics. Condensed matter: an Institute of Physics journal 2017, 29, 273002 10.1088/1361-648X/aa680e. 28323250

[ref67] Vanden-Eijnden E.; Ciccotti G. Second-Order Integrators for Langevin Equations with Holonomic Constraints. Chemical physics letters 2006, 429, 310–316. 10.1016/j.cplett.2006.07.086.

[ref68] Korpelin V.; Kiljunen T.; Melander M. M.; Caro M. A.; Kristoffersen H. H.; Mammen N.; Apaja V.; Honkala K. Addressing Dynamics at Catalytic Heterogeneous Interfaces with DFT-MD: Anomalous Temperature Distributions from Commonly Used Thermostats. J. Phys. Chem. Lett. 2022, 13, 2644–2652. 10.1021/acs.jpclett.2c00230. 35297635 PMC8959310

[ref69] Epifanovsky E.; et al. Software for the Frontiers of Quantum Chemistry: An Overview of Developments in the Q-Chem 5 Package. J. Chem. Phys. 2021, 155, 084801 10.1063/5.0055522. 34470363 PMC9984241

[ref70] Lu T.; Chen F. Multiwfn: A Multifunctional Wavefunction Analyzer. J. Comput. Chem. 2012, 33, 580–592. 10.1002/jcc.22885. 22162017

[ref71] Schrödinger L. L. C. PyMOL Molecular Graphics System, version 1.8; 2015.

[ref72] Crameri F. Scientific Colour Maps, 2021. Zenodo. https://doi.org/10.5281/zenodo.5501399 (accessed Nov 4, 2023). The development of the Scientific colour maps is not funded any longer, but will continue as a pro bono project for the scientific community.

[ref73] Jia T.-T.; Lu C.-H.; Ding K.-N.; Zhang Y.-F.; Chen W.-K. Oxidation of Pdn (n = 1–5) Clusters on Single Vacancy Graphene: A First-Principles Study. Computational and Theoretical Chemistry 2013, 1020, 91–99. 10.1016/j.comptc.2013.07.029.

[ref74] Lian X.; Guo W.; Liu F.; Yang Y.; Xiao P.; Zhang Y.; Tian W. DFT Studies on Pt3M (M = Pt, Ni, Mo, Ru, Pd, Rh) Clusters for CO Cxidation. Comput. Mater. Sci. 2015, 96, 237–245. 10.1016/j.commatsci.2014.09.025.

[ref75] Kua J.; Goddard W. A. Chemisorption of Organics on Platinum. 2. Chemisorption of C2Hx and CHx on Pt(111). J. Phys. Chem. B 1998, 102, 9492–9500. 10.1021/jp982527s.

[ref76] Parreira R. L. T.; Caramori G. F.; Galembeck S. E.; Huguenin F. The Nature of the Interactions between Pt4 Cluster and the Adsorbates · H, · OH, and H2O. J. Phys. Chem. A 2008, 112, 11731–11743. 10.1021/jp8033177. 18942818

[ref77] Lushchikova O. V.; Szalay M.; Tahmasbi H.; Juurlink L. B. F.; Meyer J.; Höltzl T.; Bakker J. M. IR Spectroscopic Characterization of the Co-Adsorption of CO2 and H2 onto Cationic Cun+ Clusters. Phys. Chem. Chem. Phys. 2021, 23, 26661–26673. 10.1039/D1CP03119H. 34709259 PMC8653698

[ref78] Hou G.-L.; Faragó E.; Buzsáki D.; Nyulászi L.; Höltzl T.; Janssens E. Observation of the Reaction Intermediates of Methanol Dehydrogenation by Cationic Vanadium Clusters. Angew. Chem., Int. Ed. 2021, 60, 4756–4763. 10.1002/anie.202011109. 33200509

[ref79] Szalay M.; Buzsáki D.; Barabás J.; Faragó E.; Janssens E.; Nyulászi L.; Höltzl T. Screening of Transition Metal Doped Copper Clusters for CO2 Activation. Phys. Chem. Chem. Phys. 2021, 23, 21738–21747. 10.1039/D1CP02220B. 34549207

[ref80] Mayer I. Charge, Bond Order and Valence in the Ab Initio SCF Theory. Chem. Phys. Lett. 1983, 97, 270–274. 10.1016/0009-2614(83)80005-0.

[ref81] Mayer I. Bond Order and Valence Indices: A Personal Account. J. Comput. Chem. 2007, 28, 204 10.1002/jcc.20494. 17066501

[ref82] Megha; Mondal K.; Ghanty T. K.; Banerjee A. Adsorption and Activation of CO2 on Small-Sized Cu–Zr Bimetallic Clusters. J. Phys. Chem. A 2021, 125, 2558–2572. 10.1021/acs.jpca.1c00751. 33728907

[ref83] Barabas J.; Ferrari P.; Kaydashev V.; Vanbuel J.; Janssens E.; Höltzl T. The Effect of Size, Charge State and Composition on the Binding of Propene to Yttrium-doped Gold Clusters. Rsc Advances 2021, 11, 29186–29195. 10.1039/D1RA03262C. 35492069 PMC9040652

[ref84] Mayer I. Covalent Bonding: The Role of Exchange Effects. J. Phys. Chem. A 2014, 118, 2543–2546. 10.1021/jp501232u. 24625298

[ref85] Zhao L.; von Hopffgarten M.; Andrada D. M.; Frenking G. Energy Decomposition Analysis. WIREs Computational Molecular Science 2018, 8, e1345 10.1002/wcms.1345.

[ref86] Khaliullin R. Z.; Cobar E. A.; Lochan R. C.; Bell A. T.; Head-Gordon M. Unravelling the Origin of Intermolecular Interactions Using Absolutely Localized Molecular Orbitals. J. Phys. Chem. A 2007, 111, 8753–8765. 10.1021/jp073685z. 17655284

[ref87] Hopffgarten M. v.; Frenking G. Energy Decomposition Analysis. Wiley Interdisciplinary Reviews: Computational Molecular Science 2012, 2, 43–62. 10.1002/wcms.71.

[ref88] Levine D. S.; Head-Gordon M. Energy Decomposition Analysis of Single Bonds within Kohn-Sham Density Functional Theory. Proc. Natl. Acad. Sci. U. S. A. 2017, 114, 12649–12656. 10.1073/pnas.1715763114. 29158379 PMC5715786

[ref89] Horn P. R.; Head-Gordon M. Polarization Contributions to Intermolecular Interactions Revisited with Fragment Electric-Field Response Functions. J. Chem. Phys. 2015, 143, 114111 10.1063/1.4930534. 26395691

[ref90] Khaliullin R. Z.; Bell A. T.; Head-Gordon M. Analysis of Charge Transfer Effects in Molecular Complexes Based on Absolutely Localized Molecular orbitals. J. Chem. Phys. 2008, 128, 184112 10.1063/1.2912041. 18532804

[ref91] Veccham S. P.; Lee J.; Mao Y.; Horn P. R.; Head-Gordon M. A Non-Perturbative Pairwise-Additive Analysis of Charge Transfer Contributions to Intermolecular Interaction Energies. Phys. Chem. Chem. Phys. 2021, 23, 928–943. 10.1039/D0CP05852A. 33355325

[ref92] Barhács B.; Janssens E.; Höltzl T. C2 Product Formation in the CO2 Electroreduction on Boron-Doped Graphene Anchored Copper Clusters. Phys. Chem. Chem. Phys. 2022, 24, 21417–21426. 10.1039/D2CP01316A. 36047512

[ref93] Mondal K.; Megha; Banerjee A.; Fortunelli A. Adsorption and Activation of CO2 on a Au19Pt Subnanometer Cluster in Aqueous Environment. Computational and Theoretical Chemistry 2022, 1212, 113701 10.1016/j.comptc.2022.113701.

[ref94] Saputro A. G.; Agusta M. K.; Wungu T. D. K.; Suprijadi; Rusydi F.; Dipojono H. K. DFT Study of Adsorption of *CO*_2_ on Palladium Cluster Doped by Transition Metal. Journal of Physics: Conference Series 2016, 739, 012083 10.1088/1742-6596/739/1/012083.

[ref95] Saputro A. G.; Putra R. I.; Maulana A. L.; Karami M. U.; Pradana M. R.; Agusta M. K.; Dipojono H. K.; Kasai H. Theoretical Study of CO2 Hydrogenation to Methanol on Isolated Small Pdx Clusters. Journal of Energy Chemistry 2019, 35, 79–87. 10.1016/j.jechem.2018.11.005.

[ref96] Chen W.; Cao J.; Fu W.; Zhang J.; Qian G.; Yang J.; Chen D.; Zhou X.; Yuan W.; Duan X. Molecular-Level Insights into the Notorious CO Poisoning of Platinum Catalyst. Angew. Chem., Int. Ed. 2022, 61, e202200190 10.1002/anie.202200190. 35132761

[ref97] Imaoka T.; Toyonaga T.; Morita M.; Haruta N.; Yamamoto K. Isomerizations of a Pt4 cluster revealed by spatiotemporal microscopic analysis. Chem. Commun. 2019, 55, 4753–4756. 10.1039/C9CC00530G. 30897188

[ref98] Adams B. D.; Asmussen R. M.; Chen A.; Mawhinney R. C. Interaction of Carbon Monoxide with Small Metal Clusters: a DFT, Electrochemical, and FTIR Study. Can. J. Chem. 2011, 89, 1445–1456. 10.1139/v11-120.

[ref99] Zhai H.; Alexandrova A. N. Fluxionality of Catalytic Clusters: When It Matters and How to Address It. ACS Catal. 2017, 7, 1905–1911. 10.1021/acscatal.6b03243.

[ref100] Yan X.; Hu X.; Fu G.; Xu L.; Lee J.-M.; Tang Y. Facile Synthesis of Porous Pd3Pt Half-Shells with Rich “Active Sites” as Efficient Catalysts for Formic Acid Oxidation. Small 2018, 14, 1703940 10.1002/smll.201703940. 29409151

