# Supplementary material for: Multistep CO2 Activation and Dissociation Mechanisms on PdxPt4–x Clusters in the Gas Phase
Source: J Phys Chem A. 2023 May 17;127(21):4596–608. doi: 10.1021/acs.jpca.2c08333 (PMC10240495; doi:10.1021/acs.jpca.2c08333)
Supplement: Supplementary file 1 — jp2c08333_si_001.pdf [file jp2c08333_si_001.pdf]

Supporting Information: Multi-step CO_2 Activation and Dissociation Mechanisms on Pd_xPt_{4-x} Clusters in the Gas Phase

Renata Sechi[†,¶] and Tibor Höltzl[*,†,‡,¶]

†*Furukawa Electric Institute of Technology, Késmárk utca 28/A, Budapest, 1158, Hungary*

‡*MTA-BME Computation Driven Chemistry Research Group, Department of Inorganic and Analytical Chemistry, Budapest University of Technology and Economics, Műegyetem rkp. 3, Budapest, 1111, Hungary*

¶*Department of Inorganic and Analytical Chemistry, Budapest University of Technology and Economics, Szent Gellért tér 4, Budapest, 1111 Hungary*

E-mail: tibor.holtzl@furukawaelectric.com

Test of Spin multiplicity and basis-sets benchmarking on bare Pd_xPt_{4-x} clusters

In the following we report the results of the optimization of the different clusters. We consider four starting configurations for the Pd_xPt_{4-x} clusters: a chain (fig. S1a), a quadrangle (fig. S1b), a triangle (fig. S1c), a tetrahedron (fig. S1d). We generated the starting geometries usuign the IQmol software and applied consecutively three basis sets to optimize them: LANL2DZ, def2-SVP, def2-TZVP. Moreover, we tested different spin multiplicities (1, 3, 5), based the on gas phase results reported in[1,2]. We computed the atomization E_{atom} energy

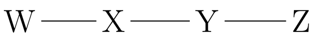

(a) chain

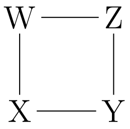

(b) quadrangle

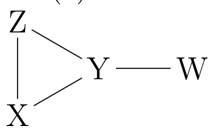

(c) triangular

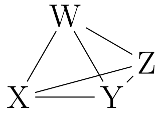

(d) tetrahedron

Figure S1: Tetranuclear starting structures for the clusters' geometry optimization in the gas phase. W, X, Y, Z = Pd/Pt atom.

of the optimized structures as

$$E_{atom}(Pd_xPt_{4-x}) = \frac{E_{mol}(Pd_xPt_{4-x}) - x \cdot E(Pd) - (4-x) \cdot E(Pt)}{4}, \tag{1}$$

where $E_{mol}(Pd_xPt_{4-x})$ is the SCF energy of a cluster composed by x Pd-atoms and $4-x$ Pt-atoms, $x = 1, ..., 4$, $E(Pt)$, $E(Pd)$ are the SCF energies of the Pd and Pt atoms, respectively. First we show the difference of the structures' atomization energy depending on the basis set. Each of the Figures S2 -S12, show the results for one of the starting conformations in FigureS1 and spin multiplicities 1,3, or 5. From this results, we observe that the def2-SVP and LANL2DZ basis sets yield atomization energies higher than the def2-TZVP basis set. For the further computations only the tetrahedron like structures were used, since they were the most stable ones. Even if we started the optimization from another structure, the final optimized cluster structure was often found to exihibit rhomboidal and tetrahedral.

For each starting geometry, we found that the triplet structure is the one with most negative atomization energy (i.e., the most stable structure, energetically). Figures S13-S15 show the atomization energies of the optimized structures for singlet, triplet and quintet spin multiplicities of the clusters, respectively. Even if the initial geometry was different, most of the structures converged to a tetrahedron-like shape, so the energies of the optimized structures are very similar. As stated in the main text, the structures with tetrahedron-like geometry and triplet state are the ones with lowest energy.

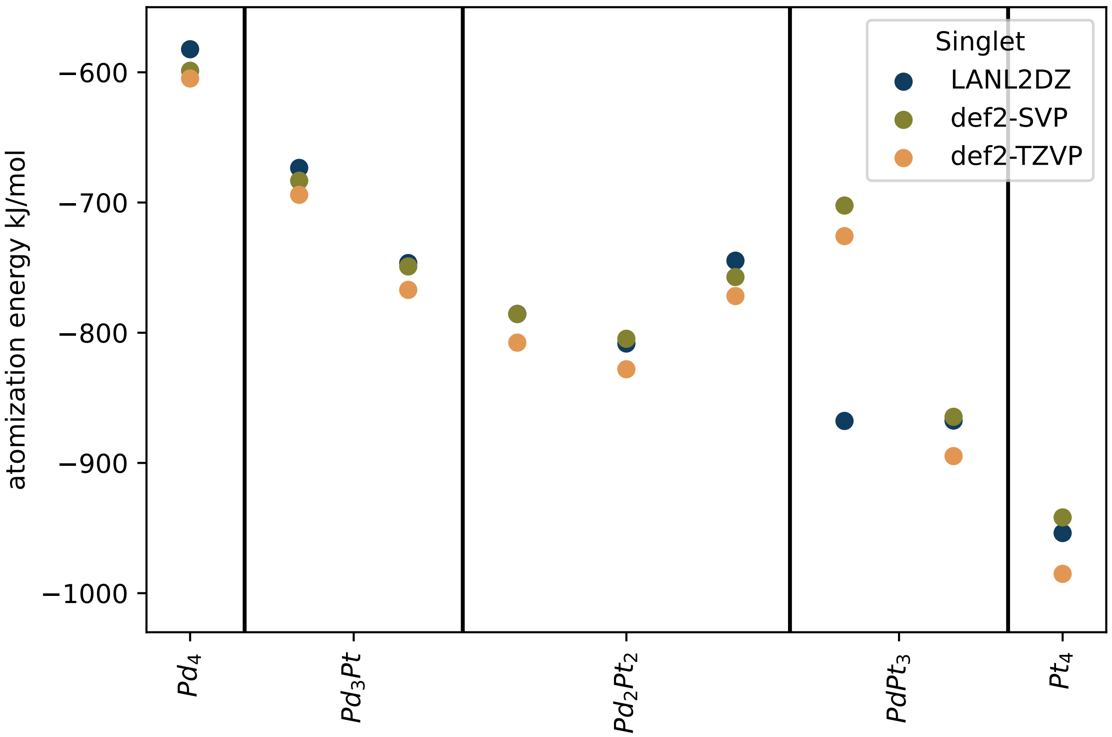

Figure S2: Atomization energy of the chain clusters with different basis sets. (Singlet spin states).

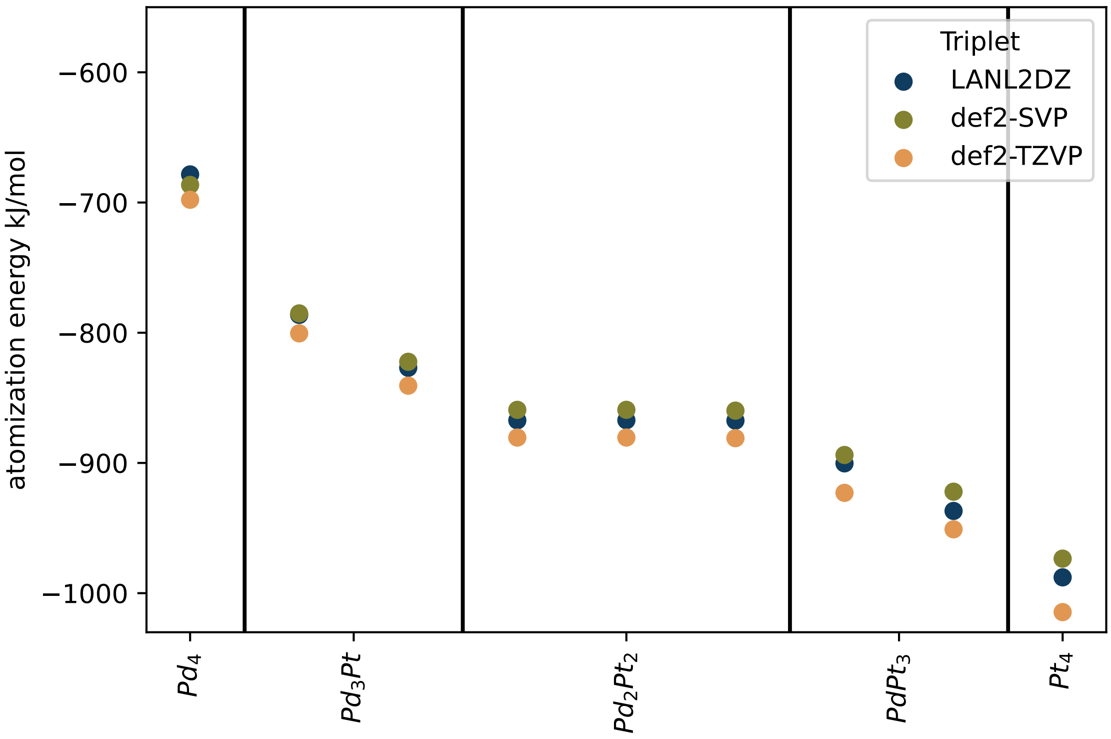

Figure S3: Atomization energy of the chain clusters with different basis sets. (Triplet spin states).

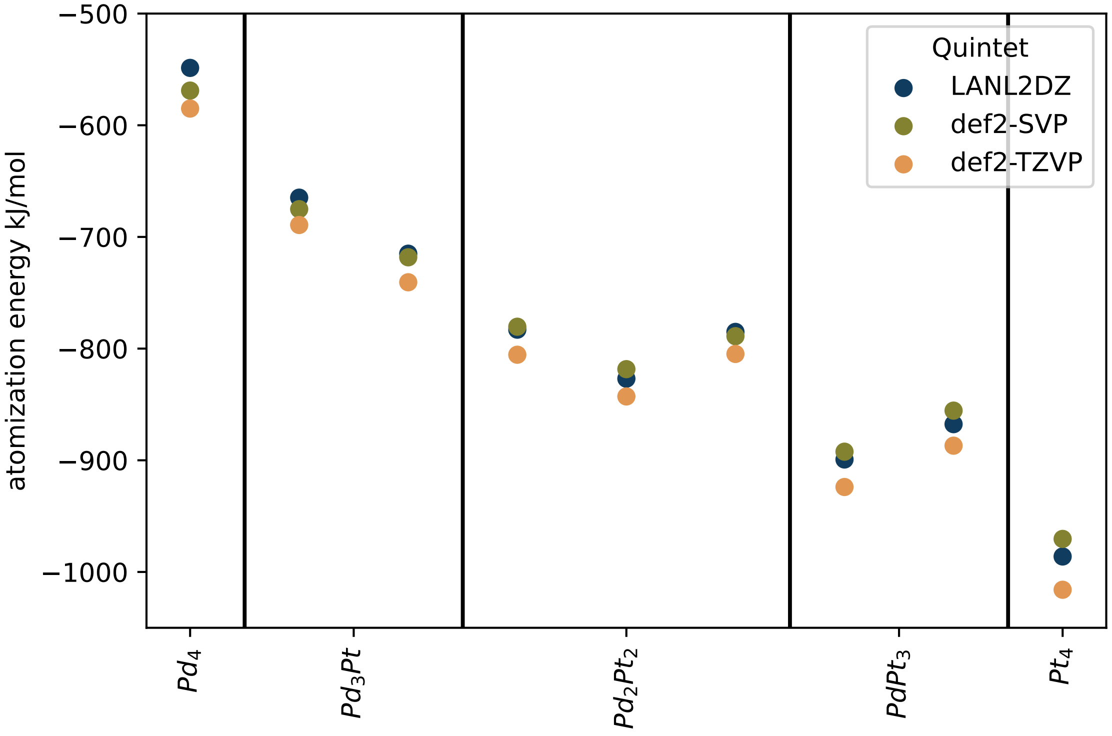

Figure S4: Atomization energy of the chain clusters with different basis sets. (Quintet spin states)

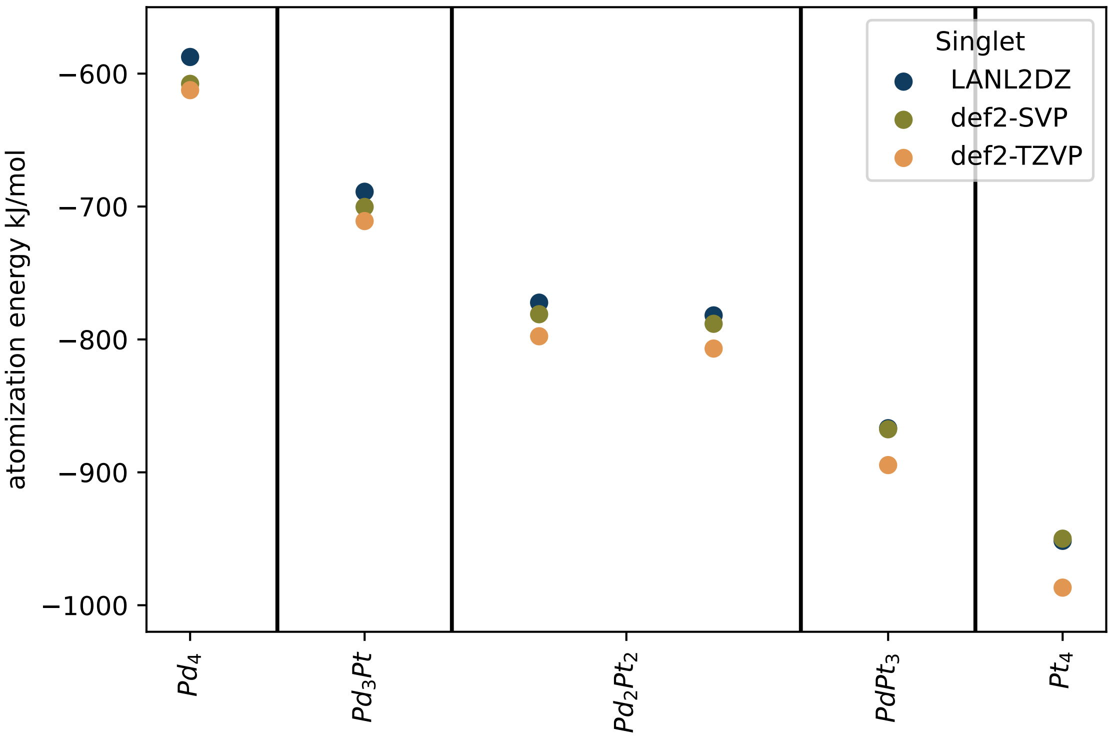

Figure S5: Atomization energy of the quadrangular clusters with different basis sets. (Singlet spin states).

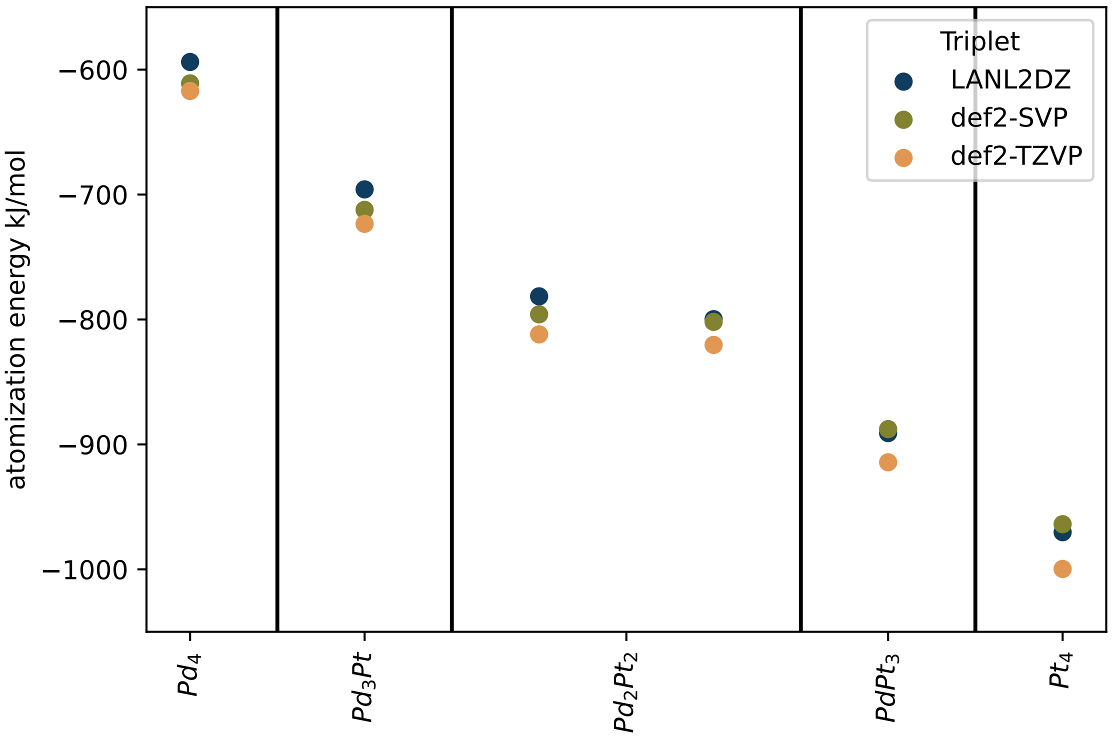

Figure S6: Atomization energy of the quadrangular clusters with different basis sets. (Triplet spin states).

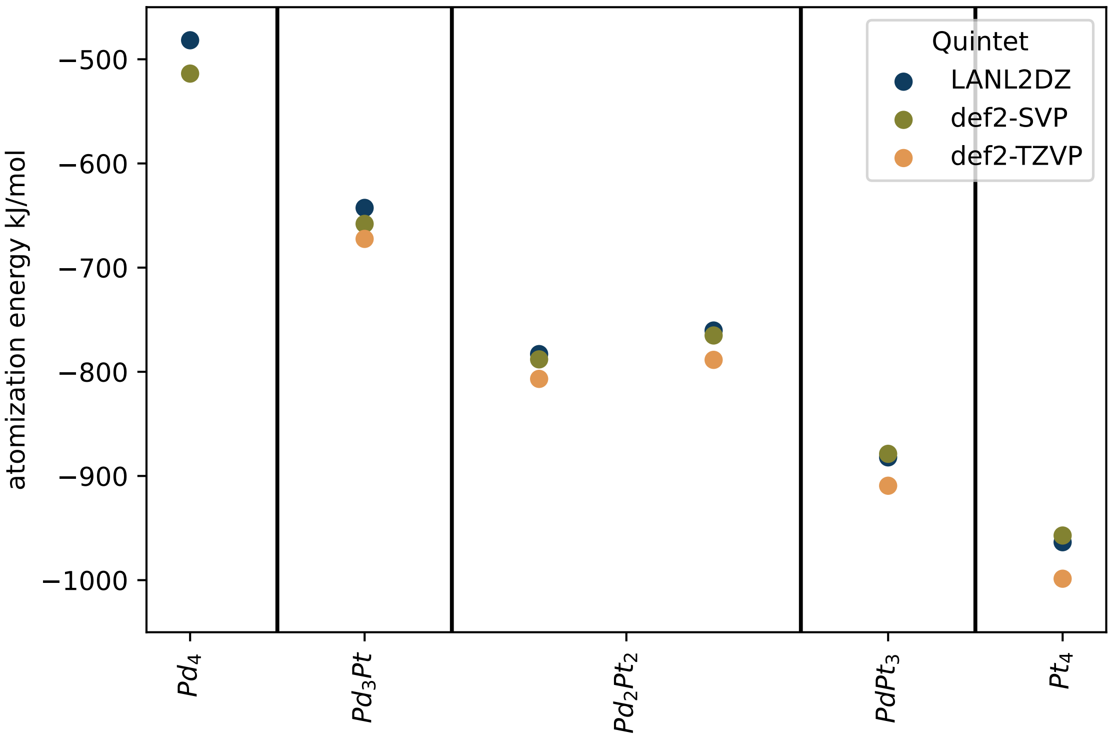

Figure S7: Atomization energy of the quadrangular clusters with different basis sets. (Quintet spin states).

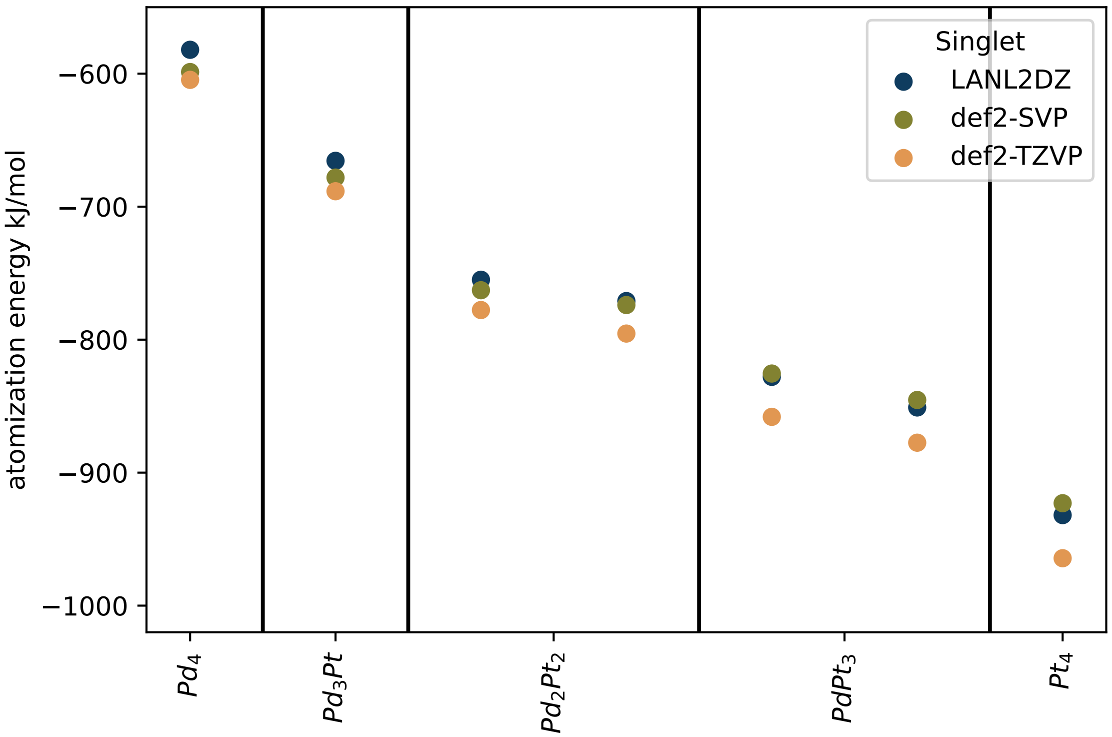

Figure S8: Atomization energy of the triangular clusters with different basis sets. (Singlet spin states).

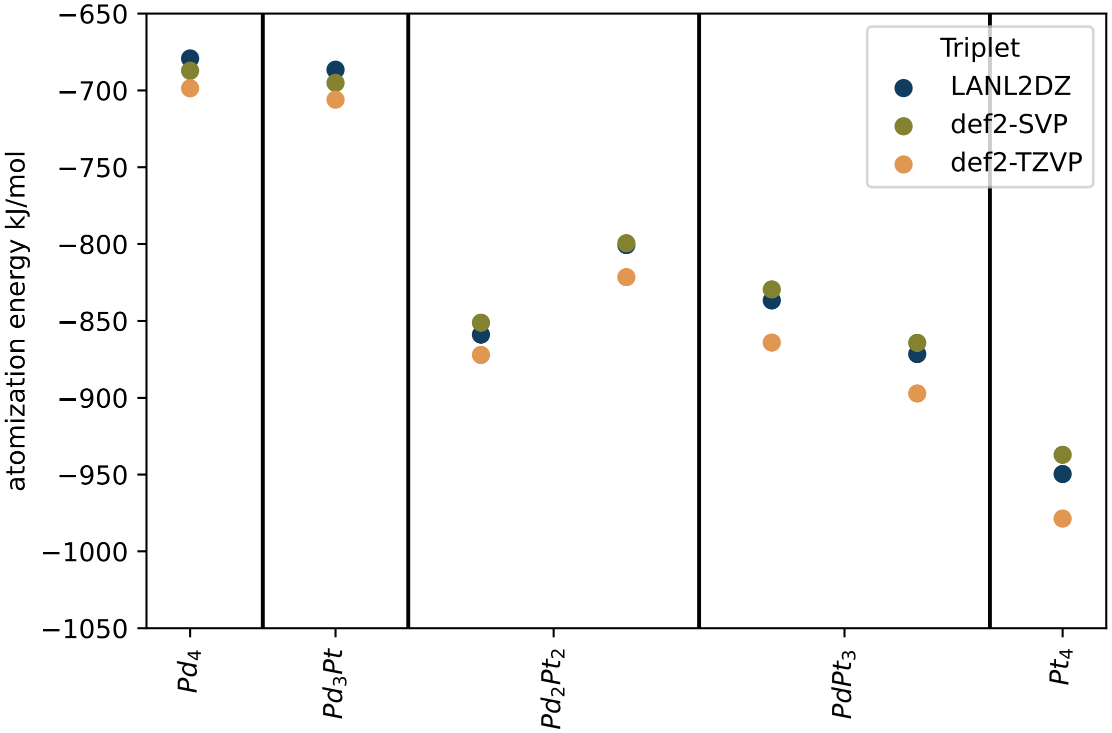

Figure S9: Atomization energy of the triangular clusters with different basis sets. (Triplet spin states).

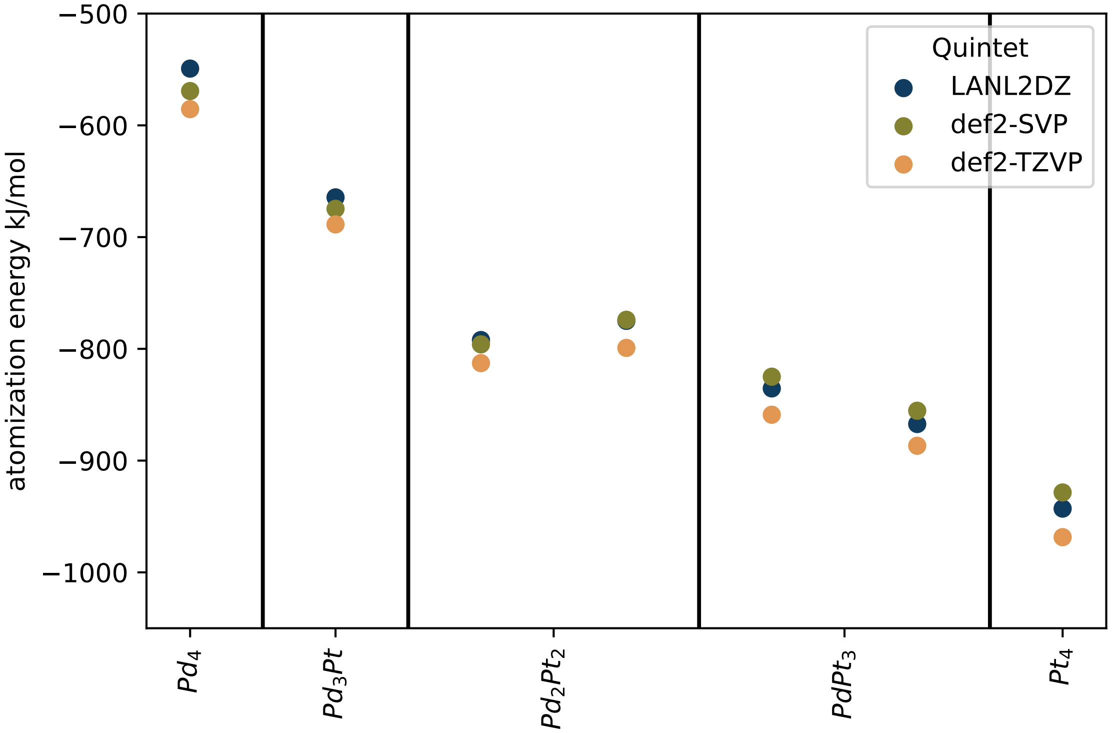

Figure S10: Atomization energy of the triangular clusters with different basis sets. (Quintet spin states).

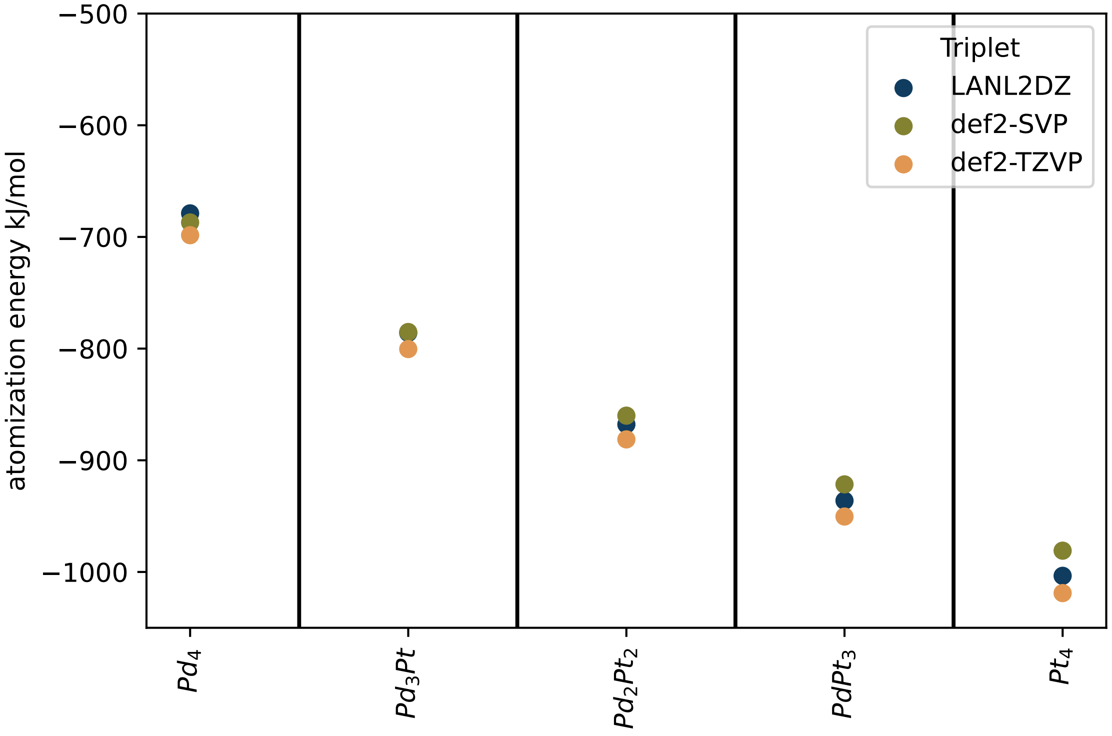

Figure S11: Atomization energy of the tetrahedron-like clusters with different basis sets. (Triplet spin states)

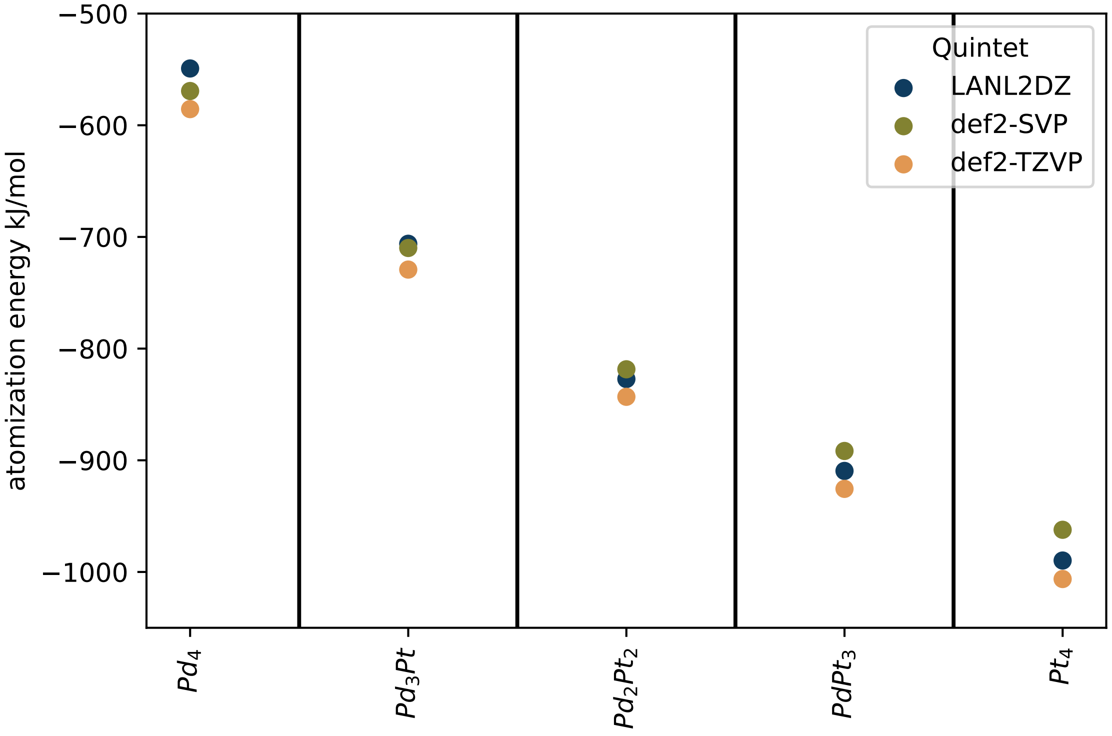

Figure S12: Atomization energy of the tetrahedron-like clusters with different basis sets. (Quintet spin states).

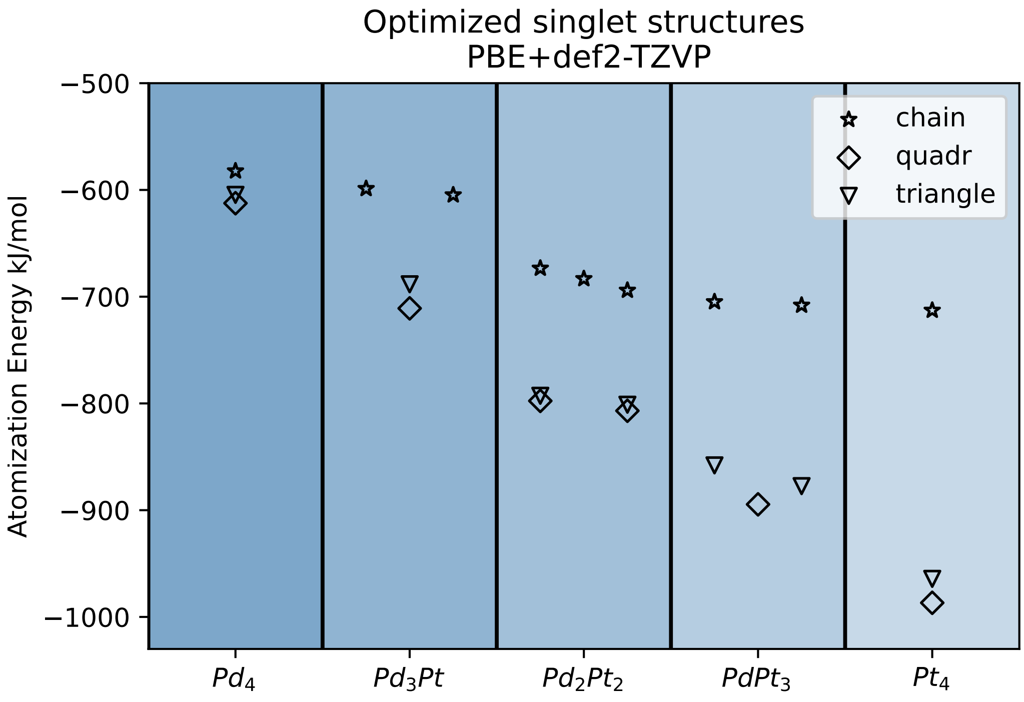

Figure S13: Atomization energy of the optimized cluster structures in singlet state. The singlet tetrahedron-shaped structures are missing.

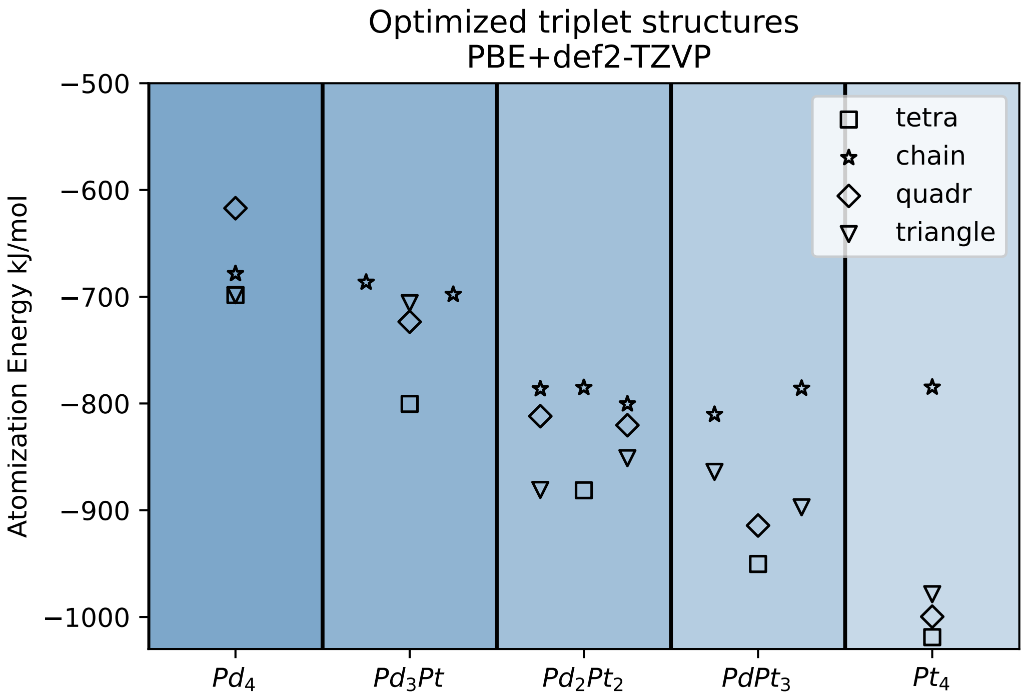

Figure S14: Atomization energy of the optimized cluster structures in triplet state.

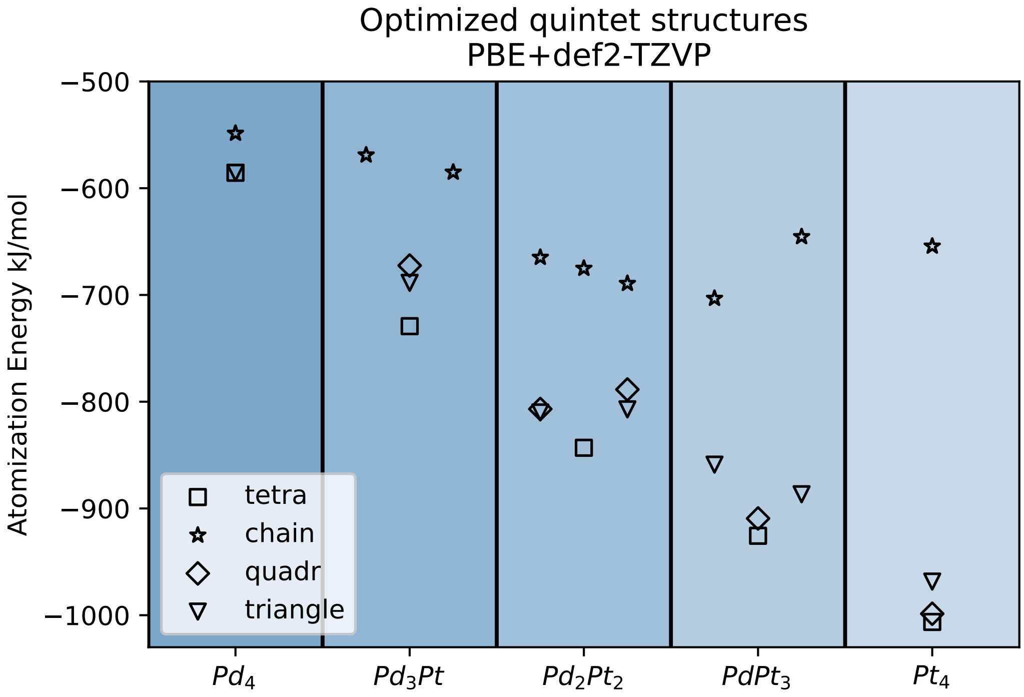

Figure S15: Atomization energy of the optimized cluster structures in quintet state.

Pd_xPt_{4-x}+CO_2 structures

We generated CO_2 cluster adducts considering six binding modes, displayed in Figure S16 (intact CO_2) and Figure S17 (dissociated CO_2). Different basis-sets have been used for the bare-cluster structure optimization, as mentioned in Section Computational Methods in the main text.

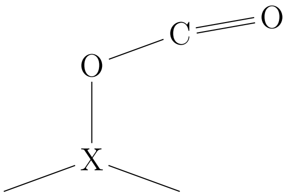

(a) Intact i

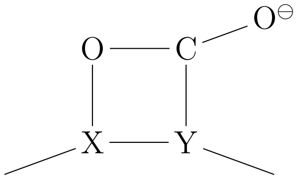

(b) Intact ii

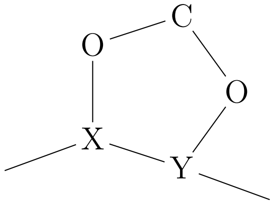

(c) Intact iii

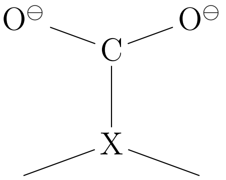

(d) Intact iv

Figure S16: Schematized intact CO_2-binding mechanisms to cluster.

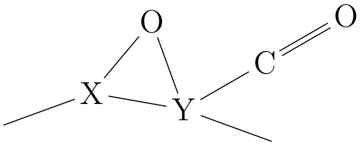

(a) Dissociated v

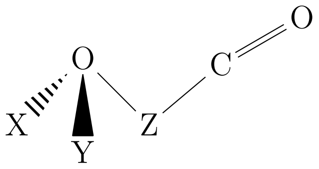

(b) Dissociated vi

Figure S17: Schematized dissociated CO_2 binding modes to cluster.

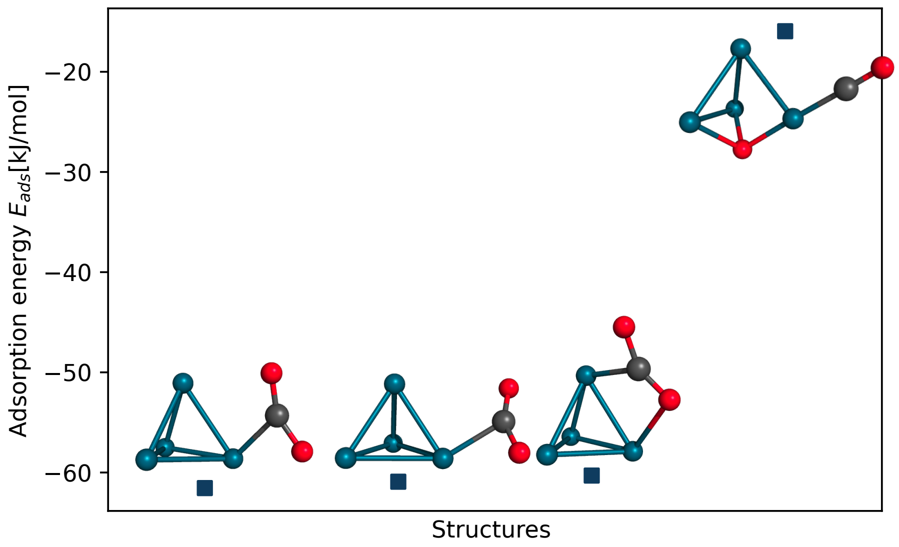

Figure S18: Lowest-adsorption energy structures of Pd_4 cluster

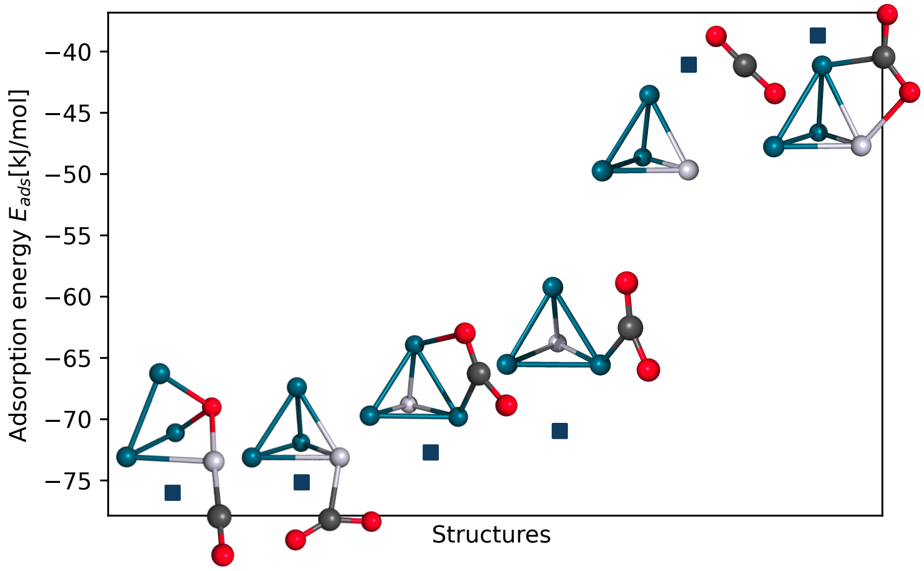

Figure S19: Lowest-adsorption energy structures of Pd_3Pt cluster

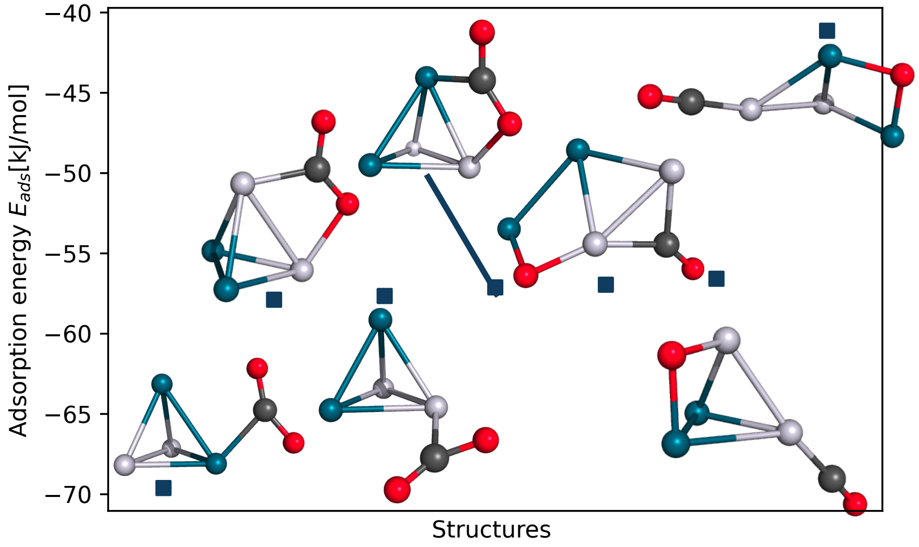

Figure S20: Lowest-adsorption energy structures of Pd_2Pt_2 cluster

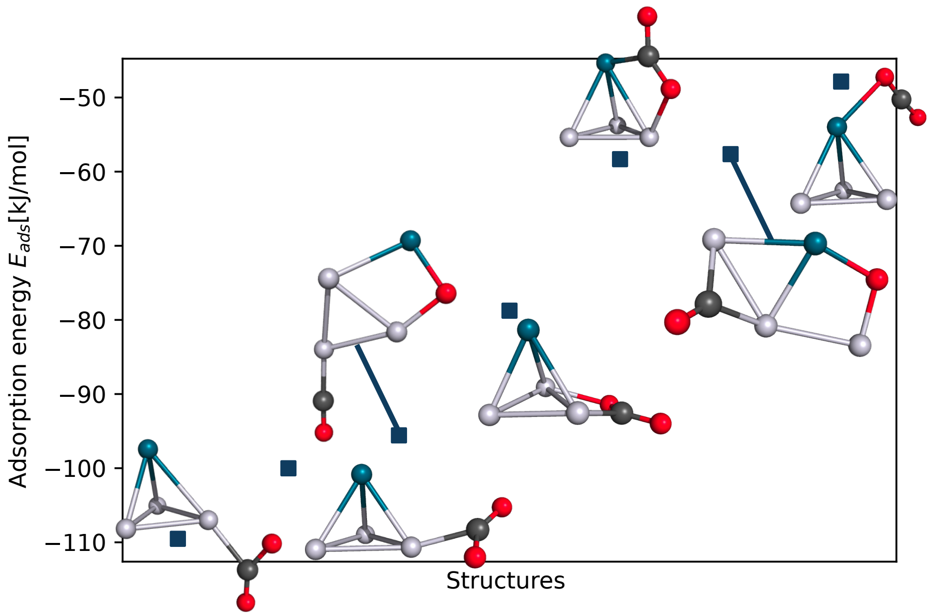

Figure S21: Lowest-adsorption energy structures of $PdPt_3$ cluster

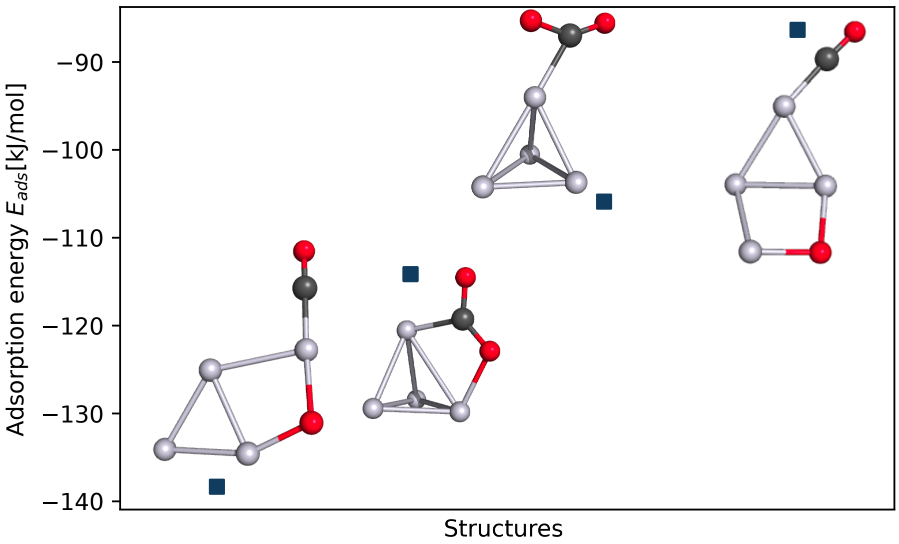

Figure S22: Lowest-adsorption energy structures of Pt_4 cluster

Benchmarking of Pt_4 geometry and spin multiplicity

We analyzed the stabilizes of the quadrangular and tetrahedron-like structures with spin multiplicities 3 and 5 using various Density Functionals, comparing to high level CCSD(T) computations.

We optimized the structure using the def2-TZVPP, def2-QZVP and also the def2-QZVPP basis sets in conjunction with the PBE functional, including D3 dispersion correction (Figure S23). Subsequently, to test how the choice of the exchange.correlation functional influences the results, we re-optimized the cluster structures with the def2-QZVPP basis set and the B3LYP, TPSS, TPSSh functionals (Figure S24). We report in the following the values of the bond lenghts and angles for the Pt_4 isomers. As last step of our analysis, we computed the CCSD(T)/def2-QZVPP single-point energies of the structures optimized by PBE-D3/def2-TZVP method. The results are also shown in Figure S24. Our results infer that the triplet multiplicity, tetrahedron-like structure has lower energy then the quintet state quadrangular structure. Furthermore, we observe that the description given by the TPSSh functional is the one that is closer to the CC energy. We utilize this knowledge for compute with TPSSh+D3/def2-QZVPP level of theory the single point energies of the single cluster and CO_2 structures, the highest energy-barrier structure and the most stable product in the main text, see Figure S31.

We use the T_1 diagnostics to estimate the possible extent of multireference character of the Pt_4 molecules. Table 1 shows the results for the computations. All the values we obtained are larger than 0.17 (quadrangular quintet). Typically, T_1 values smaller than 0.02 indicate that single reference methods perform well[3]. However, it has been reported for 4d transition metal clusters and their oxides, hydrides, and metal halides that the 0.02 value is not practical for 4d TM-containing species and propose $T_1 < 0.045$[4]. Yet, our calculations are far from this value. These indicates a possible multireference charachter of all the computed structures.

Table 1: T_1 descriptors for the Pt_4 CCSD(T)/def2-QZVPP computations

Pt_4 geometry	$T1$
quadr, 3	0.2702
quadr, 5	0.1756
tetra, 3	0.2429
tetra, 5	0.2021

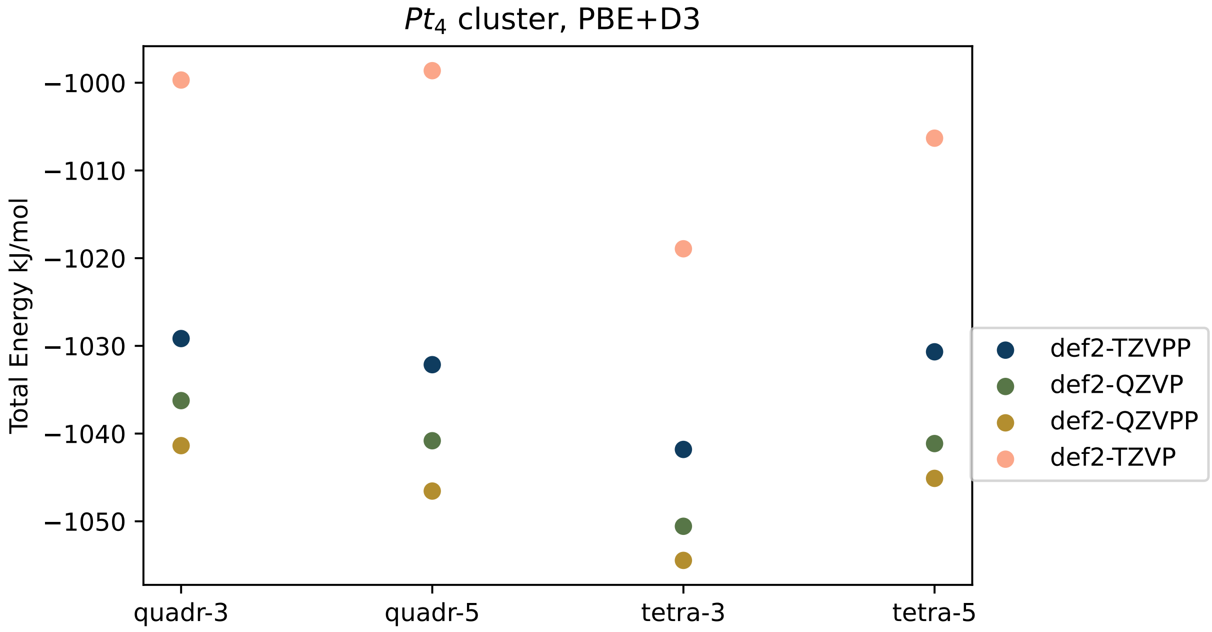

Figure S23: Test of Pt_4 with different basis sets.

In a similar way as for the bare Pt_4 cluster, we screened the aforementioned functional and basis sets for the Pt_4+CO_2 adduct with lowest energy, considering two adducts, respectively with intact and dissociated CO_2 bond to the transition metal cluster. The results can be found in Figures S25 and S26. Interestingly, the quadrangular, triplet structure exhibit a square with point-group symmetry D_{4h}, while the quadrangular quintet structure is a rectangle with point-group D_{2h}. Furthermore, the tetrahedron-like geometries are not a perfect tetrahedron; the difference between the structure in spin 3 and spin 5 is that the triplet structure is more regular. It has angles spacing from 58-60 degrees and bond lengths from 2.57 to 2.69 Å, whereas for the quintet structure these differences are larger (57-62 degrees, 2.50-2.63 Å).

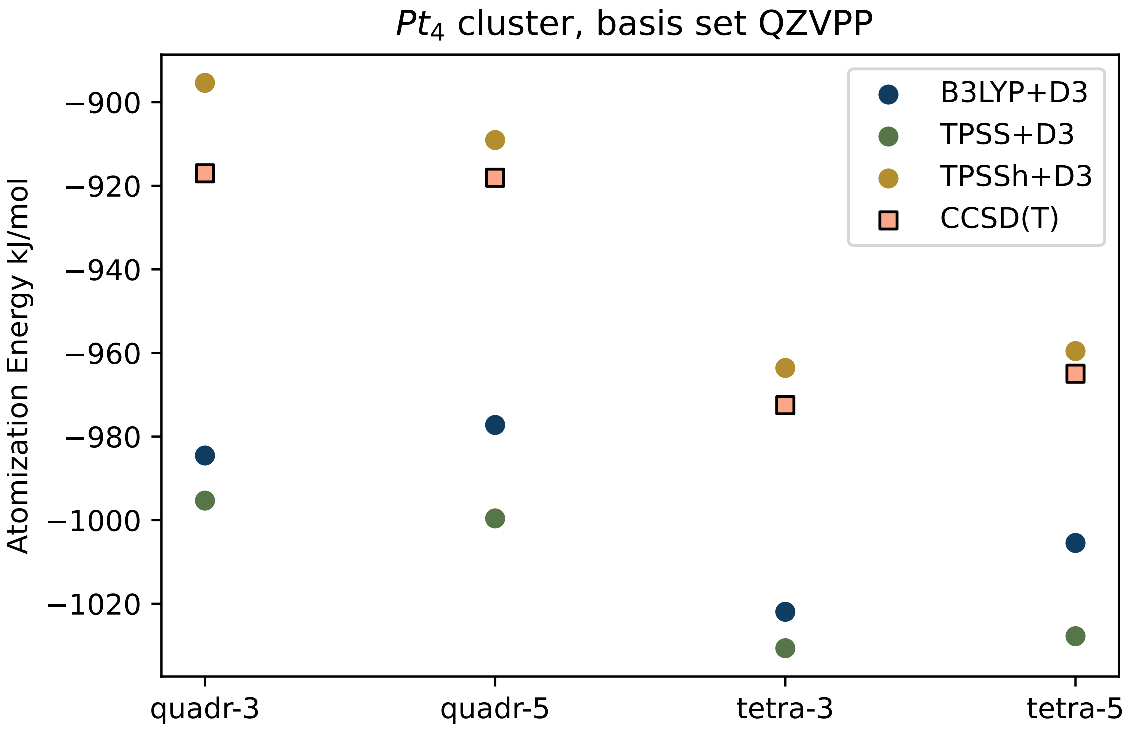

Figure S24: Test of Pt_4 with different functionals.

Testing spin multiplicity and CO_2 binding modes

We optimized the Pd_2Pt_2 CO_2 adduct structures for different spin multiplicities (1, 3, 5). The aim of our test was to examine whether the cluster keeps the triplet ground state upon CO_2 adsorption. We tested the starting structure of the intact and dissociated CO_2 binding modes; our computations showed that for the CO_2 binding modes the triplet structures have lower energy than the singlet and quintet adducts. For the dissociated-CO_2 binding modes, we found a few exceptions, however those few structures have very high energy and we excluded them from the list potential CO_2 activation or dissociation reaction intermediates. The level of theory used for the calculations is PBE+D3/def2-TZVP. Our results are reported in FigureS27.

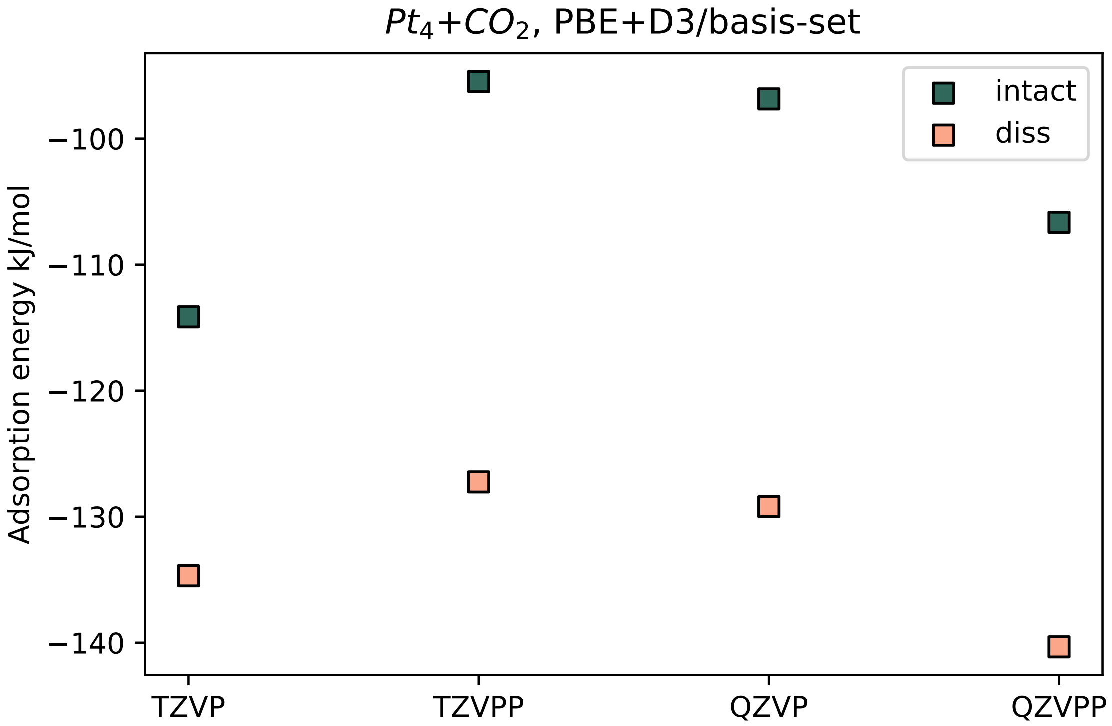

Figure S25: Test of CO_2 intact and CO_2 dissociated Pt_4 adducts with different basis sets.

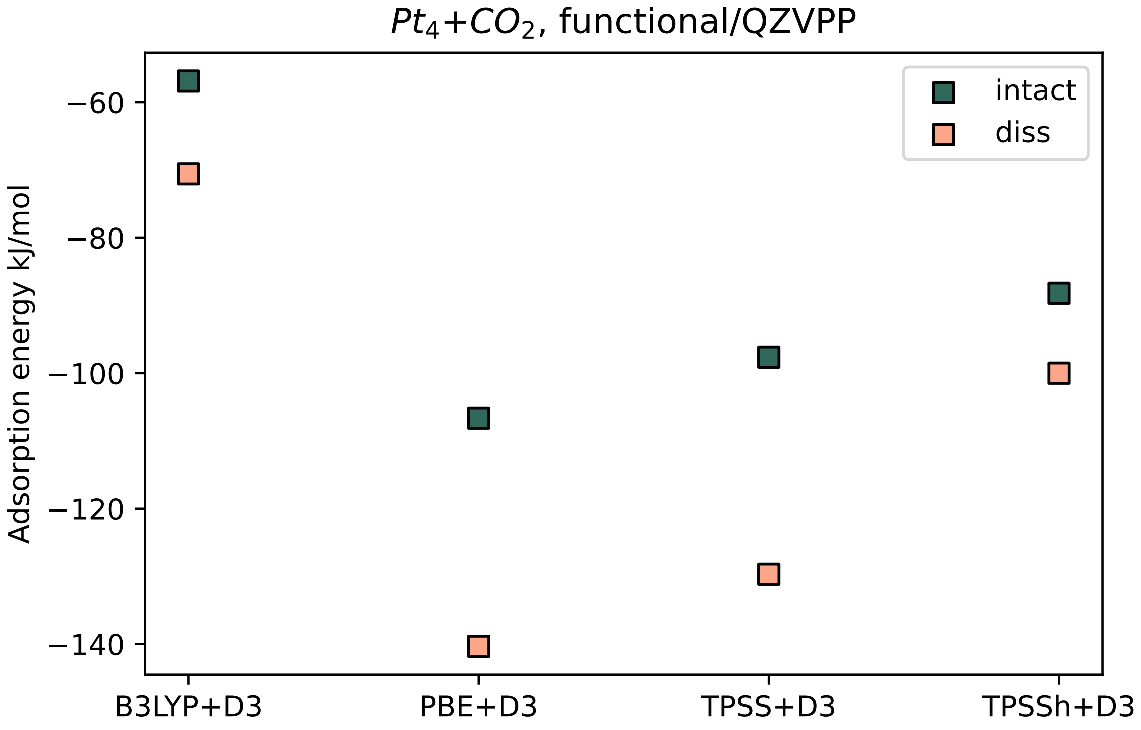

Figure S26: Test of CO_2 intact and CO_2 dissociated Pt_4 with different functionals.

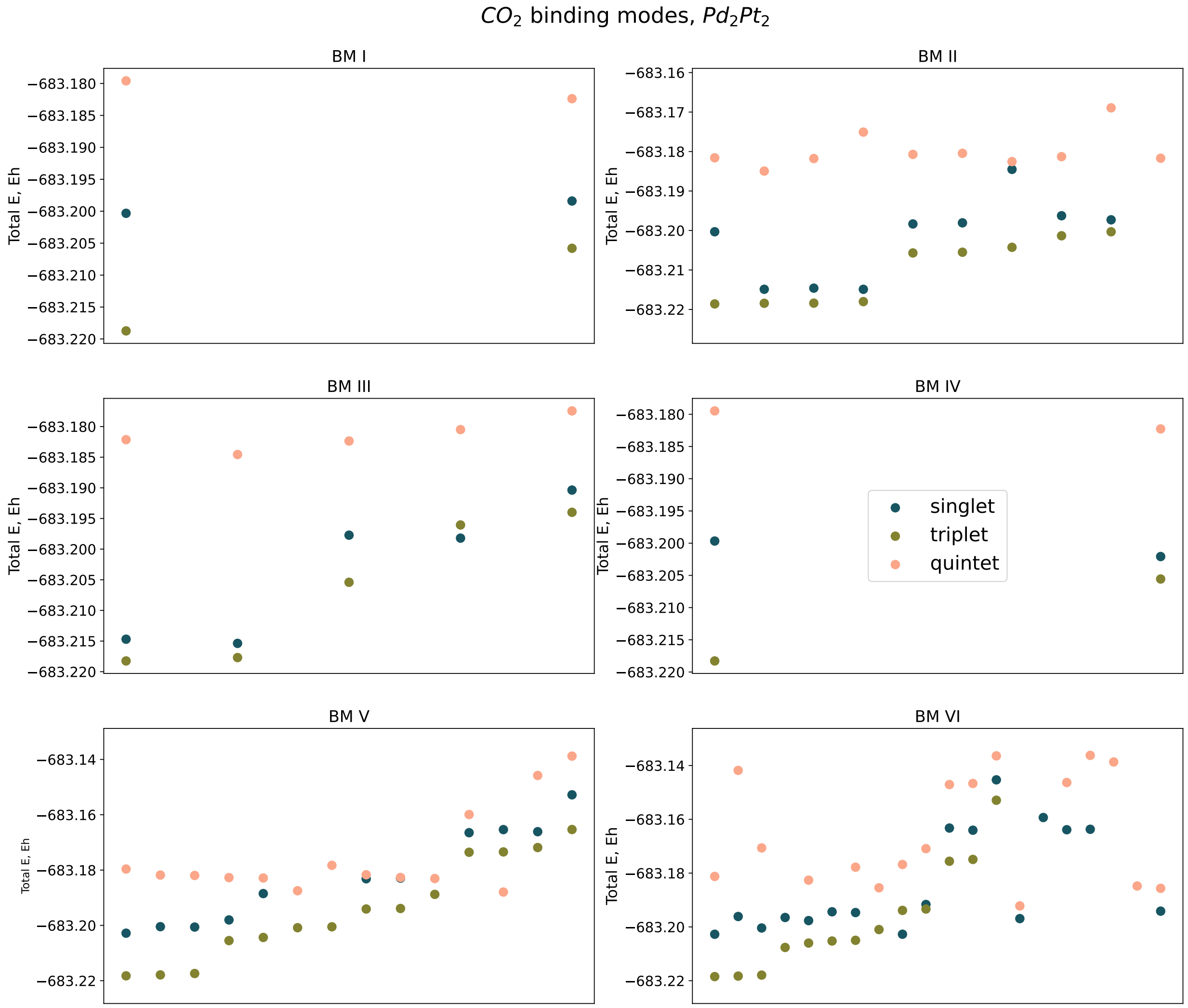

Figure S27: Test of the energy of the geometry-optimized adducts for all the CO_2 binding modes. The triplet structures have lower energy.

Testing the accuracy of the PBE-D3/def2-TZVP method for the CO_2 binding energies

Table 2: CO_2 binding energies of the first intermediates along the reaction paths (kJ/mol).

	CCSD(T)/def2-TZVPP	CCSD(fT)/def2-TZVPP	PBE-D3/def2-TZVP
Pd_4CO_2	+22	+22	-21
Pt_4CO_2	-84	-75	-114

We tested the accuracy of the applied PBE-D3/def2-TZVP method compared to coupled-cluster reference values for the CO_2 binding energies of the first intermediate along the reaction paths. Please note that as it is described in the main text, the T_1^2 diagnostics indicates possible multi-reference characters of the cluster's electronic structure. Indeed, it has been shown earlier by multi-reference complete active space self-consistent field computations that there are 44 and 51 electronic states within 2.2eV and 1.2 eV above the ground state in the case of Pd_4 and Pt_4, respectively[5], and the ground and the first excited states are quasi-degenerate. Thus, apart from the conventional CCSD(T) computations (where the CC equations were solved using the Cholesky decomposition using the default settings in the Q-Chem code), we also used the CCSD(fT) method (which is equivalent to the $CR - CCSD(T)_2$)[6], what in several cases can describe also strongly correlated electronic structure. Nevertheless, it is important to note, that still these computations are not of benchmark quality, but are expected to provide correct tendencies. It is interesting to note that the CCSD(T) and the CCSD(fT) computations yield similar results.

The results above show that while the coupled cluster and the PBE-D3 DFT results are quantitatively different by up to 43 kJ/mol, the trends are correct, which supports the dopant-dependent binding tendencies shown in our manuscript.

Mayer Bond Orders

To comparison to the adducts' Mayer Bond Order, in figure S28 we present the Mayer bond orders of the cluster structures without CO_2.

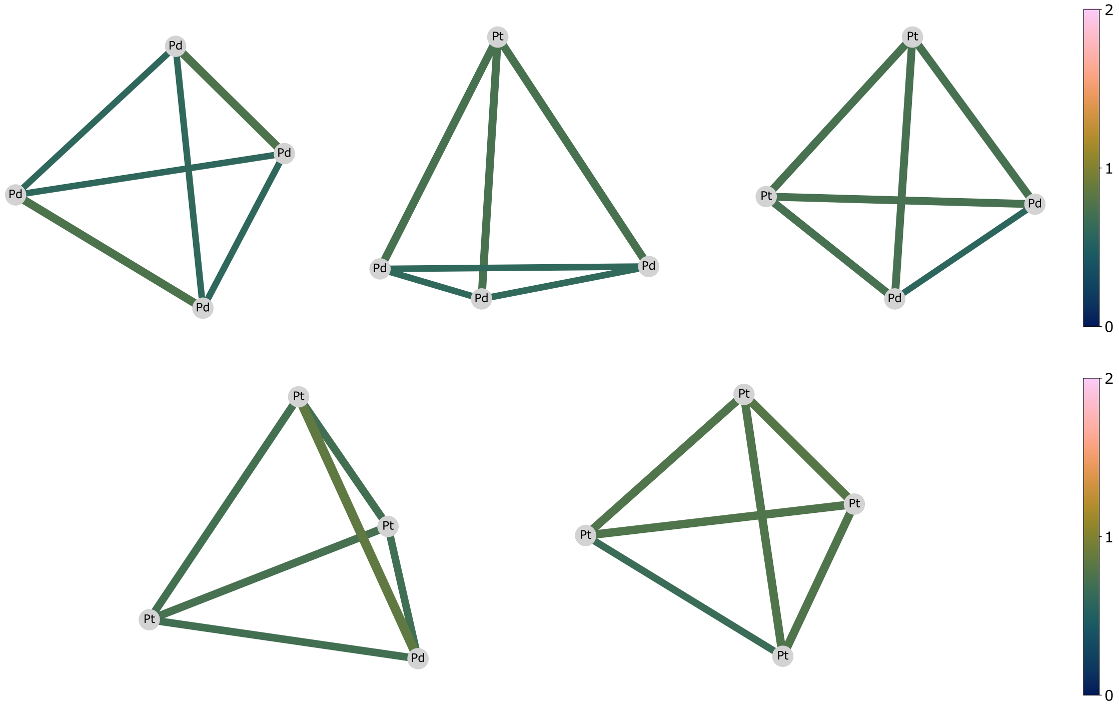

Figure S28: Mayer bond orders of the triplet tetrahedron structures for each cluster composition.

Table 3: Natural Charges of the sample adduct structures.

	Pd4	Pd3Pt	Pd2Pt2	PdPt3	Pt4
O	-0.52397	-0.52397	-0.58062	-0.58807	-0.5444
C	0.72188	0.72188	0.64872	0.64997	0.63978
O	-0.51105	-0.51105	-0.48785	-0.48805	-0.47118

EDA

In this section, we provide additional information from the EDA computations. We compared the results of the charge transfer from the TM cluster to the CO_2 moiety in the cluster-intact

CO_2 adducts.

In Figure S29b the charge transfer energy is related to the total dipole moment of the TM clusters in the gas phase. We observed that with higher TM dipole moment, the more structures we found. This means that the number of CO_2 binding sites on a cluster is related to the total dipole moment of the cluster itself.

We display in Figure S29a the mean charge-transfer energy and the energy of the HOMO, LUMO levels of the bare clusters. The values of the HOMO-LUMO gap are very similar ($|-0.2| \pm 0.02$ a.u.) for Pd_3Pt, Pd_2Pt_2,$PdPt_3$, whereas the mean charge transfer goes from - 510 to -320 kJ/mol. In Figure S30, we display the sum of the interaction and relaxation energies as function of the charge transfer. We cannot find any relationship between these two quantities, as mentioned in the main text. On the one hand, the relaxation energy is a quantity depending on the geometry of the adduct and on the geometry of the optimized free fragment, whereas charge transfer and interaction energy only depend on the final adduct. On the other hand, the interaction energy is also depending on the preparation energy, which has a large contributions in adducts with dissociated CO_2. As a result, we do not find the adsorption energy by summing interaction and relaxation energy in figure S30.

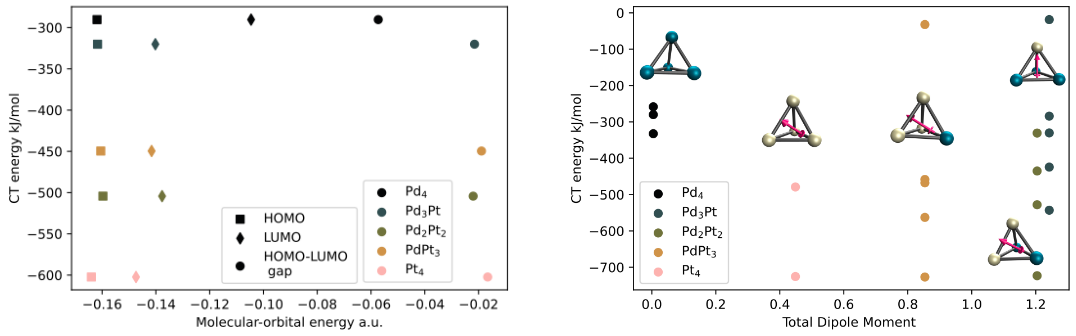

(a) Mean Charge Transfer as function of HOMO, LUMO, HOMO-LUMO gap energy

(b) Charge Transfer as function of total dipole moment of the bar cluster.

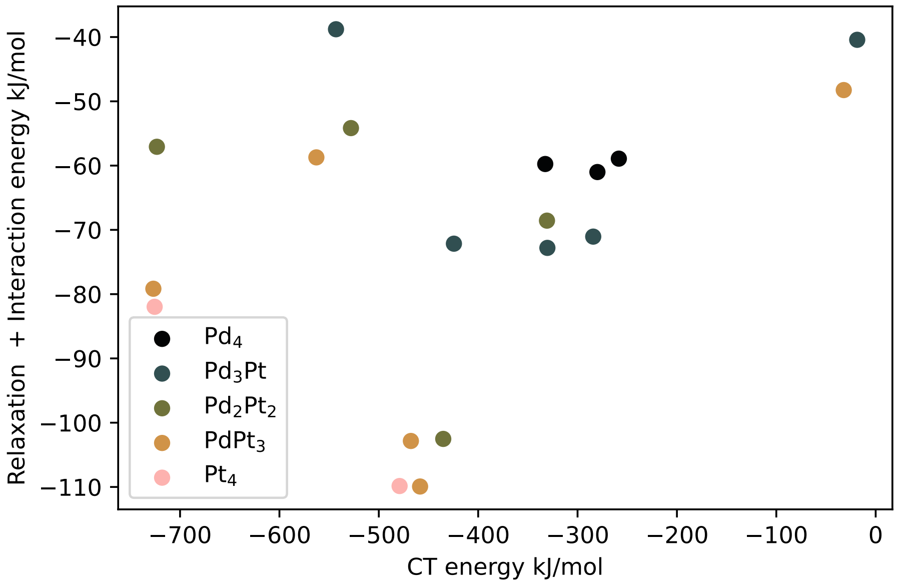

Figure S30: Sum of interaction and relaxation energy as function of the charge transfer. No correlation is observed.

Direct CO_2 dissociation from the reaction paths

In Figure S31, we compare the relative energies of the highest lying transition structures, and the lowest-energy product structures to that of the separated clusters and CO_2. We cannot find a linear scaling of the relative energy of the dissociation barrier with the Pt-doping as reported in[7]. In order to be more accurate when comparing the three energies for each cluster, the single point energies of the structures in Figure were calculated by TPSSh+D3/def2-QZVPP level of theory. Based on our benchmark studies on Pt_4 clusters (see Section Benchmarking Pt_4), the TPSSh+D3/def2-QZVPP level of theory describes the clusters with a similar energy to the CSSD(T) calculations.

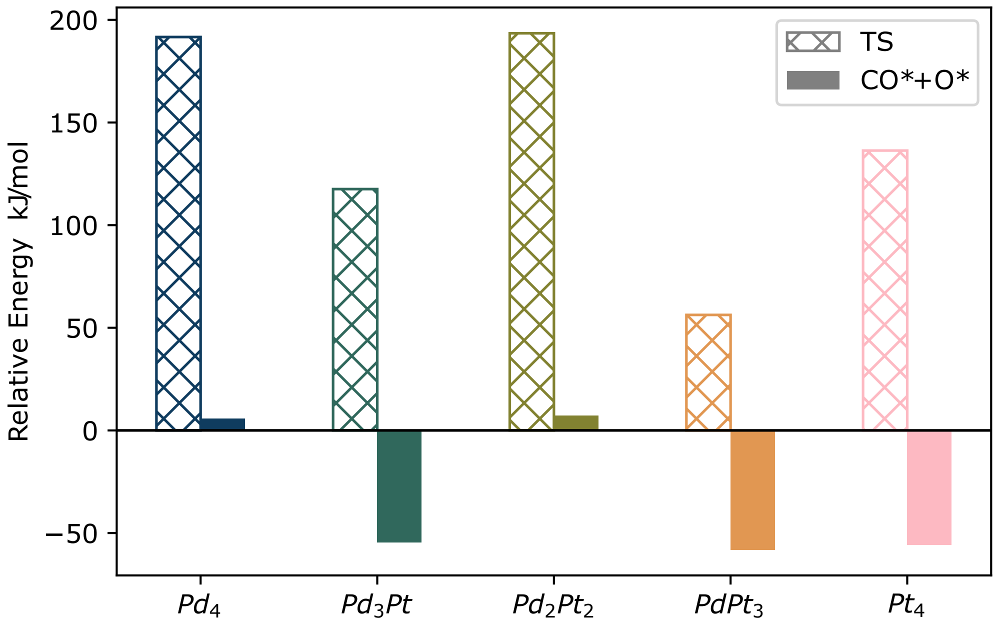

Figure S31: Relative energy to separated cluster and CO_2 of highest-energy transition state (TS), lowest-energy product with dissociated CO_2.

Further AIMD simulations analysis

In section in the main text, we reported the characteristics of the processes observed in the AIMD simulations for each cluster composition. In the following, we describe two different analysis we performed on the trajectories. The first one is on the M-C distance (M: the nearest cluster atom to the carbon atom of CO_2 at the begin of the simulation), the second one a development of a Markov State Model for the evolution of the OCO angle.

M-C distance as function of time

It is difficult to find a good reaction coordinate (RC) for the AIMD simulations. In Figure S32(a-e), we represent the distance between M-C as function of time. The distance oscillates in a range of 1 angstrom around an average value, except in the case of Pd_2Pt_2. The Pd_2Pt_2 simulation shows periodically an abrupt change of the M-C distance, so it represents the umbrella motion we described in the main text.

However, for the other cluster compositions, the difference is not very useful as reaction coordinate. For instance, in the Pd_3Pt simulation, we observe that the cluster opens into a bent-rhomboidal shape. But we do not see it in the M-C distance, because the atoms that move to open the tetrahedral shape are not the ones, we are tracking the distance of. A simular results is also obtained for the Pt_4 or the $PdPt_3$ simulations, for which the M-C distance does not show that the cluster isomerizes.

Our analysis shows that the M-C distance does not represent a descriptive RC. This is first because the CO_2 molecule migrates around the cluster surface and the cluster also modify its structure, but we are tracking only the distance between the nearest metal atom at the start of the simulation to C. All these considerations made us dispense with using the M-C distance as RC for describing the AIMD simulations.

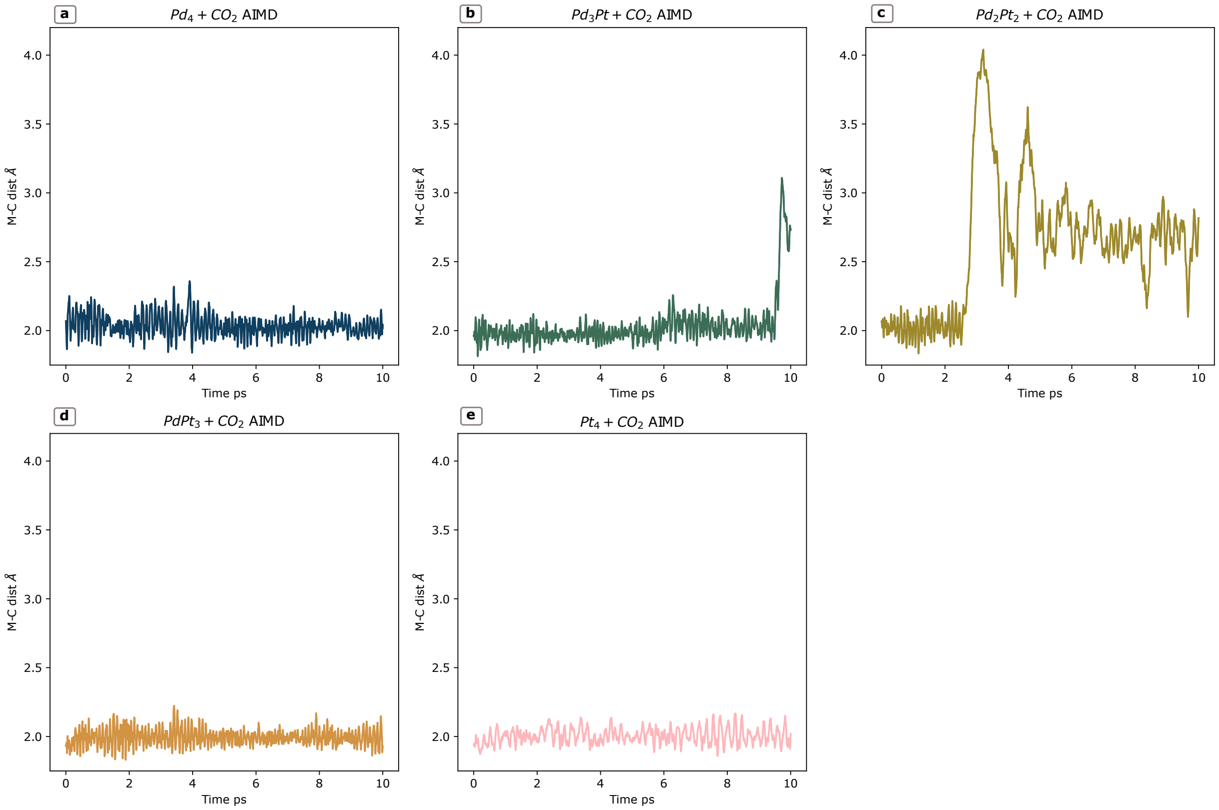

Figure S32: M-C distance as function of time for the PdxPt$_{4-x}$ CO_2 AIMD simulations.

Markov State Model of the OCO angle

The variation of the OCO angle as function of time is reported in Figure S33. To analyze the variation of the OCO angle, we developed a Markov State Model (MSM) by utilizing K-Means for the partitioning the state space. The results did not show any trends for the CO_2 activation or differentiation of this as function of the metal cluster composition, as the OCO angle fluctuates around a 20-degrees range. The script has been written by the authors and the Kmeans algorithm we use is from the scikit-learn Python package[8]. We define the state space, i.e., all the possible values in our system (state is in the mathematical sense) to be the possible values that the OCO angle can take. The OCO angle as function of time is our trajectory. This is how our MSM model works:

- Consider a trajectory. In this case, the trajectory is the OCO angle as function of time.
- Assign N centroids using the Kmeans algorithm to discretize the state space into Voronoi cells centered in the centroids.
- Assign each point of the trajectory to a centroid.
- Build a count matrix for counting the transitions from a Voronoi cell to another.
- Row-normalize the count matrix to obtain a transition probability matrix P

See also a very clear explanation of MSM in the work of Prinz et al.[9]. Please, note that in Figure S33 the color sequence is not relevant, it is a mere coloring for distinguishing the different kmeans clusters. Time step = 5 fs.

Figure S33 shows that, for each cluster composition, the OCO angle of the CO_2 molecule opens and closes for the whole simulation. The only exception is for the simulation Pd_3Pt simulation, in which we see that the CO_2 angle is larger in the second half of the simulation.

The corresponding transition probability matrices are:

$$P_{Pd4} = \begin{Bmatrix} 0.72 & 0.13 & 0.14 \\ 0.23 & 0.77 & 0.00 \\ 0.21 & 0.00 & 0.79 \end{Bmatrix}; \quad P_{Pd3Pt} = \begin{Bmatrix} 0.8 & 0.07 & 0.13 \\ 0.14 & 0.86 & 0.00 \\ 0.14 & 0.00 & 0.86 \end{Bmatrix};$$

$$P_{Pd2Pt2} = \begin{Bmatrix} 0.73 & 0.13 & 0.14 \\ 0.21 & 0.79 & 0.00 \\ 0.23 & 0.00 & 0.77 \end{Bmatrix}; \quad P_{PdPt3} = \begin{Bmatrix} 0.67 & 0.17 & 0.16 \\ 0.3 & 0.7 & 0.01 \\ 0.29 & 0.01 & 0.71 \end{Bmatrix};$$

$$P_{Pt4} = \begin{Bmatrix} 0.83 & 0.09 & 0.08 \\ 0.15 & 0.85 & 0.00 \\ 0.14 & 0.00 & 0.86 \end{Bmatrix}.$$

We thought that the partitioning of the state space (the values of the OCO angle) and the computation of the transition matrix could give some information about the CO_2 behavior. Instead, we see a cyclic behavior between the states: from state 1 (central value), one can go to state 2 or 3 (which is, close or open the angle), and transitions from state 2 to state 3 are not allowed (OCO angle does not change of 50 degrees in 5 fs). The model shows what happens in the simulation (the OCO angle varies in a certain range and does not change abruptly), which means that the model represents well the process. However, the information we obtain is almost obvious.

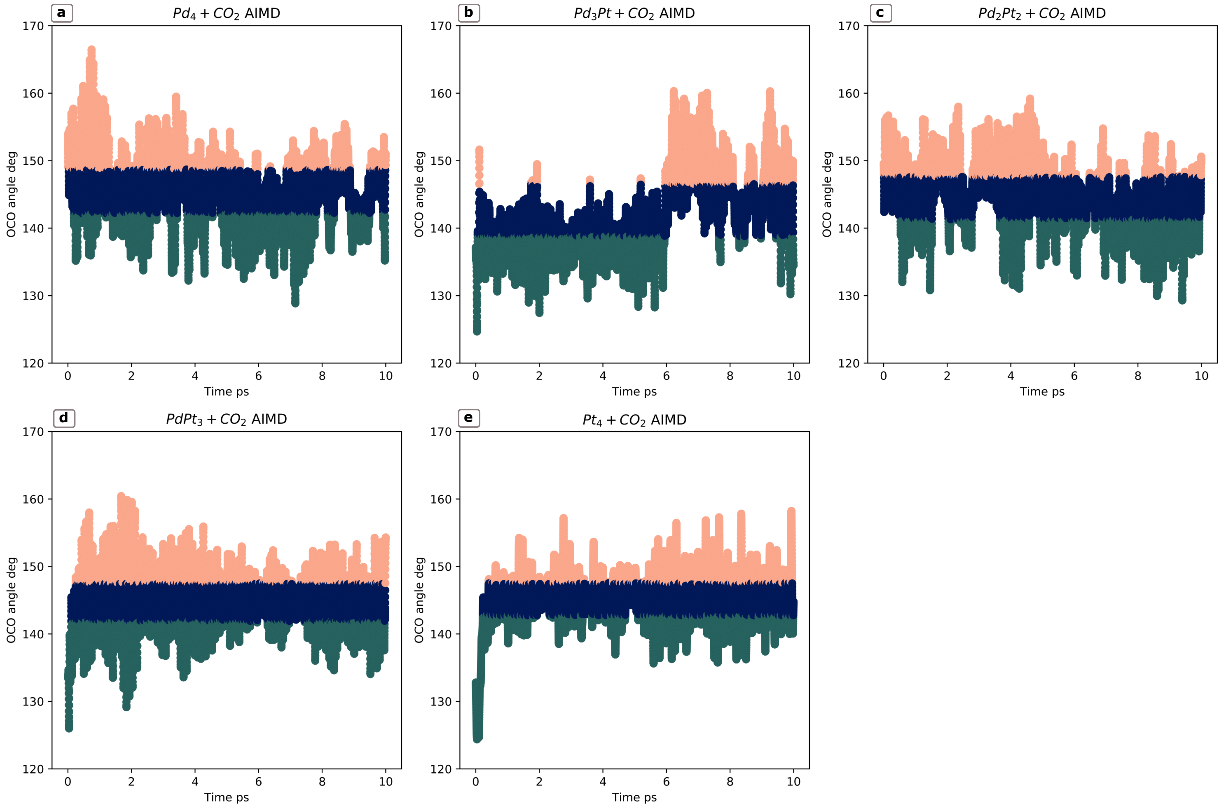

Figure S33: OCO angle as function of time for each Pd_xPt_{4-x} cluster. The color coding in each figure represents the assignment of the trajectory point to one of three centroids by K-means algorithm.

Reduction of the oxide clusters

Reduction of the oxide clusters was investigated by computing the reaction energy (ΔE) of

$$Pd_xPt_{4-x}O + H_2 \longrightarrow Pd_xPt_{4-x} + H_2O$$

The negative reaction energies indicate thermodynamically favoured reduction of the oxide clusters.

Table 4: Reaction energy (ΔE) of the oxide clusters (PBE-D3/def2-TZVP)

Cluster	ΔE (kJ/mol)
Pt_4O	-93
$PdPt_3O$	-112
Pd_2Pt_2O	-172
Pd_3PtO	-165
Pd_4O	-142

Coordinate of the structures in the reaction paths

We report the coordinates of the structures represented in Figure 6, optimized with PBE+D3/def2-TZVP level of theory. All the adducts coordinates are reported from the left to the right as appearing in Figure 6. In case of multiple reaction paths for the same cluster composition, we refer to each reaction path as labelled by the legend of Figure 6. Atomic coordinates are given in Ångstroms.

Pd_4

Pd4
Pd -5.9004753226 1.9860237924 0.9062138566
Pd -4.8769975689 3.5321057880 -0.8660641125
Pd -4.3398122459 0.9533952070 -0.9449130980
Pd -3.2972242626 2.3945731126 0.9047633540
Pd -3.404697580 1.002983174 0.709692580
Pd -4.883561879 2.448867062 -0.859539921
Pd -2.530587960 1.848839625 -1.724861366
Pd -2.870693637 3.613644459 0.192533226
O -1.836341941 -0.365919337 1.652115865
C -1.635938579 -0.045266887 0.481869986
O -0.944371425 -0.103466096 -0.501202370
Pd -3.7029795462 1.0485974110 0.8814007656
Pd -4.7985637252 2.5211439447 -0.9694117441
Pd -2.4632524698 1.4545188740 -1.4071351760
Pd -2.8205796758 3.5107902582 0.2830513081
O -1.8348688990 -0.1942543446 1.4771261569
C -1.6345928484 0.1116576026 0.3118152844

O -0.8576218356 -0.0260327459 -0.6205045948
Pd -4.277073180 1.053138670 0.254290507
Pd -1.727947043 1.914219161 0.464167139
Pd -2.574787622 -0.286967592 1.764456927
Pd -3.378039167 2.013771252 2.501392651
O -3.139196758 0.852413187 -1.885244103
C -2.088755597 1.288033916 -1.447315395
O -0.920394632 1.565072406 -1.701138725
Pd -4.2720833630 0.4617489360 0.2062438265
Pd -1.9540326173 1.9324791521 0.3688800892
Pd -2.5263744578 -0.1661682406 2.1103978920
Pd -3.7791528973 2.0217002793 2.2992138521
O -3.2535411689 1.6259620654 -1.1324484333
C -1.4261044931 1.4885898883 -1.4091017382
O -0.9821715026 1.1908299196 -2.4374744883
Pd -4.2477701833 3.0978463529 0.1022072072
Pd -2.8802170524 0.4436909170 0.2669916676
Pd -1.6794856752 2.6649257029 -0.5305645354
Pd -3.5073433534 1.9128839546 -2.3214273658
O -4.4366233771 1.2799600537 -0.6782315910
C -1.4860519194 -0.3617865084 1.1556521349
O -0.6038623392 -0.8510854728 1.7182153825
Pd4O
Pd -4.2123331635 3.0587378032 0.1436289366
Pd -2.9184371381 0.5040203681 0.1827593660
Pd -1.6353877986 2.7292701270 -0.5309786511
Pd -3.4585673901 1.8305331071 -2.2766170751

O -4.5267143097 1.2767456945 -0.6798171765

Pd_3Pt

Pd3Pt
Pt 0.3806589733 -0.9396129902 0.6334592267
Pd -0.4040067556 1.4867714326 0.5458819284
Pd -0.7978040503 -0.2056267788 -1.5019050090
Pd 1.6878274327 0.6315244365 -0.8940013462
Pt 0.3316188360 -1.1745341284 -1.2996240589
Pd 0.6911486834 1.2337371273 -0.4885141537
Pd 0.0534351409 0.6586765323 -3.0615853786
Pd 2.4726572315 -0.0173522361 -2.0838085271
O -1.1425622536 -1.2086342853 1.8382779133
C -0.7294211460 -0.1188448305 1.9320197364
O -0.3311717923 0.9787959206 2.0727444686
Pt 0.4661035900 -1.3133758198 -1.3211660292
Pd 0.4188391595 1.0724376098 -0.3693840489
Pd 0.0453962615 0.5849417059 -2.9798073163
Pd 2.4650207493 0.1430814446 -1.9688793149
O -1.2607738826 -1.0594830498 1.7249724378
C -0.6735621736 -0.0487706058 1.7908189396
O -0.1179940041 0.9726787152 2.0278752320
Pt -0.0354725613 -1.1920276036 -1.1483558161
Pd 0.5458766424 1.2869386535 -0.1472000136
Pd 0.0739988187 0.7243973629 -2.8397739233
Pd 2.2558724534 -0.1905948153 -1.7406860680

O -0.7708501912 -0.9975606696 0.8306036629

C -0.4391546064 0.1906207188 1.1327275515

O -0.5694219556 0.9344803534 2.0896602066

Pt -0.0694996074 -1.1874231242 -1.0340480235

Pd 0.3755754790 1.2410362998 -0.1697182974

Pd 0.0880090724 0.5521197546 -2.9051638310

Pd 2.2354880885 -0.1803735821 -1.6195413081

O -0.5582790613 -1.1093403436 1.1179797557

C -0.3206839373 0.1088849067 1.3111459106

O -0.4027614340 0.9313500889 2.2063213937

Pt 0.6014359100 -1.2628612350 -0.8523749610

Pd 0.1312889800 1.3027177680 -0.2165351130

Pd 0.7675624070 0.3772560840 -2.7997396130

Pd 2.6212090030 0.3186369140 -0.9734311870

O -0.6811303430 -1.1692979240 0.8776728340

C -0.8212466270 0.0858674930 0.9929694390

O -1.4441273290 0.8543198990 1.7054816010

Pt 0.8549098167 -1.0412547839 -0.4408614761

Pd 0.0790199216 1.3738189765 -0.3726306730

Pd 0.6862760352 0.2381027388 -2.6984456077

Pd 2.7316449156 0.5398181319 -1.1338342033

O -1.3254957937 -1.2632169926 0.6997908881

C -0.8997899071 -0.1437844445 0.9418191467

O -0.9277080883 0.8015726737 1.7143689253

Pt -0.617616084 -0.392593896 -0.553845907

Pd -0.360120373 2.014794473 -1.692593544

Pd 0.418339856 -0.117282590 -2.900271605

Pd 1.816443596 0.764729934 -0.903998045
O -1.186360948 -0.854442676 1.541236662
C 0.017400238 -0.546819886 1.364024745
O 1.086905715 -0.361746358 1.879492694
Pt -0.1090200715 0.2636940481 -0.0875073052
Pd -0.7531112216 1.6212047384 -2.2558607758
Pd 0.7023094379 -0.4872966422 -2.4960909438
Pd 1.6913978885 1.6358635123 -1.4642260204
O -1.4710099106 0.2622427594 1.2429541744
C 0.3565723317 -0.7420701795 1.4107180888
O 0.6823405317 -1.3805517548 2.3115832925
Pt -0.207504178 -0.106579885 0.376730297
Pd -0.335104760 1.480544604 -2.143328562
Pd 0.064299946 -1.295998306 -2.452675637
Pd 1.857301398 0.241503846 -1.213055215
O -1.176548392 -0.171045491 -1.376199340
C 0.592234048 -0.052075129 2.014287437
O 1.109080939 -0.012689640 3.052752021
Pd3Pt II
Pt 0.841451991 -1.721945418 -2.112677219
Pd -0.775270743 -0.645902540 -0.393579138
Pd 0.407497771 0.764402148 -2.433008056
Pd 1.867303382 -0.134642701 -0.363231456
O -1.558050467 0.606121063 1.389551952
C -0.351200428 0.610541570 1.158320780
O 0.743259495 1.028063878 1.488668137
Pd3PtO

Pt -0.2178404306 -0.1123427441 0.2843716943
Pd -0.3386791866 1.4886836537 -2.1220463927
Pd 0.0614745332 -1.3082351728 -2.4218925635
Pd 1.8963675831 0.2502843385 -1.2045867652
O -1.1988785991 -0.1699653753 -1.3443743728

Pd_2Pt_2

Pd2Pt2
Pt -3.2488980467 -0.0743361140 1.0801471069
Pd -1.7192310312 -0.0643154799 -1.0113043673
Pd -1.8434374740 2.0813339632 0.7550622050
Pt -3.7986778480 1.4825006307 -0.8445499446
Pd2Pt2 I
Pt -1.556376876 0.606012850 1.666451047
Pd 0.466299401 1.882655469 0.771416994
Pd -2.002285494 2.841188996 0.491694134
Pt -1.134199318 0.920421686 -1.168280620
O -2.153356515 -1.091241001 0.555757634
C -1.893387635 -0.793052435 -0.666179902
O -2.028621563 -1.407219566 -1.720896288
Pt -2.3190772678 1.0753982246 1.5112639678
Pd 0.2241014706 1.0160214782 0.7820811303
Pd -1.3497601001 2.8938505417 -0.2724316317
Pt -1.6416591813 0.4307481249 -1.0587454596
O -1.6318877815 -1.7085202550 1.0517161877

C -1.7118759644 -0.5887999916 0.6658319804

O -1.7240962755 0.0633666772 -2.7905250749

Pt -1.1148513000 0.2198622000 1.1157863000

Pd 0.4770489000 2.0220497000 0.2240872000

Pd -2.1487243000 2.4183865000 0.0153161000

Pt -0.8909870000 0.7360933000 -1.5163017000

O -3.3306910000 -1.7147394000 1.6647404000

C -2.4473495000 -0.9880354000 1.4951665000

O -0.5822353000 0.5955743000 -3.2444210000

Pt -2.0662080901 0.5448840500 1.1273698947

Pd 0.8071085665 1.8587953167 -0.5264851044

Pd -1.5602879925 2.8529227683 0.1146527219

Pt -1.3281508227 0.6698756253 -1.3153800937

O -3.0890757538 -2.2260653245 1.5364139918

C -2.6807477840 -1.1470046024 1.4407086811

O -0.1215430234 0.6829250666 -2.6508042913

Pt -1.3072514315 -0.4304612956 0.2482737499

Pd -1.5431059446 1.4350280714 -1.9616015642

Pd -1.2707665508 2.1565569621 0.7256608825

Pt -3.4714962257 0.9921763486 -0.1667222434

C 0.0778340681 -1.2735505490 1.1104798772

O 0.9458462597 -1.7383349912 1.7213722892

O -3.5276601752 1.4969124537 -1.9520059911

Pd2Pt2O I

Pt -1.3356992414 -0.3923976647 0.2472769487

Pd -1.5286044841 1.4123484035 -1.9574833398

Pd -1.2768818447 2.1631487617 0.6882868867

Pt -3.4712534500 0.9228872214 -0.1683652916
O -3.5078413076 1.5442258183 -1.9161103703
Pd2Pt2 II
Pt -2.152426011 -0.233592573 0.137319169
Pd -2.555295686 0.799046853 -2.342641617
Pd -1.427014741 2.330377122 -0.434612122
Pt -3.876771091 1.582389687 -0.295372288
O -0.552248406 -1.586980315 0.734906486
C -0.278717137 -0.368608905 0.892319515
O 0.540544071 0.436134132 1.238042858
Pt -2.6425508587 0.0881211226 0.7552729557
Pd -1.2533245264 0.6277401227 -1.3526212313
Pd -2.2550318748 2.6847358490 0.3215741251
Pt -3.7997046641 1.2920549942 -1.1639453143
O -0.7924240401 -1.1224232115 1.2792947626
C -0.3752993610 -0.4543897990 0.3232589655
O 0.6653745249 -0.1495866781 -0.2418341634
Pt -2.9172448775 -0.1472067429 0.5796512424
Pd -1.1515391445 0.7090223357 -1.1020927675
Pd -2.4942078756 2.4546486842 0.6901363628
Pt -3.7290218597 1.3746476505 -1.2889278927
O -0.9633314499 -1.2750018426 1.0600745791
C -0.3467834604 -0.6491480088 0.1900445525
O 0.7621597676 -0.4823010761 -0.2960213767
Pd2Pt2 III
Pt -3.996333277 0.239606428 1.024770144
Pd -1.954247391 -0.508953250 -0.406450288

Pd -1.888445764 1.732640832 1.336485852

Pt -3.269622405 1.734799112 -0.884736956

O 0.140903112 -0.919651775 -1.204171699

C 0.064126097 -0.062103622 -0.324668072

O 0.601691627 0.742428276 0.388734019

Pt -4.0765070804 0.5713409556 1.2526776883

Pd -2.1059658516 -0.6505864618 -0.0716674004

Pd -1.5858941523 1.5782934899 1.0343516800

Pt -3.3119704669 1.5977801245 -0.9214449288

O 0.7655249286 -0.8863968695 -1.0788437548

C -0.2272656885 -0.5471297388 -0.5896409021

O 0.1131200110 1.0990195001 0.6001763178

Pt -3.8387366517 0.7284239780 0.8031583756

Pd -1.7249075982 -0.4544555214 1.8107827029

Pd -1.6292999099 2.1281654584 0.9171630553

Pt -2.0422680016 0.0651845005 -0.9315846974

O 0.2347486704 -0.7100392658 -2.7061594783

C -0.6682125701 -0.4073433858 -2.0434509846

O -1.0009920521 1.1931543351 2.4648363372

Pt -3.4772727000 0.2739080000 -1.4260125000

Pd -0.8580073000 -0.1543861000 -1.5084342000

Pd -1.9527439000 2.2994449000 -0.9086164000

Pt -2.2369311000 -0.2274761000 0.7835208000

O -0.4716347000 -0.8694223000 3.1076162000

C -1.1883425000 -0.6222720000 2.2293804000

O -0.4812091000 1.6630500000 -1.9412467000

Pt -3.5781229217 0.2692733939 0.9567927446

Pd -0.9500959602 0.1149741390 1.2841315832
Pd -2.3751535910 2.3384304587 1.9695327070
Pt -2.0307586103 -0.0950672341 -1.0512299964
O -0.0872251808 -1.0877172008 -3.0889414208
C -0.8535834819 -0.6832265411 -2.3184627303
O -0.8179827588 1.4361557833 2.6321908054
Pt -2.4682961470 -0.6354641955 1.9677770702
Pd -2.7877108130 1.3845807423 0.2781464433
Pd -0.8185797062 1.3458165439 2.1738116719
Pt -1.3291504034 -0.7415020114 -0.3306742020
O -0.3527642519 -0.8174991299 -3.1546656452
C -0.7140848391 -0.7914128219 -2.0527047893
O -2.0433075395 2.6333594723 1.5100211511
Pt -2.4682961470 -0.6354641955 1.9677770702
Pd -2.7877108130 1.3845807423 0.2781464433
Pd -0.8185797062 1.3458165439 2.1738116719
Pt -1.3291504034 -0.7415020114 -0.3306742020
O -0.3527642519 -0.8174991299 -3.1546656452
C -0.7140848391 -0.7914128219 -2.0527047893
O -2.0433075395 2.6333594723 1.5100211511
Pd2Pt2O III
Pt -1.8406365519 -0.3826041606 0.9370575378
Pd -2.4196949253 1.5224392286 -2.6058018465
Pd -1.0555497032 1.6570313440 -0.2725118060
Pt -3.6012573311 0.6583366693 -0.4674559834
O -0.9627886885 2.5622331186 -1.9435011019

$PdPt_3$

PdPt3
Pt -1.1322369399 -0.3264646508 0.6584271164
Pt 0.0998292918 1.1811441307 -1.0503398676
Pt 1.0088293386 -1.1417186398 -0.4829826501
Pd 1.2655053094 0.6908571599 1.3126636012
PdPt3 I
Pt -1.787585760 0.046996083 -0.788432170
Pt -0.566423491 1.712185528 -2.325519634
Pt 0.477132006 -0.600012889 -1.875733998
Pd 0.426933157 1.365485733 0.027932874
O 1.104542959 0.273532412 2.069044532
C 0.990422256 -0.860082836 1.754050805
O 0.905221872 -2.000704030 1.527035590
Pt -0.5306370870 1.7045106690 -2.2850373165
Pt 0.4447043030 -0.6470308979 -1.9261654877
Pd 0.4727065640 1.2657952862 0.0517055046
O 1.0726626211 0.3062148382 2.1563007226
C 0.9635388594 -0.7921533068 1.7187528884
O 0.8981844286 -1.9224716980 1.4322469733
Pt -2.0092208200 0.3061761870 -0.9839023684
Pt -0.0726223266 1.2855397172 -2.3424877808
Pt 0.2611450293 -0.9513869920 -1.2286283452
Pd 0.0509375328 1.1598971546 0.5375808969
O 1.2982651961 0.0223312450 2.4088051654
C 0.9475897961 -0.3905823590 1.3162815815

O 1.0638482922 -1.4706376528 0.6543455505

Pt -1.9214185868 0.2405203134 -0.8254951200

Pt -0.2662174325 1.3508665139 -2.3914293402

Pt 0.3996093357 -0.8719878453 -1.2847190682

Pd 0.2615095775 1.1969746785 0.3857903506

O 1.1319374461 0.0154421649 2.4391626392

C 0.9259139362 -0.4435259724 1.3283454515

O 1.0098829238 -1.5330407530 0.7155511872

Pt -0.965221391 0.139735723 -1.062751042

Pt 0.119771134 2.414178292 -0.441484081

Pt 1.086991922 0.943553335 -2.306664103

Pd 1.433040094 0.219390320 0.416026132

O -1.033343803 -1.145202285 0.640136479

C 0.132138067 -1.046375382 1.129816259

O 0.776867978 -1.587879002 2.013297357

Pt 0.9372705454 1.2467164575 -2.4451024040

Pt 0.0997721997 2.4041654710 -0.3440637165

Pt -0.8737675622 0.0814918166 -0.9669597388

Pd 1.4840721399 0.0815341466 0.0004819272

O 0.5054863156 -1.6068801138 2.3273602585

C 0.5274974523 -1.0112147136 1.3309175583

O -1.1056923907 -1.2283402643 0.2894882152

Pt -0.5214461961 1.5068308092 -1.0407043525

Pt 1.1312490162 1.8097354410 0.9616012930

Pt -1.8333597068 -0.4151356783 -1.9603240881

Pd 0.7180589145 -0.6413915777 -0.2761494468

O 1.2464924453 -0.5574933013 2.5862969798

C 1.1063275038 -0.0191746312 1.5422505147

O -0.3822351769 -1.5339793617 -1.7331120002

Pt -0.6765722869 1.4159250776 -0.5121155594

Pt 1.5334668783 1.6822321542 0.9987281858

Pt -1.7042571269 -0.6234344627 -1.5537008421

Pd 1.0828861272 -0.5038230547 -0.5046937197

O -0.0076773347 -0.4847732087 2.2036731954

C 0.5378645017 0.1043640362 1.3380725331

O -0.1346239587 -1.5610988419 -1.7581048932

Pt 0.7045936 0.8643738 -0.0989825

Pt -0.4371837 -0.0946589 2.1122072

Pt 2.0729065 -0.2432672 -2.0223384

Pd 0.6724567 -1.6636255 0.2544029

O -2.0619953 1.6789445 0.4776306

C -1.0518249 1.1009659 0.6966821

O 1.9161291 -1.9185785 -1.2689909

Pt 0.2449921155 0.6801405222 -0.3617265282

Pt -0.3670352979 -0.3882829516 1.8686307200

Pt 2.1990938895 -0.3293835317 -1.7033157576

Pd 1.2467309155 -1.7719971792 0.4070462961

O -2.1076446339 2.1024419354 0.8546310820

C -1.2099755006 1.4654623595 0.4767417000

O 1.9331810118 -2.1435185547 -1.4096476124

Pt -0.4562867 -1.1479682 -0.4508976

Pt 1.9353515 -1.3781498 0.4578110

Pt 0.8688020 0.9423299 0.0825679

Pd 2.3521942 0.5158414 2.3694195

O -2.9229066 -0.5301915 -2.0204425

C -1.9814245 -0.7945868 -1.4003875

O 1.9598411 2.0096870 1.1457982

Pt 1.4088020769 1.2236170797 -2.2967036896

Pt 0.3193610196 2.4134507849 -0.3829229213

Pt -0.4223423213 -0.1045237955 -0.9173452454

Pd 1.7173126485 0.2527478087 0.2833669472

O 0.9873342987 -0.9590925512 1.4511857229

C -0.8440470930 -1.1004837498 0.6787932257

O -1.5210583293 -1.7531009768 1.3650915604

PdPt3 II

Pt 0.3399641 0.0364026 0.4566851

Pt 2.2171284 0.8571714 -1.1188308

Pt 2.0545877 -1.6152631 -0.5379945

Pd 3.1688274 0.0105469 1.1292228

O -2.2443211 -0.2296394 -1.0581645

C -1.6058055 0.0309310 -0.0840778

O -1.6513731 0.4242736 1.1260048

Pt 0.4097351971 1.1002877962 0.4275453197

Pt 2.5993802173 0.9077671456 -1.0423340825

Pt 0.9919930595 -1.1083087436 -1.0642937937

Pd 2.4861996078 -0.5873144225 1.0597566244

O -1.0230255890 -1.1850422951 -0.4638922896

C -1.1191211985 -0.0952594537 0.1898070606

O -2.0110452942 0.4887697729 0.8005100612

Pt 1.1175418 -0.1566986 -0.9646327

Pt 2.4502196 0.1232305 1.2602324

Pt -0.0337327 0.2610315 1.7880049
Pd 1.0919225 2.1972649 0.4370947
O -1.3282971 -0.6638795 0.4109641
C -0.7108549 -0.7264132 -0.7074015
O -1.0365563 -1.0971356 -1.8358848
Pt 2.3570244490 0.0114681092 1.2785309207
Pt 0.8996720738 -0.1242025973 -0.9298687941
Pt -0.1907971176 0.6353574400 1.4254748499
Pd 1.5640448427 2.2062043916 0.2268245808
O -1.5208683795 -1.5347844448 0.3067462914
C -0.7806635223 -0.6773413211 -0.0396027185
O -0.6821152462 -0.4494475776 -1.9337236302
Pt 1.9353515 -1.3781498 0.4578110
Pt 0.8688020 0.9423299 0.0825679
Pt -0.4562867 -1.1479682 -0.4508976
Pd 2.3521942 0.5158414 2.3694195
O -2.9229066 -0.5301915 -2.0204425
C -1.9814245 -0.7945868 -1.4003875
O 1.9598411 2.0096870 1.1457982
PdPt3O
Pt -0.3732986515 -1.1029593923 -0.5100608227
Pt 1.8687491916 -1.4608075153 0.5418336396
Pt 0.8270735219 0.9429140135 0.1235968027
Pd 2.4307849097 0.5146442002 2.2839825766
O 1.9065931282 2.0479489939 1.1653468039
PdPt3 III
Pt -1.787585760 0.046996083 -0.788432170

Pt -0.566423491 1.712185528 -2.325519634

Pt 0.477132006 -0.600012889 -1.875733998

Pd 0.426933157 1.365485733 0.027932874

O 1.104542959 0.273532412 2.069044532

C 0.990422256 -0.860082836 1.754050805

O 0.905221872 -2.000704030 1.527035590

Pt -1.0595869001 -0.5352047340 -0.6066301516

Pt -1.1176866368 1.5046217965 -2.1627116227

Pt 0.8370096622 -0.1552959983 -2.3490780569

Pd 0.6558620981 1.5565787911 -0.2765216982

O 1.4516265198 0.1205554111 1.9722881502

C 0.7839129742 -0.8332725882 1.8134032794

O 0.1644059826 -1.8285468781 1.7463963998

Pt 0.033817129 0.205132937 -0.461283745

Pt 0.918740318 2.851592795 -0.287505296

Pt 2.503938716 0.893478742 -0.307754739

Pd 0.967692256 1.246784216 1.749719330

O -1.713008829 -1.002982532 -0.651434829

C -0.749058636 -1.614694709 -0.082231140

O -0.411877954 -2.641910449 0.428867419

Pt 2.5993585127 1.3947751459 -0.7285717936

Pt 0.5142338188 2.4454653476 0.2987715794

Pt 0.3853542970 0.4590132477 -1.4027492688

Pd 0.6904847054 -0.0525947241 1.2463537776

O -0.9521485279 -2.0015798552 1.2962734445

C -0.6682185115 -1.2010648947 0.4229276960

O -0.9901184046 -1.0112838371 -0.7950267751

Pt 1.086991922 0.943553335 -2.306664103
Pt 0.119771134 2.414178292 -0.441484081
Pt -0.965221391 0.139735723 -1.062751042
Pd 1.433040094 0.219390320 0.416026132
O 0.776867978 -1.587879002 2.013297357
C 0.132138067 -1.046375382 1.12981625
O -1.033343803 -1.145202285 0.640136479

Pt_4

Pt4 I
Pt -3.2612596652 -0.0878540274 1.0919441564
Pt -1.6634285303 -0.0105280058 -0.9450718264
Pt -1.8250499807 2.0015760747 0.6815567115
Pt -3.8605062239 1.5219889584 -0.8490740415
Pt -1.8084437859 0.1458301112 0.1723660880
Pt -2.2129229987 1.2057330899 -2.1580559392
Pt -2.2204438243 2.6934454401 -0.0591892611
Pt -4.2705746829 1.2819957054 -0.6564110430
O -0.9209430313 -1.2668285636 1.4627363153
C -0.0455044523 -0.5918730838 0.8159465858
O 1.1244746755 -0.4326236991 0.6396658541
Pt -2.2508326971 0.2549280429 0.9812144658
Pt -1.2524157399 1.1147078611 -1.6057462557
Pt -2.3365430899 2.7493583983 0.0506412690
Pt -3.7988561185 0.9308179791 -1.0050918281
O -0.3964487961 -0.9930944947 1.7143181634

C -0.4927257874 -0.5268699286 0.5646750215

O 0.1312188290 -0.4654050581 -0.5031191358

Pt -0.829790999 1.249085117 -0.874317680

Pt -2.897209346 0.000521596 0.926521672

Pt -2.597510554 2.547524503 0.627014814

Pt -3.459865615 1.095832246 -1.340813542

O -1.065538902 -0.989338075 0.874601057

C -0.298948516 -0.368273283 0.055301649

O 0.846934932 -0.576586105 -0.338343970

Pt -0.9391741229 1.3984435990 -0.4391180366

Pt -2.6113055121 0.1547746457 0.9079958168

Pt -3.0739363752 2.7726931103 0.4810082566

Pt -3.4543912052 1.1111680438 -1.3895830098

O -1.2685253129 -1.1141667744 0.9584827614

C -0.0152578854 -0.3060497473 -0.2281715073

O 0.9301814137 -0.9704059772 -0.3917294811

Pt -1.6539261000 -0.8024410000 0.6000582000

Pt -2.7001197000 1.8605692000 -2.6070873000

Pt -3.4969323000 0.6945478000 -0.4285398000

Pt -0.9530416000 1.4027099000 -0.4237038000

O -1.2000362000 2.6818645000 -1.8217913000

C -0.4333016000 -1.4383892000 1.8043990000

O 0.3407586000 -1.7605352000 2.6021201000

Pt -1.9210633373 -0.3264024510 0.6445478740

Pt -1.7863113034 0.4696190551 -2.0411378109

Pt -3.7812586833 1.1219235793 -0.4895248951

Pt -1.5630298536 2.4011666019 -0.0229380315

O -1.0183905774 2.2111504262 -1.8480984740
C -0.5461405301 -1.2664344000 1.4099426563
O 0.3558176851 -1.8296247116 1.8705092811
Pt -1.8952958081 -0.3578920896 0.5735961170
Pt -1.9471553822 0.5846439020 -2.2511058416
Pt -3.7694629209 1.1086618251 -0.5180679188
Pt -1.4492346043 2.2491615237 0.0295238261
O -0.9933578929 2.1668195462 -1.8291240019
C -0.5547464889 -1.2153672344 1.4784653645
O 0.3167677972 -1.7164326731 2.0553835546
Pt -0.7278329067 0.1278857046 -0.2596312741
Pt -2.3346722393 1.4235893172 -2.6728789215
Pt -3.5445394724 0.7915774319 -0.5831948099
Pt -2.0292248864 1.9501763166 1.0394097501
O -0.6757974637 0.8125567768 -2.0747577282
C -0.5326352272 -0.7666596836 1.3421794903
O -0.4073685011 -1.3486034714 2.3333208840
Pt -0.8107615 -0.7368461 -0.1958058
Pt -2.5092356 1.2378896 -1.9010352
Pt -2.1656933 1.3835716 0.5348122
Pt -3.1051207 3.3447646 -0.7613370
O -1.7568642 -0.4991643 -1.9472554
C 0.0978673 -1.0116655 1.3690601
O 0.6766459 -1.1827629 2.3559920
Pt4 II
Pt -0.7541274064 1.1499953806 -0.2116995705
Pt -3.0818785282 0.0167250600 0.2694917518

Pt -2.8238668603 2.6523491855 0.1341277584
Pt -4.8387378095 1.4106369268 -0.7580298547
O -0.9951126539 -0.5683286430 0.7640585556
C 0.4452638563 -0.3458000666 -0.1349659993
O 1.2961340019 -1.1337104434 -0.2309734413
Pt4O
Pt -0.8114154649 -0.6733567634 -0.2622226641
Pt -2.2122538197 1.3550773776 0.5469279193
Pt -3.0730651092 3.3624618999 -0.7607038025
Pt -2.4993076390 1.2290189954 -1.8893827534
O -1.7516332671 -0.5429861095 -1.9052398993

References

(1) Ha, M.-A.; Dadras, J.; Alexandrova, A. Rutile-Deposited Pt–Pd clusters: A Hypothesis Regarding the Stability at 50/50 Ratio. *ACS Catalysis* **2014**, *4*, 3570–3580.

(2) Alvarez-Garcia, A.; Flórez, E.; Moreno, A.; Jimenez-Orozco, C. CO2 Activation on Small Cu-Ni and Cu-Pd Bimetallic Clusters. *Molecular Catalysis* **2020**, *484*, 110733.

(3) Lee, T. J.; Taylor, P. R. A Diagnostic for Determining the Quality of Single-Reference Electron Correlation Methods. *International Journal of Quantum Chemistry* **1989**, *36*, 199–207.

(4) Wang, J.; Manivasagam, S.; Wilson, A. K. Multireference Character for 4d Transition Metal-Containing Molecules. *Journal of Chemical Theory and Computation* **2015**, *11*, 5865–5872, PMID: 26642991.

(5) Dai, D.; Balasubramanian, K. Electronic structures of Pd4 and Pt4. *The Journal of Chemical Physics* **1995**, *103*, 648–655.

(6) Piecuch, P.; Włoch, M. Renormalized coupled-cluster methods exploiting left eigenstates of the similarity-transformed Hamiltonian. *The Journal of Chemical Physics* **2005**, *123*, 224105.

(7) Wang, X.; Pan, J.; Wei, H.; Li, W.; Zhao, J.; Hu, Z. CO2 Activation and Dissociation on In2O3(110) Supported PdnPt(4-n) (n = 0–4) Catalysts: a Density Functional Theory Study. *Phys. Chem. Chem. Phys.* **2021**, *23*, 11557–11567.

(8) Pedregosa, F. et al. Scikit-learn: Machine Learning in Python. *Journal of Machine Learning Research* **2011**, *12*, 2825–2830.

(9) Prinz, J.-H.; Wu, H.; Sarich, M.; Keller, B.; Senne, M.; Held, M.; Chodera, J. D.; Schütte, C.; Noé, F. Markov Models of Molecular Kinetics: Generation and Validation. *The Journal of chemical physics* **2011**, *134*, 174105.
